# The Effect of Dialysate Bicarbonate Concentration or Oral Bicarbonate Supplementation on Outcomes in Patients on Maintenance Dialysis: A Systematic Review and Meta-Analysis

**DOI:** 10.1177/20543581251356182

**Published:** 2025-07-31

**Authors:** Ashlee M. Azizudin, Samuel A. Silver, Amit X. Garg, Zoe K. Friedman, Andrea C. Cowan, Catherine M. Clase, Amber O. Molnar

**Affiliations:** 1 Department of Health Research Methods, Evidence, and Impact, McMaster University, Hamilton, ON, Canada; 2 Division of Nephrology, Department of Medicine, Queen’s University, Kingston, ON, Canada; 3 Division of Nephrology, Department of Medicine, Western University, London, ON, Canada; 4 Queen’s University, Kingston, ON, Canada; 5 Division of Nephrology, Department of Medicine, McMaster University, Hamilton, ON, Canada

**Keywords:** bicarbonate, hemodiafiltration, hemodialysis, peritoneal dialysis, systematic review

## Abstract

**Background:**

Metabolic acidosis is a common complication of kidney failure that is treated with bicarbonate supplementation. The addition of bicarbonate to the dialysis solution and oral bicarbonate supplementation are used to treat metabolic acidosis in patients receiving dialysis, but the treatment approach that is best for patient health remains unknown.

**Objective:**

The purpose of this study was to determine whether the concentration of dialysate bicarbonate or the use of oral bicarbonate supplementation alters the risk of mortality, hospitalizations, cardiovascular and nutritional outcomes, and laboratory measurements in patients treated with maintenance dialysis.

**Design:**

Systematic review and meta-analysis.

**Setting:**

Any country of origin.

**Patients:**

Adult patients (≥18 years) receiving maintenance dialysis.

**Measurements:**

Extracted data included demographic characteristics and outcomes such as mortality, hospitalizations, cardiovascular events, surrogate markers of nutrition, and pre-dialysis and post-dialysis levels of serum bicarbonate, pH, calcium, potassium, and parathyroid hormone.

**Methods:**

We searched MEDLINE, Embase, CENTRAL, and Google Scholar through October 7, 2024 for studies examining dialysate bicarbonate concentration and/or oral bicarbonate supplementation in adults undergoing maintenance dialysis. Meta-analysis was performed for pre-dialysis serum bicarbonate and for pre-dialysis and post-dialysis calcium and potassium.

**Results:**

We identified 37 studies (n = 24,782 patients) with patients treated with hemodialysis (13 randomized trials, 10 non-randomized interventional studies, 14 observational studies) and 4 studies (n = 347 patients) with patients receiving peritoneal dialysis (3 randomized trials, 1 non-randomized interventional study). No randomized trials reported mortality or hospitalizations in hemodialysis patients. Studies reporting cardiovascular outcomes (n = 20) were small with inconsistent results. Most studies reporting nutritional outcomes (n = 21) reported no significant differences with dialysate bicarbonate concentration or oral bicarbonate supplementation but were small in sample size (largest study n = 200). Meta-analysis of parallel-group randomized trials comparing dialysate bicarbonate >35 mmol/L with ≤35 mmol/L found a mean difference of 3.5 mmol/L (95% confidence interval [CI] −0.6 to 7.7) in pre-dialysis serum bicarbonate.

**Limitations:**

Non-English and gray literature were excluded. Most studies were small or observational in nature, and heterogeneity further limited the ability to perform meta-analysis of outcomes such as mortality, hospitalizations, and cardiovascular outcomes.

**Conclusions:**

The evidence for the effect of higher vs lower dialysate bicarbonate concentration and oral bicarbonate supplementation on clinical outcomes in dialysis patients is very uncertain. There is a need for large, high-quality randomized controlled trials in this area.

## Abrégé

**Contexte:**

L’acidose métabolique, une complication fréquente de l’insuffisance rénale, est traitée par une supplémentation en bicarbonate. Présentement, les patients sous dialyse sont traités par l’ajout de bicarbonate au dialysat et la prise de suppléments oraux, mais l’approche thérapeutique la plus adaptée à la santé du patient reste inconnue.

**Objectif:**

Cette étude visait à déterminer si la concentration en bicarbonate du dialysat ou la prise de suppléments oraux de bicarbonate chez les patients sous dialyse chronique avait une incidence sur le risque de mortalité, les hospitalisations, les résultats cardiaques et nutritionnels, et les mesures de laboratoire.

**Conception:**

Revue systématique et méta-analyse.

**Cadre:**

Tous les pays d’origine.

**Sujets:**

Patients adultes (≥18 ans) sous dialyse chronique.

**Mesures:**

Les caractéristiques démographiques et les issues cliniques telles que la mortalité, les hospitalisations, les événements cardiovasculaires, les marqueurs substituts de la nutrition, de même que les taux sériques de bicarbonate, de calcium, de potassium, d’hormone parathyroïdienne et le pH avant et après la dialyse.

**Méthodologie:**

Nous avons consulté MEDLINE, Embase, CENTRAL et Google Scholar jusqu’au 7 octobre 2024 à la recherche d’études examinant la concentration de bicarbonate dans le dialysat et/ou la prise de suppléments oraux de bicarbonate chez des adultes sous dialyse chronique. Une méta-analyse a été réalisée pour le taux de bicarbonate sérique pré-dialyse, ainsi que pour les taux de calcium et de potassium pré- et post-dialyse.

**Résultats:**

Nous avons identifié 37 études (n = 24 782 patients) portant sur des patients sous hémodialyse (13 essais randomisés, 10 études interventionnelles non randomisées, 14 études observationnelles) et 4 études (n = 347 patients) portant sur des patients sous dialyse péritonéale (3 essais randomisés, 1 étude interventionnelle non randomisée). Aucun des essais randomisés ne rapportait de mortalité ou d’hospitalisation chez les patients hémodialysés. Les études faisant état de résultats cardiovasculaires (n = 20) étaient de petite taille et présentaient des résultats contradictoires. La plupart des études faisant état de résultats nutritionnels (n = 21) ne rapportaient aucune différence significative pour la concentration de bicarbonate du dialysat ou la prise de suppléments oraux de bicarbonate, mais leurs échantillons étaient de petite taille (étude la plus importante: n = 200). La méta-analyse des essais randomisés en groupes parallèles comparant la concentration en bicarbonate du dialysat (>35 mmol/l par rapport à ≤35 mmol/l) a révélé une différence moyenne de 3,5 mmol/l (intervalle de confiance à 95% [IC] −0,6 à 7,7) pour le taux sérique pré-dialyse.

**Limites:**

L’étude excluait la littérature grise et les études dans une autre langue que l’anglais. La plupart des études étaient de petite taille ou de nature observationnelle. L’hétérogénéité a limité davantage la capacité de réaliser une méta-analyse sur les résultats comme la mortalité, les hospitalisations et les résultats cardiovasculaires.

**Conclusion:**

L’effet d’une concentration plus élevée ou plus faible de bicarbonate dans le dialysat et d’une supplémentation orale en bicarbonate sur les résultats cliniques des patients dialysés ne peut être prouvé avec certitude. Des essais contrôlés randomisés de haute qualité et de plus grande envergure sont nécessaires dans ce domaine.

## Introduction

Kidney failure leads to metabolic acidosis, which is associated with adverse patient outcomes, including malnutrition, bone disease, hospitalizations, and increased mortality. Metabolic acidosis contributes to protein-energy wasting, which is common among patients receiving maintenance dialysis, and is associated with increased morbidity and mortality.^1^ For patients receiving hemodialysis (HD) or peritoneal dialysis (PD), metabolic acidosis is treated primarily by the addition of bicarbonate to the dialysate; however, daily oral sodium bicarbonate supplementation may also be used. Guidelines recommend a serum bicarbonate concentration ≥22 mmol/L, measured pre-dialysis in patients treated with HD, which is based on low quality evidence that suggests an improvement in measures of nutrition and bone metabolism with correction of metabolic acidosis.^1,2^ However, observational data from the international Dialysis Outcomes and Practice Patterns Study (DOPPS) showed that a higher (vs lower) concentration of dialysate bicarbonate concentration was associated with a higher risk of hospitalizations and death.^3^ This association may be causal, potentially explained by an increased risk of significant intra-dialysis and post-dialysis metabolic alkalosis with high dialysate bicarbonate concentrations. However, this result may be confounded (i.e., higher observed mortality with higher dialysate bicarbonate concentration may be driven by differences in age, comorbidities, and central venous catheter use).^3^

The optimal concentration of dialysate bicarbonate and whether oral bicarbonate supplementation should be used remain uncertain. The purpose of this systematic review and meta-analysis was to determine whether the concentration of dialysate bicarbonate or the use of oral bicarbonate supplementation alters the risk of mortality, hospitalizations, cardiovascular and nutritional outcomes, and laboratory measurements in patients treated with maintenance dialysis.

## Methods

This systematic review is reported in accordance with the Preferred Reporting Items for Systematic Reviews and Meta-Analyses (PRISMA) criteria^4^ (Supplemental Appendix A), with the exception of prospective protocol registration. We support protocol registration in our future research to prevent unintended duplication and to increase transparency.

### Study Selection

Eligible populations were adults ≥18 years undergoing maintenance dialysis (any modality) for the treatment of kidney failure. Studies with at least 2 comparison groups evaluating dialysate bicarbonate concentration or oral sodium bicarbonate supplementation were included. Outcomes included the following: mortality, hospitalizations, cardiovascular outcomes (e.g., myocardial infarction, stroke, blood pressure (BP), intradialytic hypotension), surrogate markers of nutrition (e.g., serum albumin, body mass index (BMI)), pre-dialysis and post-dialysis serum bicarbonate, pH, calcium, potassium, and parathyroid hormone (PTH). Additional eligibility criteria were English language and articles that were publicly available or could be ordered through our library system. Publications in the form of consensus statements, protocols, abstracts, conference proceedings, letters, or editorials were excluded. Case studies, case series, studies with less than 20 patients, studies comparing acetate dialysate versus bicarbonate dialysate and studies performed on animals or conducted in vitro were also excluded.

### Searching and Screening

MEDLINE, Embase, CENTRAL, and Google Scholar were searched from inception to October 7, 2024 inclusive. The search strategy was developed with the assistance of a librarian and included Medical Subject Headings and keywords that reflect terms commonly used in the literature to refer to dialysis and bicarbonate treatment in patients with kidney failure (see Supplemental Appendix B). Using Covidence software (Covidence, Australia), AOM and AMA screened articles retrieved from the search, first by titles and abstracts, and subsequently by full text to confirm eligibility for inclusion in the review. Disagreements were resolved through discussion between the two reviewers in accordance with the eligibility criteria.

### Data Extraction and Synthesis

Data extraction was performed in duplicate by AMA and ZKF and verified by AOM using a standardized data extraction form developed and piloted on Covidence software (see Supplemental Appendix C for data abstraction form). When data were available only in figures and not numerically reported, WebPlotDigitizer software (Automeris, US), which has demonstrated high reliability and validity, was used.^5,6^ Where appropriate, data were pooled using meta-analysis; otherwise, results were summarized narratively.

### Assessment of Study Quality

Study quality was assessed independently and in duplicate by AMA and ZKF and verified by AOM. The risk of bias for randomized controlled trials (RCTs) was assessed using the Cochrane Risk of Bias 2 tool for parallel or crossover trials;^7^ non-randomized studies of interventions were assessed using the ROBINS-I tool;^8^ prospective or retrospective cohort studies were evaluated using the Newcastle-Ottawa Scale;^9^ and cross-sectional studies by the Joanna Briggs Institute (JBI) checklist.^10^ Risk-of-bias figures were created using the Risk-of-bias VISualization (robvis) tool (University of Bristol, UK).

### Statistical Analysis

Meta-analysis was conducted using a random-effects model on RevMan 5.4 software (Cochrane, UK) and performed on data extracted from studies that were deemed to be reasonably homogenous in study population, intervention, methodology, and outcome measurement. A priori, we planned to perform meta-analysis on randomized, non-randomized interventional and observational studies separately and HD and PD studies separately. Final decisions regarding studies included in meta-analysis and comparator groups were data-driven; data from parallel-group RCTs or crossover RCTs that included patients on HD were separately pooled when deemed appropriate, whereas results from non-randomized studies and PD studies were described narratively. Dialysate bicarbonate concentration categories used for the meta-analysis comparisons were >35 mmol/L versus ≤35 mmol/L (parallel-group RCTs; outcomes of pre-dialysis serum bicarbonate, total calcium, and potassium) and ≥32 mmol/L versus ≤29 mmol/L (crossover RCTs; outcomes of pre-dialysis and post-dialysis ionized calcium and potassium). All outcomes were continuous, and the effect estimates were mean differences (MD). Where applicable, standard error was converted to standard deviation (SD) prior to meta-analysis.

### Assessment of the Certainty of the Evidence

Certainty of the evidence for each outcome included in the meta-analysis was evaluated using the Grading of Recommendations Assessment, Development, and Evaluation (GRADE) framework.^11^ RCTs started as high certainty and were rated down based on concerns in any of the five domains: risk of bias, inconsistency, indirectness, imprecision, or publication bias. Funnel plots were not used to assess publication bias because none of the comparisons had 10 or more studies. With fewer studies, funnel plot asymmetry may arise by chance,^12^ leading to potentially misleading assessments of publication bias. Therefore, no formal assessment of publication bias was conducted. The overall grading of the evidence related to each outcome was generated using GRADEPro GDT software (McMaster University and Evidence Prime, Canada).

## Results

### Patient and Study Characteristics

There were 41 studies that met inclusion criteria (n = 37 HD or hemodiafiltration [HDF]), n = 4 PD) (Supplemental Figure 1).^3,13-52^ Eligible HD or HDF studies were published from 1989 to 2024. All studies were single-center, except one international cohort study that used DOPPS data,^3^ and the number of included patients ranged from 20^36,37^ to 17,031.^3^ Mean age (SD) of included patients varied from 40 (11) to 74 (13) years. HD or HDF study designs were parallel-group RCTs (n = 7), crossover RCTs (n = 6), non-randomized interventional (n = 10), cohort (n = 11), and cross-sectional (n = 3) studies. Interventions were variable across studies (Supplemental Table 1). Eligible PD studies were published from 1997 to 2017, and sample sizes ranged from 40 to 200 patients. There were 3 randomized parallel-group RCTs^42,43,48^ and 1 non-randomized interventional study^41^ (Supplemental Table 2). The largest HD and PD parallel-group RCT included 93 and 200 patients, respectively.^17,48^

### Study Quality and Risk of Bias

**Figure 1.**
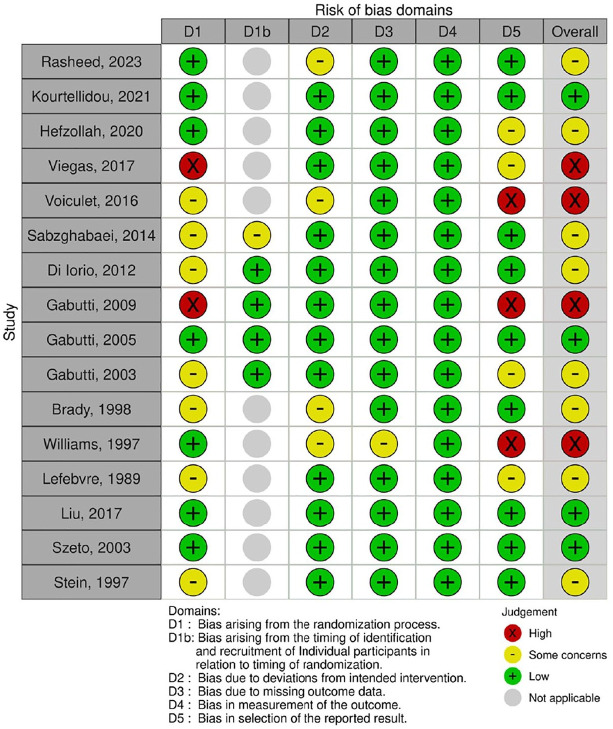
Risk of bias of randomized controlled trials.^a^ ^a^PD studies = Liu et al,^42^ Szeto et al,^43^ and Stein et al.^48^

**Figure 2.**
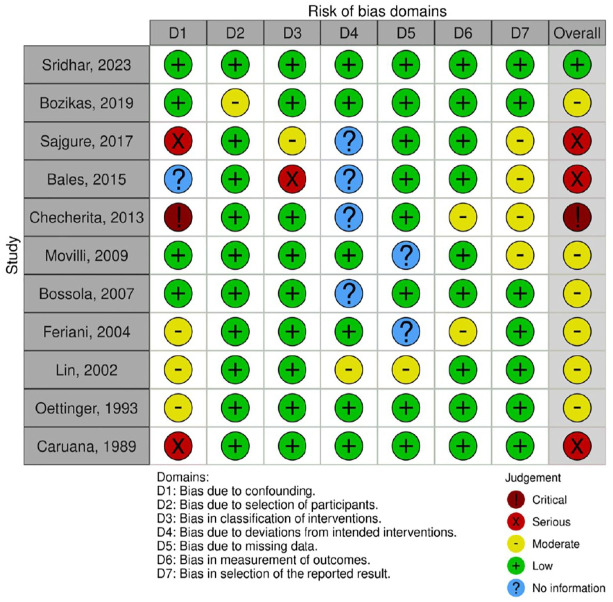
Risk of bias of non-randomized studies of interventions.^a^ ^a^PD study = Feriani et al.^41^

Most HD/HDF RCTs had some concerns (n = 7)^16,18-21,24,52^ or high risk of bias (n = 4)^17,22,25,33^ (Figure 1), as did most of the non-randomized interventional HD and HDF studies (Figure 2). The quality of HD/HDF observational studies is summarized in Supplemental Tables 3 and 4. Cohort studies varied in quality with 5 out of 11 studies rated as good quality,^3,44,45,49,51^ while all cross-sectional studies (n = 3) were of moderate quality.^14,30,39^ PD RCTs had low risk of bias^42,43^ or some concerns^48^ (Figure 1), while the non-randomized interventional study had a moderate risk of bias^41^ (Figure 2).

### Mortality and Hospitalizations

No HD/HDF RCTs examined mortality or hospitalizations. A prospective cohort study using DOPPS data found that a higher dialysate bicarbonate concentration (per 4 mmol/L higher) was associated with a higher risk of mortality (adjusted hazard ratio [HR] = 1.1, 95% confidence interval [CI] 1.0-1.2) and hospitalizations (adjusted HR = 1.1, 95% CI 1.0-1.1).^3^ A retrospective cohort study found a higher all-cause mortality risk (HR = 3.3, 95% CI 1.5-7.4) associated with a dialysate bicarbonate concentration ≥33.6 mmol/L versus 31.3-32.3 mmol/L, but no significant association with the outcome of first hospitalization (Table 1).^44^ An RCT conducted in patients treated with PD (n = 200) found the hospitalization rate and number of days spent in hospital per year to be significantly lower in the group treated with high alkali dialysate and oral sodium bicarbonate than those in the low alkali dialysate group.^48^ Another RCT of patients treated with PD (n = 60) found no difference in all-cause mortality or the number of hospitalizations with oral bicarbonate supplementation versus placebo (Table 2).^43^

**Table 1.** Mortality and Hospitalization Outcomes in HD/HDF Studies.

Outcome	Study design	Study	Exposure	Follow-up time	Number of patients	Results
All-cause mortality	Retrospective cohort	Wan et al^44^	Dialysate bicarbonate <31.3 mmol/L, 31.3-32.3 mmol/L, 32.3-33.6 mmol/L, ≥33.6 mmol/L (mean of 32.2 ± 1.6 mmol/L)	Median 65 (range, 8-71) months	313	• HR = 1.4 (95% CI 0.6-3.4), for <31.3 mmol/L compared to reference 31.3-32.3. • HR = 1.8 (95% CI 0.8-4.3), for 32.3-33.4 mmol/L compared to reference 31.3-32.3. • HR = 3.3 (95% CI 1.5-7.4) for ≥33.6 mmol/L compared to reference 31.3-32.3. • Adjusted HR = 4.3 (95% CI 2.1-8.5), per 3 mmol/L higher dialysate bicarbonate concentration.
	Prospective cohort	Tentori et al^3^	Dialysate bicarbonate (mean 35.5 ± 2.7 mmol/L)	16.4 (8.3-27.2) months	17,031	• Adjusted HR = 1.1 (95% CI 1.0-1.2), per 4 mmol/L higher dialysate bicarbonate concentration. • Dialysate bicarbonate ≥38 mmol/L, HR = 1.1 (95% CI 1.0-1.2) and ≤32 mmol/L, HR = 0.9 (95% CI 0.8-1.0), referent 33-37 mmol/L.
Cardiovascular (CV) mortality	Retrospective cohort	Wan et al^44^	Dialysate bicarbonate <31.3 mmol/L, 31.3-32.3 mmol/L, 32.3-33.6 mmol/L, ≥33.6 mmol/L	Median 65 (range, 8-71) months	313	• Incidence of CV mortality by group: <31.3 mmol/L (n = 10), 31.3-32.3 mmol/L (n = 4), 32.3-33.6 mmol/L (n = 8), ≥33.6 mmol/L (n = 13); no significant difference between groups (*P* = .1).
	Prospective cohort	Tentori et al^3^	Dialysate bicarbonate (mean 35.5 ± 2.7 mmol/L)	16.4 (8.3-27.2) months	17,031	• HR = 1.0 (95% CI 0.93-1.17), per 4 mmol/L higher dialysate bicarbonate concentration.
Infection-related mortality	Prospective cohort	Tentori et al^3^	Dialysate bicarbonate (mean 35.5 ± 2.7 mmol/L)	16.4 (8.3-27.2) months	17,031	• HR = 1.4 (95% CI 1.2-1.7), per 4 mmol/L higher dialysate bicarbonate concentration.
Hospitalization	Retrospective cohort	Wan et al^44^	Dialysate bicarbonate <31.3 mmol/L, 31.3-32.3 mmol/L, 32.3-33.6 mmol/L, ≥33.6 mmol/L	Median 65 (range, 8-71) months	313	• 215 (68.7%) first hospitalizations. Adjusted HR per 3 mmol/L higher bicarbonate concentration, 1.0; 95% CI 0.8-1.4; *P* = 0.8 (no significant association).
	Non-randomized intervention	Checheriţă et al^38^	650 mg oral bicarbonate tablets (2, 4, or 6 tablets per day) on interdialytic days	12 months	164	• Frequency of hospital admissions decreased following treatment (3.3 before treatment vs 2.9 after treatment) (no *P*-value reported, units of hospital admissions unclear).
	Prospective cohort	Tentori et al^3^	Dialysate bicarbonate (mean 35.5 ± 2.7 mmol/L)	16.4 (8.3-27.2) months	17,031	• HR = 1.1 (95% CI 1.0-1.1), per 4 mmol/L higher dialysate bicarbonate.
Arrhythmia hospitalization	Prospective cohort	Tentori et al^3^	Dialysate bicarbonate (mean 35.5 ± 2.7 mmol/L)	16.4 (8.3-27.2) months	17,031	• HR = 1.0 (95% CI 0.9-1.2), per 4 mmol/L higher dialysate bicarbonate concentration.
CV-related hospitalization	Prospective cohort	Tentori et al^3^	Dialysate bicarbonate (mean 35.5 ± 2.7 mmol/L)	16.4 (8.3-27.2) months	17,031	• HR = 1.1 (95% CI 1.0-1.2), per 4 mmol/L higher dialysate bicarbonate concentration.
Infection-related hospitalization	Prospective cohort	Tentori et al^3^	Dialysate bicarbonate (mean 35.5 ± 2.7 mmol/L)	16.4 (8.3-27.2) months	17,031	• HR = 1.0 (95% CI 0.9-1.2), per 4 mmol/L higher dialysate bicarbonate concentration.
Mean duration of hospitalization	Non-randomized intervention	Checheriţă et al^38^	650 mg oral bicarbonate tablets (2, 4, or 6 tablets per day) on interdialytic days	12 months	48	• Mean duration of hospital admission 13.6 days before treatment compared to 11.8 days after treatment.

*Note.* Conversion factor: Bicarbonate conventional unit (mEq/L) to SI unit (mmol)/L = 1. Values rounded to one decimal place or one significant figure. HR = hazard ratio.

**Table 2.** Mortality and Hospitalizations in PD Studies.

Outcome	Study design	Study	Intervention	Follow-up time	Number of patients	Results
All-cause mortality	Randomized parallel-group trial	Szeto et al^43^	Oral bicarbonate 0.9 g 3x/day for 12 months	12 months	60 (30 treatment, 30 placebo)	• 2 deaths in treatment group, 5 deaths in placebo group. • At 1 year, treatment group vs placebo group actuarial survival = 93.3% vs 83.3% (*P* = .2).
	Randomized parallel-group trial	Stein et al^48^	Low alkali (lactate 35 mmol/L) vs high alkali (lactate 40 mmol/L + optional oral sodium bicarbonate averaging 1.4 (0.2) g/day) targeting serum bicarbonate 30 mmol/L	12 months	200 (100 low alkali, 100 high alkali; 48 of high alkali receiving oral sodium bicarbonate)	• 15 deaths in the low alkali group, 12 deaths in the high alkali group (no *P*-value reported).
Cardiovascular (CV) mortality	Randomized parallel-group trial	Stein et al^48^	Low alkali (lactate 35 mmol/L) vs high alkali (lactate 40 mmol/L + optional oral sodium bicarbonate averaging 1.4 (0.2) g/day) targeting serum bicarbonate 30 mmol/L	12 months	200 (100 low alkali, 100 high alkali; 48 of high alkali receiving oral sodium bicarbonate)	• 7 deaths (6 cardiovascular disease, 1 stroke) in the low alkali group, 4 deaths (cardiovascular disease) in the high alkali group.
Number of hospitalizations	Randomized parallel-group trial	Szeto et al^43^	Oral bicarbonate 0.9g 3x/day for 12 months	12 months	60 (30 treatment, 30 placebo)	• 1.8 ± 3.1 hospitalizations per year in treatment group, and 2.4 ± 2.8 per year in placebo group (*P* = .07).
		Stein et al^48^	Low alkali (lactate 35 mmol/L) vs high alkali (lactate 40 mmol/L + optional oral sodium bicarbonate averaging 1.4 (0.2) g/day) targeting serum bicarbonate 30 mmol/L	12 months	200 (100 low alkali, 100 high alkali; 48 of high alkali receiving oral sodium bicarbonate)	• 1.1 ± 0.2 hospitalizations per patient per year in high alkali group, and 1.7 ± 0.2 in low alkali group (*P* < .05).
Hospitalization days per year	Randomized parallel-group trial	Szeto et al^43^	Oral bicarbonate 0.9g 3x/day for 12 months	12 months	60 (30 treatment, 30 placebo)	• Placebo group hospitalized for longer duration (16.8 ± 21.7 days/year) compared to treatment group (8.4 ± 17.7 days/year) (*P* = .02).
		Stein et al^48^	Low alkali (lactate 35 mmol/L) vs high alkali (lactate 40 mmol/L + optional oral sodium bicarbonate averaging 1.4 (0.2) g/day) targeting serum bicarbonate 30 mmol/L	12 months	200 (100 low alkali, 100 high alkali; 48 of high alkali receiving oral sodium bicarbonate)	• 16.4 ± 1.4 days/year in hospital in high alkali group compared to 21.2 ± 1.9 days/year in low alkali group (*P* < .05).

*Note.* Values rounded to one decimal place or one significant figure. Conversion factors: Lactate conventional unit (mg/dL) to SI unit (mmol/L) = 0.111.

### Cardiovascular Outcomes

There were 17 HD/HDF studies that reported cardiovascular outcomes (Table 3).^3,15,17,20-24,26,27,30,33,35,38,39,49,52^ No studies reported stroke or myocardial infarction. The most commonly reported outcomes were systolic (SBP) and/or diastolic blood pressure (DBP) (9 studies),^15,22-24,26,35,38,39,52^ and intradialytic hypotension (4 studies),^3,17,24,38^ and results were mixed. One crossover RCT reported significantly lower mean intradialytic SBP and DBP with a higher dialysate bicarbonate concentration (32 vs 26 mmol/L), but no significant difference in lowest intradialytic DBP.^24^ Two parallel-group RCTs found no significant differences in pre-dialysis SBP and DBP with oral bicarbonate supplementation (compared to placebo).^15,52^ Two crossover RCTs comparing higher vs lower dialysate bicarbonate concentrations found no significant differences in intradialytic SBP or DBP.^22,23^ One non-randomized interventional study examining an increase in dialysate bicarbonate depending on the pre-dialysis serum bicarbonate concentration found no difference in SBP from baseline to end of follow-up.^26^ Two other non-randomized interventional studies found no significant difference in pre-dialysis SBP or DBP with oral bicarbonate supplementation,^35,39^ whereas one study reported increased maximum mean pre-dialysis SBP and DBP following treatment with oral bicarbonate, but no impact on post-dialysis SBP or DBP.^38^

**Table 3.** Cardiovascular Outcomes in HD/HDF Studies.

Outcome	Study design	Study	Intervention	Follow-up time	Number of patients	Results
Systolic blood pressure (SBP)	Randomized parallel-group trial	Rasheed et al^52^	Oral sodium bicarbonate (500 mg) daily	3 months	50 (25 intervention, 25 control)	• Pre-HD SBP 130.3 ± 22.6 mm Hg in intervention group compared to 145.6 ± 23.9 mm Hg in control group (*P* = .3) at 3 months.
		Kourtellidou et al^15^	Average 3.7 ± 0.5 g oral sodium bicarbonate (maximum 6 g) daily	20 weeks	33 (15 intervention, 18 control)	• Pre-HD SBP 141 ± 15 mm Hg in intervention group compared to 141 ± 18 mm Hg in control group (*P* = .9) at weeks 13-16.
	Randomized crossover trial	Gabutti et al^22^	Dialysate bicarbonate 26-29 vs 32-35 mmol/L	12 weeks	21	• Intradialytic SBP maximum decrease 25.9 ± 12.9 mm Hg (dialysate 26-29 mmol/L), compared to 27.3 ± 16.1 mm Hg (dialysate 32-35 mmol/L) (*P* > .05). • Intradialytic SBP maximum increase 12.9 ± 7.1 mm Hg with dialysate 26-29 mmol/L, compared to 13.9 ± 10.3 mm Hg with dialysate 32-35 mmol/L (*P* > .05).
		Gabutti et al^23^	3 dialysate modalities: A = bicarbonate 32 mmol/L, calcium 1.3 mmol/L, B = bicarbonate 26 mmol/L, calcium 1.3 mmol/L, C = bicarbonate 32 mmol/L, calcium 1.5 mmol/L	3 weeks	27	• Lowest SBP during dialysis: modality A (115.2 ± 18.1 mm Hg) vs B (113.1 ± 19.5 mm Hg). • Highest SBP during dialysis: modality A (147.0 ± 19.1 mm Hg) vs B (146.6 ± 19.7 mm Hg). • Mean SBP during dialysis: modality A (131.8 ± 22.5 mm Hg) vs B (132.1 ± 22.8 mm Hg). • No statistically significant differences between bicarbonate 32 and 26 (modalities A and B).
		Gabutti et al^24^	Dialysate bicarbonate 32 vs 26 mmol/L	6 weeks	26	• Significant difference in mean intradialytic SBP between bicarbonate 32 (138.5 ± 23.8 mm Hg) and bicarbonate 26 (144.6 ± 24.8 mm Hg, *P* < .001). • Significant difference in lowest intradialytic SBP between bicarbonate 32 (120.8 ± 20.8 mm Hg) and bicarbonate 26 (124.3 ± 20.6 mm Hg, *P* < .01).
	Non-randomized intervention	Bales et al^26^	Dialysate bicarbonate baseline 32 mmol/L, adjusted to maintain pre-dialysis serum bicarbonate ≥22 mmol/L	4 months	48	• No significant difference in SBP from month 0 (143 ± 25 mm Hg) to month 4 (146 ± 18 mm Hg, *P* = .6) (unclear when SBP was determined relative to dialysis).
		Checheriţă et al^38^	650 mg oral bicarbonate tablets (2, 4, or 6 tablets per day) on interdialytic days	12 months	48	• Pre-HD SBP maximum mean values 162 mm Hg before therapy vs 171 mm Hg after therapy (significant, no *P*-value reported). • Post-HD SBP maximum mean values 142 mm Hg before therapy vs 144 mm Hg after therapy (insignificant, no *P*-value reported).
		Movilli et al^35^	Oral bicarbonate mean dose 2.9 ± 0.9 g/day; pre-dialysis serum bicarbonate maintained between 23-26 mmol/L	30 months	29	• Pre-HD SBP 137 ± 20 mm Hg at baseline to 139 ± 20 mm Hg (*P* > .05).
	Cross-sectional	Movilli et al^39^	Dialysate bicarbonate 35 mmol/L; Group A daily oral sodium bicarbonate administration mean dose 1.9 ± 0.9 g/day (range 1-5 g/day), Group B control	4 weeks	110 (70 Group A, 40 Group B)	• Pre-HD SBP 137 ± 20 mm Hg in oral bicarbonate group compared to 139 ± 20 mm Hg in control group, *P* > .05.
Diastolic blood pressure (DBP)	Randomized parallel-group trial	Rasheed et al^52^	Oral sodium bicarbonate (500 mg) daily	3 months	50 (25 intervention, 25 control)	• Pre-HD DBP 78.6 ± 10.7 mm Hg in intervention group compared to 85.2 ± 15.0 mm Hg in control group (*P* = .1) at 3 months.
		Kourtellidou et al^15^	Average 3.7 ± 0.5 g oral sodium bicarbonate (maximum 6 g) daily	20 weeks	33 (15 intervention, 18 control)	• Pre-HD DBP 84 ± 9 mm Hg in intervention group compared to 82 ± 11 mm Hg in control group (*P* = .7) at weeks 13-16.
	Randomized crossover trial	Gabutti et al^22^	Dialysate bicarbonate 26-29 vs 32-35 mmol/L	12 weeks	21	• Intradialytic DBP maximum decrease did not significantly differ between bicarbonate 26-29 mmol/L (12.9 ± 6.4 mm Hg) and bicarbonate 32-35 mmol/L (13.8 ± 7.2 mm Hg), *P* > .05. • Intradialytic DBP maximum increase did not significantly differ between bicarbonate 26-29 mmol/L (8.1 ± 4.2 mm Hg) and bicarbonate 32-35 mmol/L (8.7 ± 5.5 mm Hg), *P* > .05.
		Gabutti et al^23^	3 dialysate modalities: A = bicarbonate 32 mmol/L, calcium 1.3 mmol/L, B = bicarbonate 26 mmol/L, calcium 1.3 mmol/L, C = bicarbonate 32 mmol/L, calcium 1.5 mmol/L	3 weeks	27	• Lowest DBP during dialysis: modality A (58.7 ± 8.9 mm Hg) vs B (56.8 ± 9.1 mm Hg). • Highest DBP during dialysis: modality A (76.1 ± 9.0 mm Hg) vs B (74.7 ± 8.5 mm Hg). • Mean DBP: modality A (68.0 ± 11.4 mm Hg) vs B (66.9 ± 11.6 mm Hg). • No statistically significant differences between bicarbonate 32 and 26 (modalities A and B).
		Gabutti et al^24^	Dialysate bicarbonate 32 vs 26 mmol/L	6 weeks	26	• Intradialytic mean DBP significantly different with bicarbonate 32 mmol/L (71.5 ± 11.4 mm Hg) compared to bicarbonate 26 mmol/L (73.9 ± 11.5 mm Hg) (*P* < .001). • No significant difference in lowest intradialytic DBP with bicarbonate 32 mmol/L (64.1 ± 12.1 mm Hg) compared to bicarbonate 26 mmol/L (65.0 ± 12.1 mm Hg), *P* > .05.
	Non-randomized intervention	Checheriţă et al^38^	650 mg oral bicarbonate tablets (2, 4, or 6 tablets per day) on interdialytic days	12 months	48	• Minimum mean pre-HD DBP 86 mm Hg before therapy vs 92 mm Hg after therapy (significant, no *P*-value reported). • Minimum mean post-dialysis DBP 76 mm Hg before therapy vs 80 mm Hg after therapy (insignificant, no *P*-value reported).
		Movilli et al^35^	Oral bicarbonate mean dose 2.9 ± 0.9 g/day; pre-dialysis serum bicarbonate maintained between 23-26 mmol/L	30 months	29	• Pre-HD DBP 75 ± 11 mm Hg at baseline to 77 ± 11 mm Hg (*P* > .05).
	Cross-sectional	Movilli et al^39^	Dialysate bicarbonate 35 mmol/L; Group A daily oral sodium bicarbonate administration mean dose 1.9 ± 0.9 g/day (range 1-5 g/day), Group B control	4 weeks	110 (70 Group A, 40 Group B)	• Pre-HD DBP 75 ± 11 mm Hg with oral bicarbonate vs 77 ± 11 mm Hg in control (*P* > .05).
Mean arterial pressure	Non-randomized intervention	Lin et al^27^	Dialysate bicarbonate increased from 35 to 38 mmol/L (month 1), then adjusted to 38-40 mmol/L in subgroup A (patients with pre-dialysis serum bicarbonate ≤21 mmol/L).	6 months	17	• Baseline = 97.6 ± 3.2 mm Hg, 3 months = 96.6 ± 4.0 mm Hg, 6 months = 94.6 ± 3.8 mm Hg (*P* > .05).
Intradialytic hypotension	Randomized parallel-group trial	Viegas et al^17^	Dialysate bicarbonate 34 vs 30 mmol/L	9 months	93 (46: 34 mmol/L, 47: 30 mmol/L)	• No significant difference in rate of intradialytic hypotension: 28.0 events per 1000 sessions in bicarbonate 34 mmol/L, compared to 27.4 per 1000 sessions in bicarbonate 30 mmol/L (*P* = .9).
	Randomized crossover trial	Gabutti et al^24^	Dialysate bicarbonate 32 vs 26 mmol/L	6 weeks	26	• Percentage of dialysis sessions with hypotensive episodes was 5.5% in bicarbonate 32 mmol/L, compared to 1.7% in bicarbonate 26 mmol/L (*P* < .05).
	Non-randomized intervention	Checheriţă et al^38^	650 mg oral bicarbonate tablets (2, 4, or 6 tablets per day) on interdialytic days	12 months	48	• Before therapy = 32 (67%) patients, after therapy = 19 (39%) patients.
	Prospective cohort	Tentori et al^3^	Dialysate bicarbonate (mean 35.5 ± 2.7 mmol/L)	16.4 (8.3-27.2) months	17,031	• Positive association between dialysate bicarbonate and the incidence of intradialytic hypotension (HR per 4 mmol/L higher bicarbonate concentration, 1.1, 95% CI 1.0-1.3).
Vascular calcification	Randomized parallel-group trial	Voiculeț et al^33^	Oral bicarbonate 5 g/day on non-dialysis days in intervention group; mean (SD) dialysate bicarbonate 29.8 (1.4) mmol/L intervention, 33 (2.2) mmol/L control	12 months	63 (29 intervention, 34 control)	• 51.9% of intervention group by end of study vs 75.0%, 58.3%, and 92.9% in control subgroups 1 (pre-dialysis alkaline reserve 22 to 24 mmol/L), 2 (pre-dialysis alkaline reserve 20 to 22 mmol/L), and 3 (pre-dialysis alkaline reserve <20 mmol/L), respectively (*P* = .03).
Stroke volume	Randomized crossover trial	Gabutti et al^22^	Dialysate bicarbonate 26-29 vs 32-35 mmol/L	12 weeks	21	• Intradialytic stroke volume maximum decrease did not significantly differ between bicarbonate 26-29 mmol/L (18.6 ± 11.8 mL) and bicarbonate 32-35 mmol/L (20.6 ± 13.6 mL), *P* > .05. • Intradialytic stroke volume maximum increase did not significantly differ between bicarbonate 26-29 mmo/L (24.6 ± 8.8 mL) and bicarbonate 32-35 mmol/L (27.3 ± 12.1 mL), *P* > .05.
Peripheral resistance	Randomized crossover trial	Gabutti et al^22^	Dialysate bicarbonate 26-29 vs 32-35 mmol/L	12 weeks	21	• Intradialytic peripheral resistance maximum decrease did not significantly differ between bicarbonate 26-29 mmol/L (345 ± 288 dyne s cm^-5^) and bicarbonate 32-35 mmol/L (286 ± 198 dyne s cm^-5^), *P* > .05. • Intradialytic peripheral resistance maximum increase did not significantly differ between bicarbonate 26-29 mmol/L (520 ± 200 dyne s cm^-5^) and bicarbonate 32-35 mmol/L (690 ± 455 dyne s cm^-5^), *P* > .05.
PR interval	Randomized parallel-group trial	Kourtellidou et al^15^	Average 3.7 ± 0.5 g oral sodium bicarbonate (maximum 6 g) daily	20 weeks	33 (15 intervention, 18 control)	• Effect of HD on PR interval −11.5 ± 21.0 in intervention group compared to −9.9 ± 11.5 in control group (*P* = .7) at weeks 13-16.
QTc interval	Randomized parallel-group trial	Kourtellidou et al^15^	Average 3.7 ± 0.5 g oral sodium bicarbonate (maximum 6 g) daily	20 weeks	33 (15 intervention, 18 control)	• Effect of HD on corrected QT +11.7 ± 28.8 in intervention group compared to +14.9 ± 20.8 in control group (*P* = .7) at weeks 13-16. • Effect of HD on QT dispersion −7.3 ± 13.6 in intervention group compared to −0.4 ± 16.2 in control group (*P* = .2) at weeks 13-16.
	Randomized crossover trial	Sabzghabaei et al^20^	4 dialysate solutions: #1 bicarbonate 24 mmol/L, potassium 2 mmol/L, sodium 140 mmol/L, calcium 2.5 mmol/L; #2 bicarbonate 28 mmol/L, potassium 2 mmol/L, sodium 140 mmol/L, calcium 2.5 mmol/L; #3 bicarbonate 24 mmol/L, potassium 3 mmol/L, sodium 140 mmol/L, calcium 2.5 mmol/L; #4 bicarbonate 28 mmol/L, potassium 3 mmol/L, sodium 140 mmol/L, calcium 2.5 mmol/L	Dialysis session	35	• No significant difference in mean QTc before vs after dialysis in any of the solutions: • Solution 1: 434.5 ± 29.6 vs 435.0 ± 43.8 ms (*P* = .9) • Solution 2: 428.7 ± 45.7 vs 436.9 ± 32.5 ms (*P* = .2) • Solution 3: 440.5 ± 45.7 vs 447.0 ± 34.0 ms (*P* = .2) • Solution 4: 437.3 ± 43.8 vs 436.0 ± 83.8 ms (*P* = .8)
		Di Iorio et al^21^	Dialysate bicarbonate 30 vs 34 mmol/L	Dialysis session	22	• Mean QTc change during dialysis 40 ± 10 ms with dialysate bicarbonate 34 mmol/L, 2 ± 2 ms with dialysate bicarbonate 30 mmol/L (*P* < .01). • Higher dialysate bicarbonate predicted increased QTc (HR 1.5, 95% CI 1.3-1.7, *P* = .001).
Cardiac index	Cross-sectional	Silva et al^30^	Dialysate bicarbonate (mean 38.2 ± 2.1 mmol/L)	Dialysis session	30	• Higher dialysate bicarbonate correlated with higher cardiac index drop (r = −0.4, *P* = .001). • Patients dialyzed with higher bicarbonate (>38 mmol/L) had higher post-HD cardiac index drop (−0.9 ± 0.6 vs −0.1 ± 0.5 l/min/m^2^, *P* = .002).
Arrhythmia	Prospective cohort	Ravi et al^49^	Dialysate bicarbonate <35, 35, and >35 mmol/L (fixed)	12 months	66	• Lower incidence of arrhythmia episodes associated with dialysate bicarbonate >35 than 35 mmol/L (incidence rate ratio [IRR] 0.45 (0.27, 0.75) and aIRR 0.54 (0.30, 0.97)); association not significant when adjusting for serum and dialysate potassium levels (aIRR 0.60 (0.32, 1.11)).

*Note.* CI = confidence interval, HD = hemodialysis, HR = hazard ratio, SBP = systolic blood pressure, DBP = diastolic blood pressure, IRR = incidence rate ration, aIRR = adjusted incidence rate ratio. Conversion factors: Bicarbonate conventional unit (mEq/L) to SI unit (mmol/L) = 1, Calcium conventional unit (mg/dL) to SI unit (mEq/L) = 0.25, potassium conventional unit (mEq/L) to SI unit (mmol)/L = 1, sodium conventional unit (mEq/L) to SI unit (mmol)/L = 1. Values rounded to one decimal place or one significant figure.

There was a positive association between dialysate bicarbonate concentration and the incidence of intradialytic hypotension in 2 of 3 studies comparing higher vs lower dialysate bicarbonate,^3,24^ while the incidence of intradialytic hypotension was found to be lower following oral bicarbonate supplementation in one pre-post intervention study.^38^ The largest HD parallel-group RCT found no significant difference in the rate of intradialytic hypotension between dialysate bicarbonate groups of 34 vs 30 mmol/L,^17^ whereas the largest observational study found a positive association between dialysate bicarbonate concentration and the incidence of intradialytic hypotension (HR per 4 mmol/L higher bicarbonate concentration, 1.1, 95% CI 1.0-1.3).^3^

QTc interval was reported in two crossover RCTs; one trial comparing dialysate bicarbonate concentrations of 24 vs 28 mmol/L found no difference between groups in QTc interval pre-dialysis or post-dialysis,^20^ and one trial found a significantly larger increase in QTc during dialysis with higher vs lower dialysate bicarbonate concentration (40 ms ± 10 vs 2 ms ± 2 with dialysate bicarbonate 34 vs 30 mmol/L respectively).^21^

An RCT in 200 patients treated with PD found no between-group difference in mean SBP or DBP (130 ± 2/78 ± 1 mm Hg vs 129 ± 3/76 ± 1 mm Hg, high vs low alkali) at 1 year.^48^ The other two PD RCTs also did not find any differences in SBP or DBP (Supplemental Table 5).^42,43^

### Nutritional Outcomes

There were 18 HD/HDF studies that reported nutritional outcomes (Supplemental Table 6).^14-16,18,25-28,32,34-37,39,45,50-52^ RCTs (n = 3) comparing different dialysate bicarbonate concentrations found no difference in serum albumin.^16,18,25^ An RCT comparing daily oral sodium bicarbonate supplementation to no supplementation found higher albumin in the intervention group than the control group at 3 months (4.2 ± 0.5 g/L vs 3.8 ± 0.5 g/L, *P* = .01).^52^ Most non-randomized interventional studies (n = 4 of 6) found no difference in serum albumin with higher dialysate bicarbonate concentration or with oral bicarbonate supplementation.^27,35-37^ Most HD/HDF studies that examined normalized protein catabolic rate (nPCR) (n = 6 of 8) found no difference with higher, compared with lower, dialysate bicarbonate concentrations^18,25,27^ or with daily oral bicarbonate supplementation.^15,36,39^ No HD/HDF studies found oral or dialysate bicarbonate interventions to have an impact on Subjective Global Assessment (SGA) (n = 2).^32,36^

Three PD studies reported nutritional outcomes (serum albumin, lean body mass, protein nitrogen appearance [PNA], and SGA).^41,43,48^ A parallel-group RCT reported significant increases in normalized PNA and SGA following 12-months of oral bicarbonate supplementation (compared to placebo), but no differences in lean body mass or serum albumin between-groups after adjusting for the Charlson Comorbidity Index.^43^ Another RCT found no within-group differences in serum albumin at 1 year or PCR at 1 month in patients treated with high alkali dialysate and oral bicarbonate supplementation vs low alkali dialysate.^48^ A non-randomized interventional study showed no difference in PNA with an increase in dialysate bicarbonate concentration from 34 to 39 mmol/L (Supplemental Table 7).^41^

### Serum Bicarbonate, pH, Calcium, Potassium, and PTH

Results for laboratory measurement outcomes are summarized in Table 4 for HD/HDF studies and Supplemental Table 8 for PD studies. There were 22 of 27 HD/HDF studies that showed significantly higher pre-dialysis serum bicarbonate with higher dialysate bicarbonate concentration^3,13,17-19,27,28,31,32,44,45,51^ or oral bicarbonate supplementation.^15,33-40,52^ A significantly higher post-dialysis serum bicarbonate concentration was observed with higher dialysate bicarbonate concentration in 11 of 15 HD/HDF studies,^13,14,17,19,21,24,28,31,40,44,45^ There were 9 of 16 HD/HDF studies that found higher pre-dialysis pH with higher dialysate bicarbonate concentration or oral bicarbonate supplementation.^19,22,25,27,31,34,35,37,39^ Post-dialysis pH was found to be significantly higher with higher dialysate bicarbonate concentration in 5 of 9 HD/HDF studies.^21-24,46^

**Table 4.** Bicarbonate, pH, Calcium, Potassium, and PTH in HD/HDF Studies.

Outcome	Study design	Study	Intervention	Follow-up time	Number of patients	Results
Pre-dialysis serum bicarbonate	Randomized parallel-group trial	Rasheed et al^52^	Oral sodium bicarbonate (500 mg) daily	3 months	50 (25 intervention, 25 control)	• 20.7 ± 2.5 mmol/L in intervention group vs 18.9 ± 3.9 mmol/L in control group (*P* < .05) at 3 months.
		Kourtellidou et al^15^	Average 3.7 ± 0.5 g oral sodium bicarbonate (maximum 6 g) daily	20 weeks	36 (16 intervention, 20 control)	• 20.1 ± 1.9 mmol/L in intervention group vs 16.8 ± 1.8 mmol/L in control group at weeks 13-16 (*P* < .001).
		Hefzollah et al^16^	Dialysate bicarbonate 36 vs 30 mmol/L	6 months	56 (26: 36 mmol/L, 30: 30 mmol/L)	• 20.1 ± 2.5 mmol/L at end of study compared to 20.3 ± 4.3 mmol/L at beginning of study, *P* = .8 (dialysate bicarbonate 30 mmol/L). • 20.4 ± 2.3 mmol/L at end of study compared to 20.0 ± 3.3 mmol/L at beginning of study, *P* = .7 (dialysate bicarbonate 36 mmol/L).
		Viegas et al^17^	Dialysate bicarbonate 34 vs 30 mmol/L	9 months	93 (46 34 mmol/L, 47 30 mmol/L)	• 22.7, 95% CI 20.9-24.4 mmol/L with dialysate bicarbonate 34 mmol/L vs 21.1, 95% CI 19.7-22.7 mmol/L with dialysate bicarbonate 30 mmol/L) (*P* < .001).
		Voiculeț et al^33^	Oral bicarbonate 5 g/day on non-dialysis days in intervention group; mean (SD) dialysate bicarbonate 29.8 (1.4) mmol/L intervention, 33 (2.2) mmol/L control	12 months	63 (29 intervention, 34 control)	• Bicarbonate concentration of 20-22 mmol/L in 93.1% of intervention group and 35.3% of control (*P* < .001).
		Brady and Hasbargen^18^	40 mmol/L dialysate bicarbonate, plus 1 mmol/kg/d oral sodium bicarbonate if pre-dialysis serum bicarbonate <22 mmol/L after 2 weeks vs 35 mmol/L dialysate bicarbonate	16 weeks	36 (18: 40 mmol/L, 18: 35 mmol/L)	• 17.3 ± 3.2 mmol/L (35 mmol/L) vs 20.2 ± 2.9 mmol/L (40 mmol/L) (*P* < .01) at the end of study.
		Lefebvre et al^19^	Dialysate bicarbonate 33 ± 2 mmol/L control, intervention 7-15 mmol/L added targeting pre-dialysis serum bicarbonate 24 mmol/L	18 months	21 (11 intervention, 10 control)	• 24.0 ± 0.6 mmol/L at follow-up vs baseline 15.6 ± 1.1 mmol/L (*P* < .05) in intervention group; 16.5 ± 0.9 mmol/L at follow-up vs baseline 16.8 ± 0.9 mmol/L (*P* > .05) in control group^a^
	Randomized crossover trial	Gabutti et al^24^	Dialysate bicarbonate 32 vs 26 mmol/L	6 weeks	26	• No significant difference between dialysate bicarbonate 32 mmol/L (21.1 ± 2.9 mmol/L) and dialysate bicarbonate 26 mmol/L (20.2 ± 2.8 mmol/L), *P* > .05.
	Non-randomized intervention	Bozikas et al^40^	Dialysate/oral bicarbonate 37 mmol/L (period A) vs 35 mmol/L + 5g/day (÷ 3 doses) (period B)	4 weeks	25	• 21.5 ± 2.5 mmol/L after treatment with dialysate bicarbonate 37 mmol/L vs 23.0 ± 2.8 mmol/L after dialysate bicarbonate 35 mmol/L and 5 g oral sodium bicarbonate (*P* = .03).
		Sajgure et al^34^	Oral bicarbonate mean dose 0.7 ± 0.4 mmol/kg/day at baseline to 1.0 ± 0.6 mmol/kg/day; dosage increased if baseline serum bicarbonate <22 mmol/L	3 months	35	• 24.6 ± 3.5 mmol/L at 3 months vs baseline 20.2 ± 4.9 mmol/L (*P* < .001). • Moderate positive correlation between change in oral bicarbonate supplementation and serum bicarbonate concentration (r = 0.56, *P* < .001).
		Bales et al^26^	Dialysate bicarbonate baseline 32 mmol/L, adjusted to maintain pre-dialysis serum bicarbonate ≥22 mmol/L	4 months	48	• Change in serum bicarbonate from baseline to month 4 was 2.0 (0.5, 3.2) mmol/L in the PTH <31.6 pmol/L group, and 3.4 (1.1, 4.0) mmol/L in the PTH >31.6 pmol/L group (*P* = .2 comparing the PTH groups).
		Checheriţă et al^38^	650 mg oral bicarbonate tablets (2, 4, or 6 tablets per day) on interdialytic days	12 months	48	• Serum bicarbonate significantly increased from 19.4 mmol/L at baseline to 22.6 ± 1.3 mmol/L at 12 months (*P* < .001).
		Movilli et al^35^	Oral bicarbonate mean dose 2.9 ± 0.9 g/day; pre-dialysis serum bicarbonate maintained between 23-26 mmol/L	30 months	29	• Serum bicarbonate 24.6 ± 1.1 mmol/L, vs baseline 19.1 ± 0.7 mmol/L (*P* < .001).
		Bossola et al^36^	Oral bicarbonate 1 g 3x/day	12 months	20	• Serum bicarbonate 22.1 ± 4.5 mmol/L at 12 months vs 18.1 ± 2.7 mmol/L at baseline, *P* = .001.
		Lin et al^27^	Dialysate bicarbonate increased from 35 to 38 mmol/L (month 1), then adjusted to 38-40 mmol/L in subgroup A (patients with pre-dialysis serum bicarbonate ≤21 mmol/L)	6 months	21	• Baseline serum bicarbonate 18.4 ± 0.3 mmol/L vs 24.2 ± 0.2 mmol/L at 6 months.
		Oettinger and Oliver^13^	Dialysate bicarbonate switched from 39 to 42 mmol/L (0-12 weeks) to 39 mmol/L (12-20 weeks)	20 weeks	38	• 24.7 mmol/L, error = 0.5 at week 12.^b^ • 19.1 mmol/L, error = 0.4 at week 20 (*P* < .05 weeks 12-20).^b^
		Caruana et al^37^	Oral bicarbonate targeting mid-week pre-dialysis bicarbonate 22-26 mmol/L	1 month	20	• Serum bicarbonate 22.5 ± 4.0 mmol/L vs baseline 18.6 ± 2.9 mmol/L, *P* < .001.
	Prospective cohort	Law and Davenport^45^	Dialysate bicarbonate 32 mmol/L at baseline then changed to 28 mmol/L	19 months	126	• 21.1 ± 2.3 mmol/L at baseline (dialysate bicarbonate 32 mmol/L) and 19.4 ± 2.7 mmol/L at study end (dialysate 28 mmol/L), *P* < .001.
		Cuadrado et al^47^	Dialysate bicarbonate individually modified according to pre-dialysis and post-dialysis serum bicarbonate targets of 19-25 mmol/L and 27-29 mmol/L (100% on 32 mmol/L dialysate bicarbonate at study start and 75% on 32-34 mmol/L dialysate bicarbonate at study end)	6 months	123	• 23.3 ± 2.2 mmol/L at baseline to 21.3 ± 1.5 (2 months), 21.6 ± 1.5 (4 months), 22.9 ± 1.5 mmol/L (6 months).
		Montagud-Marrahi et al^28^	Dialysate bicarbonate 35 mmol/L at baseline switched to 32 mmol/L at study start	6 months	84	• Pre-HD bicarbonate 26.8 ± 1.3 mmol/L at baseline vs 23.7 ± 1.9 mmol/L at 6 months (*P* < .001).
		Valério Alves et al^50^	Dialysate bicarbonate adjusted every 3 months (9 time points): dialysate bicarbonate >30 mmol/L. reduce 4 mmol/L; ≥25 mmol/L, reduce 2 mmol/L; 20 mmol/L – 25 mmol/L, no change; ≤20 mmol/L, increase 2 mmol/L; <18 mmol/L, increase 4 mmol/L (individualized)	24 months	31	• 25.9 ± 1.8 mmol/L at baseline (mean dialysate bicarbonate 32 mmol/L) to 23.4 ± 2.0 mmol/L at 24 months (mean dialysate bicarbonate 28 mmol/L).
		Tentori et al^3^	Dialysate bicarbonate (mean 35.5 ± 2.7 mmol/L)	16.4 (8.3-27.2) months	17,031	• 22.2 ± 3.3 mmol/L with ≤32 mmol/L, 23.0 ± 3.4 mmol/L with 33-37 mmol/L, 23.1 ± 3.9 mmol/L with ≥ 38 mmol/L. • Pre-dialysis serum bicarbonate not strongly correlated with dialysate bicarbonate (Spearman correlation, 0.09).
		Noh et al^31^	Dialysate bicarbonate 25 vs 30 vs 35 mmol/L	64.4 ± 47 months	53	• Pre-HD 21.6 ± 2.6 mmol/L (25 mmol/L), 20.9 ± 2.8 mmol/L (30 mmol/L), and 22.9 ± 3.4 mmol/L (35 mmol/L).
		Blair et al^32^	Dialysate bicarbonate baseline 35 mmol/L increased to 39 mmol/L	6 months	199	• 21.7 ± 2.8 mmol/L at baseline vs 23.1 ± 3.3 mmol/L at 6 months (*P* > .001).
	Retrospective cohort	Molnar et al^51^	Standardized dialysate bicarbonate (35, 36-37, and ≥38 mmol/L) and individualized dialysate bicarbonate concentration	12 months	5414	• 25.0 (2.6), 24.8 (2.5), 24.1 (2.3), 25.7 (2.0), and 26.0 (2.7) mmol/L in individualized group, combined standardized group all together, 35 mmol/L group, 36-37 mmol/L group, and ≥38 mmol/L group, respectively. • Regression coefficients for association of dialysate bicarbonate with outpatient serum bicarbonate (mmol/L): • Individualized vs standardized = −0.3 (95% CI −1.5 to 0.8) unadjusted, −0.3 (−0.9, 0.4) adjusted • Individualized vs 35 mmol/L = −0.8 (−2.0, 0.4) unadjusted, −0.6 (−1.4, 0.2) adjusted • Individualized vs 36-37 mmol/L = 0.2 (−2.0, 2.5) unadjusted, 0.1 (−1.4, 1.6) adjusted • Individualized vs ≥38 mmol/L = 0.7 (−1.1, 2.4) unadjusted, 0.3 (−0.7, 1.3) adjusted • 35 vs 36-37 mmol/L = 1.1 (−0.8, 2.9) unadjusted, 0.7 ( −0.3, 1.7) adjusted • 35 vs ≥38 mmol/L = 1.5 (0.1, 2.8) unadjusted, 0.9 (0.1, 1.6) adjusted
		Wan et al^44^	Dialysate bicarbonate <31.3 mmol/L, 31.3-32.3 mmol/L, 32.3-33.6 mmol/L, ≥33.6 mmol/L	Median 65 (range, 8-71) months	313	• Dialysate bicarbonate positively associated with pre-HD serum bicarbonate (r = 0.2, *P* = .002).
	Cross-sectional	Movilli et al^39^	Dialysate bicarbonate 35 mmol/L; Group A daily oral sodium bicarbonate administration mean dose 1.9 ± 0.9 g/day (range 1-5 g/day), Group B control	4 weeks	110 (70 Group A, 40 Group B)	• 23.8 ± 1.4 mmol/L (A) vs 20.9 ± 1.4 mmol/L (B), *P* = .001.
Post-dialysis serum bicarbonate	Randomized parallel-group trial	Kourtellidou et al^15^	Average 3.7 ± 0.5 g oral sodium bicarbonate (maximum 6 g) daily	16 weeks (4 week run in, 12 weeks of treatment)	36 (16 intervention, 20 control)	• Pre-HD total calcium 24.2 ± 1.0 mmol/L in intervention group vs 23.3 ±1.6 mmol/L in control group at weeks 13-16 (*P* = 0.08).
		Viegas et al^17^	Dialysate bicarbonate 34 vs 30 mmol/L	9 months	93 (46: 34 mmol/L, 47: 30 mmol/L)	• 28.0 (IQR 26.9 to 29.1) mmol/L with bicarbonate 34 mmol/L vs 25.3 (IQR 24.0-26.5) mmol/L with bicarbonate 30 mmol/L (*P* < .001).
		Lefebvre et al^19^	Dialysate bicarbonate 33 ± 2 mmol/L control, intervention 7-15 mmol/L added targeting pre-dialysis serum bicarbonate 24 mmol/L	18 months	21 (11 intervention, 10 control)	• 29.0 ± 0.6 mmol/L at end of study compared to 21.0 ± 0.9 mmol/L at beginning of study, *P* < .05 (intervention group). • 19.4 ± 0.9 mmol/L at end of study compared to 20.8 ± 1.1 mmol/L at beginning of study, *P* > .05 (control group).^a^
	Randomized crossover trial	Di Iorio et al^21^	Dialysate bicarbonate 30 vs 34 mmol/L	4 hours	22	• 29 ± 0.7 mmol/L (Bic 30, Ca 1.3 mmol/L) vs 33 ± 1.0 mmol/L (Bic 34, Ca 1.3 mmol/L).
		Gabutti et al^24^	Dialysate bicarbonate 32 vs 26 mmol/L	6 weeks	26	• 26.6 ± 3.1 mmol/L with dialysate bicarbonate 32 mmol/L vs 23.7 ± 2.2 mmol/L with dialysate bicarbonate 26 mmol/L (*P* < .05).
	Non-randomized intervention	Bozikas et al^40^	Dialysate/oral bicarbonate 37 mmol/L vs 35 mmol/L + 5 g/day (÷ 3 doses)	4 weeks	25	• 27.6 ± 1.7 mmol/L after treatment with dialysate bicarbonate 37 vs 26.4 ± 1 mmol/L (*P* = .03) after dialysate bicarbonate 35 mmol/L and 5 g oral sodium bicarbonate.
		Oettinger and Oliver^13^	Dialysate bicarbonate switched from 39 to 42 mmol/L (0-12 weeks) to 39 mmol/L (12-20 weeks)	20 weeks	38	• Week 0 post-dialysis serum bicarbonate = 23.2 mmol/L, error = 0.5^b^ • Week 12 post-dialysis serum bicarbonate = 28.9 mmol/L, error = 0.5 (*P* < .0001 weeks 1-12).^b^
	Prospective cohort	Law and Davenport^45^	Dialysate bicarbonate 28 mmol/L versus 32 mmol/L	19 months	126	• 24.9 ± 2.1 at baseline (dialysate bicarbonate 32 mmol/L) and 23.1 ± 1.8 mmol/L at study end (dialysate 28 mmol/L), *P* < .001.
		Cuadrado et al^47^	Dialysate bicarbonate individually modified according to pre-dialysis and post-dialysis serum bicarbonate targets of 19-25 mmol/L and 27-29 mmol/L (75% on 32-34 mmol/L dialysate bicarbonate at end of study)	6 months	123	• 27.4 ± 1.8 mmol/L at baseline to 25.4 ± 1.1 (2 months), 25.5 ± 1.1 (4 months), 26.8 ± 1.3 mmol/L (6 months).
		Montagud-Marrahi et al^28^	Dialysate bicarbonate 35 mmol/L at baseline switched to 32 mmol/L at study start	6 months	84	• Post-HD bicarbonate 31.9 ± 0.9 mmol/L at baseline vs 27.6 ± 1.4 mmol/L at 6 months (*P* < .001).
		Panesar et al^29^	Dialysate bicarbonate 30-32, 33-34, 35-36, or 37-40 mmol/L	Not stated	39	• No statistically significant difference in serum bicarbonate 68 hours post-HD across all groups: 21.1 ± 3.4 mmol/L (bicarbonate 30-32), 20.4 ± 2.5 mmol/L (bicarbonate 33-34), 21.1 ± 2.4 mmol/L (bicarbonate 35-36), 22.2 ± 2.6 mmol/L (bicarbonate 37-40).
		Noh et al^31^	Dialysate bicarbonate 25 vs 30 vs 35 mmol/L	64.4 ± 47 months	53	• Post-HD 27.4 ± 1.5 mmol/L in 25 mmol/L group, 28.5 ± 3.4 mmol/L in 30 mmol/L group, and 31.2 ± 1.4 mmol/L in 35 mmol/L group (*P* < .05 between bicarbonate 35 vs bicarbonate 30 vs bicarbonate 25).
	Retrospective cohort	Wan et al^44^	Dialysate bicarbonate <31.3 mmol/L, 31.3-32.3 mmol/L, 32.3-33.6 mmol/L, ≥33.6 mmol/L	Median 65 (range, 8-71) months	313	• Dialysate bicarbonate positively associated with post-HD serum bicarbonate (r = 0.2, *P* < .01).
	Cross-sectional	Wieliczko and Małyszko^14^	Dialysate bicarbonate mean 32.9 ± 1.8 mmol/L (range 28-36 mmol/L), titrated depending on pre-dialysis serum bicarbonate	Dialysis session	75	• Non-significant correlation between bicarbonate bath and pre-HD serum bicarbonate. • Correlation between bicarbonate bath and post-HD serum bicarbonate = 0.5, *P* < .001.
		Silva et al^30^	Dialysate bicarbonate (mean 38.2 ± 2.1 mmol/L)	Dialysis session	30	• 21.3 (18.8, 22) mmol/L with ≤38 mmol/L dialysate bicarbonate vs 20.4 (18.9, 22.4) mmol/L with >38 mmol/L dialysate bicarbonate (*P* = 1).^c^
Pre-dialysis pH	Randomized parallel-group trial	Lefebvre et al^19^	Dialysate bicarbonate 33 ± 2 mmol/L control, intervention 7-15 mmol/L added targeting pre-dialysis serum bicarbonate 24 mmol/L	18 months	21 (11 intervention, 10 control)	• 7.4 ± 0.02 at baseline to 7.4 ± 0.01 in intervention group (*P* < .05 from baseline). • 7.4 ± 0.02 at baseline to 7.4 ± 0.02 in control group (*P* > .05).
	Randomized crossover trial	Gabutti et al^22^	Dialysate bicarbonate 26-29 vs 32-35 mmol/L	12 weeks	21	• 7.4 ± 0.05 (26-29 mmol/L), 7.4 ± 0.09 (32-25 mmol/L), *P* = .05.
		Gabutti et al^23^	3 dialysate modalities: A = bicarbonate 32 mmol/L, calcium 1.3 mmol/L, B = bicarbonate 26 mmol/L, calcium 1.3 mmol/L, C = bicarbonate 32 mmol/L, calcium 1.5 mmol/L	3 weeks	27	• 7.4 ± 0.04 (A), 7.4 ± 0.05 (B), 7.4 ± 0.04 (C).
		Gabutti et al^24^	Dialysate bicarbonate 32 vs 26 mmol/L	6 weeks	26	• 7.4 ± 0.06 (32 mmol/L) vs 7.4 ± 0.06 (26 mmol/L), *P* > .05.
		Williams et al^25^	Group A (dialysate bicarbonate 30 to 40 mmol/L), Group B (40 mmol/L to 30 mmol/L), and standard bicarbonate 35 mmol/L	12 months (crossover at 6 months)	46	• Mean pre-HD pH was 7.4 ± 0.05 while using 30 mmol/L versus 7.4 ± 0.04 with 40 mmol/L, *P* < .001.
	Non-randomized intervention	Sridhar et al^46^	4 dialysate solutions with either 38 or 32 mmol/L bicarbonate	Dialysis solution for 3 consecutive alternating day treatments over 1 week, outcomes measured after 1-week period	25	• Linear regression coefficient for dialysate bicarbonate 38 vs 32 mmol/L= 0.01, *P* = .05.
		Bozikas et al^40^	Dialysate/oral bicarbonate 37 mmol/L (period A) vs 35 mmol/L + 5g/day (÷ 3 doses) (period B)	4 weeks	25	• Pre-HD pH = 7.3 ± 0.05 at end of period A. • Pre-HD pH = 7.4 ± 0.05 at end of period B.
		Sajgure et al^34^	Oral bicarbonate mean dose 0.7 ± 0.4 mmol/kg/day at baseline to 1.0 ± 0.6 mmol/kg/day; dosage increased if baseline serum bicarbonate <22 mmol/L	3 months	35	• 7.3 ± 0.08 at baseline to 7.4 ± 0.09 at 3 months (*P* < .001).
		Bales et al^26^	Dialysate bicarbonate baseline 32 mmol/L, adjusted to maintain pre-dialysis serum bicarbonate ≥22 mmol/L	4 months	48	• Change in pH from 0 to 4 months in PTH <31.6 pmol/L group = 0.02 ± 0.04. • Change in pH from 0-4 months in PTH >31.6 pmol/L group = 0.03 ± 0.03 (*P* = .2 versus PTH <31.6 pmol/L group).
		Movilli et al^35^	Oral bicarbonate mean dose 2.9 ± 0.9 g/day; pre-dialysis serum bicarbonate maintained between 23-26 mmol/L	30 months	29	• 7.3 ± 0.03 at baseline to 7.4 ± 0.02, *P* < .0001.
		Lin et al^27^	Dialysate bicarbonate increased from 35 to 38 mmol/L (month 1), then adjusted to 38-40 mmol/L in subgroup A (patients with pre-dialysis serum bicarbonate ≤21 mmol/L)	6 months	17	• Baseline 7.3 ± 0.01 to 7.4 ± 0.01 at 6 months, *P* < .001.
		Caruana et al^37^	Oral bicarbonate targeting mid-week pre-dialysis bicarbonate 22-26 mmol/L	1 month	20	• 7.4 ± 0.03 pre-therapy to 7.4 ± 0.04 post-therapy, *P* < .05.
		Noh et al^31^	Dialysate bicarbonate 25 vs 30 vs 35 mmol/L	64.4 ± 47 months	53	• 25 mmol/L group = 7.4 ± 0.02, 30 mmol/L group = 7.4 ± 0.05, 35 mmol/L group = 7.4 ± 0.04.
	Prospective cohort	Valério Alves et al^50^	Dialysate bicarbonate adjusted every 3 months (9 time points): dialysate bicarbonate >30 mmol/L. reduce 4 mmol/L; ≥25 mmol/L, reduce 2 mmol/L; 20 mmol/L – 25 mmol/L, no change; ≤20 mmol/L, increase 2 mmol/L; <18 mmol/L, increase 4 mmol/L (individualized)	24 months	31	• 7.4 ± 0.1 at baseline (mean dialysate bicarbonate 32 mmol/L) to 7.4 ± 0.1 at 24 months (mean dialysate bicarbonate 28 mmol/L).
	Cross-sectional	Wieliczko and Małyszko^14^	Dialysate bicarbonate mean 32.9 ± 1.8 mmol/L (range 28-36 mmol/L), titrated depending on pre-dialysis serum bicarbonate	Dialysis session	75	• Non-significant correlation between dialysate bicarbonate concentration and pre-HD pH.
		Movilli et al^39^	Dialysate bicarbonate 35 mmol/L; Group A daily oral sodium bicarbonate administration mean dose 1.9 ± 0.9 g/day (range 1-5 g/day), Group B control	4 weeks	110 (70 Group A, 40 Group B)	• 7.4 ± 0.02 in intervention group vs 7.3 ± 0.02 in control group, *P* = .001.
Post-dialysis pH	Randomized crossover trial	Di Iorio et al^21^	Dialysate bicarbonate 30 vs 34 mmol/L	Dialysis session	22	• pH was lower after study dialysis with bicarbonate 30 mmol/L than with bicarbonate 34 mmol/L (7.4 ± 0.03 vs 7.4 ± 0.05, *P* = .02).
		Gabutti et al^22^	Dialysate bicarbonate 26-29 vs 32-35 mmol/L	12 weeks	21	• 7.4 ± 0.06 (26-29 mmol/L), 7.5 ± 0.04 (32-25 mmol/L), *P* < .001.
		Gabutti et al^23^	3 dialysate modalities: A = bicarbonate 32 mmol/L, calcium 1.3 mmol/L, B = bicarbonate 26 mmol/L, calcium 1.3 mmol/L, C = bicarbonate 32 mmol/L, calcium 1.5 mmol/L	3 weeks	27	• 7.5 ± 0.05 (A), 7.4 ± 0.06 (B), 7.5 ± 0.05 (C).
		Gabutti et al^24^	Dialysate bicarbonate 32 vs 26 mmol/L	6 weeks	26	• 7.5 ± 0.05 (32 mmol/L) vs 7.4 ± 0.04 (26 mmol/L), *P* < .05.
		Williams et al^25^	Group A (dialysate bicarbonate 30 to 40 mmol/L), Group B (40 mmol/L to 30 mmol/L), and standard bicarbonate 35 mmol/L	12 months (crossover at 6 months)	46	• After 240-minute session, post-HD pH 7.5 ± 0.05 (35 mmol/L) vs 7.5 ±0.04 (B), *P* = .2.
	Non-randomized intervention	Sridhar et al^46^	4 dialysate solutions with either 38 or 32 mmol/L bicarbonate	Dialysis solution for 3 consecutive alternating day treatments over 1 week, outcomes measured after 1-week period	25	• Linear regression coefficient for dialysate bicarbonate 38 vs 32 mmol/L = 0.04, *P* < .05.
		Bozikas et al^40^	Dialysate/oral bicarbonate 37 mmol/L (period A) vs 35 mmol/L + 5 g/day (÷ 3 doses) (period B)	4 weeks	25	• Post-HD: 7.4 ± 0.04 at end of period A. • Post-HD: 7.4 ± 0.04 at end of period B.
	Prospective cohort	Noh et al^31^	Dialysate bicarbonate 25 vs 30 vs 35 mmol/L	64.4 ± 47 months	53	• 25 mmol/L group = 7.5 ± 0.05, 30 mmol/L group = 7.5 ± 0.09, 35 mmol/L group = 7.5 ± 0.04.
	Cross-sectional	Wieliczko and Małyszko^14^	Dialysate bicarbonate mean 32.9 ± 1.8 mmol/L (range 28-36 mmol/L), titrated depending on pre-dialysis serum bicarbonate	Dialysis session	75	• Non-significant correlation between bicarbonate bath and post-HD pH.
Pre-dialysis and post-dialysis total and ionized calcium	Randomized parallel-group trial	Rasheed et al^52^	Oral sodium bicarbonate (500 mg) daily	3 months	50 (25 intervention, 25 control)	• Pre-HD total calcium 2.1 ± 0.5 mmol/L in intervention group vs 2.0 ± 0.4 in control group (*P* = .4) at 3 months.
		Hefzollah et al^16^	Dialysate bicarbonate 36 vs 30 mmol/L	6 months	56 (26: 36 mmol/L, 30: 30 mmol/L)	• Pre-HD total calcium 2.1 ± 0.3 mmol/L at baseline to 2.1 ± 0.2 mmol/L (*P* = .9) (30 mmol/L) group. • Pre-HD total calcium 2.2 ± 0.1 mmol/L at baseline to 2.2 ± 0.2 mmol/L (*P* = .2) (36 mmol/L).
		Brady and Hasbargen^18^	40 mmol/L dialysate bicarbonate, plus 1 mmol/kg/d oral sodium bicarbonate if pre-dialysis serum bicarbonate <22 mmol/L after 2 weeks vs 35 mmol/L dialysate bicarbonate	16 weeks	38 (18: 40 mmol/L, 18: 35 mmol/L)	• Pre-HD total calcium 2.2 ± 0.2 mmol/L (35 mmol/L) vs 2.2 ± 0.4 mmol/L (40 mmol/L) at baseline (*P* > .05). • Pre-HD total calcium 2.3 ± 0.2 mmol/L (35 mmol/L) bicarbonate vs 2.1 ± 0.3 mmol/L (40 mmol/L) at study end (*P* > .05).
		Lefebvre et al^19^	Dialysate bicarbonate 33 ± 2 mmol/L control, intervention 7-15 mmol/L added targeting pre-dialysis serum bicarbonate 24 mmol/L	18 months	21 (11 intervention, 10 control)	• Pre-HD total calcium 2.3 ± 0.1 mmol/L in intervention group, 2.3 ± 0.03 mmol/L in control group, *P* > .05.^a^ • Post-HD total calcium 2.8 ± 0.08 mmol/L in intervention group, 3.0 ± 0.1 mmol/L in control group, *P* > .05.
	Randomized crossover trial	Di Iorio et al^21^	Dialysate bicarbonate 30 vs 34 mmol/L	4 hours	22	• Mean post-dialysis total calcium at end of study 2.2 ± 0.3 (K2 Ca 1.3 Bic 30) vs 2.0 ± 0.2 mmol/L (K2 Ca 1.5 Bic 34), *P* = .04; 2.3 ± 0.2 (K2 Ca 1.5 Bic 30) vs 2.2 ± 0.2 mmol/L (K2 Ca 1.5 Bic 34), *P* > .05; 2.6 ± 0.4 (K2 Ca 1.8 Bic 30) (*P* < .05 vs all solutions) vs 2.4 ± 0.3 mmol/L (K2 Ca 1.8 Bic 34); 2.2 ± 0.3 (K3 Ca 1.3 Bic 30) vs 2.1 ± 0.2 mmol/L (K3 Ca 1.3 Bic 34), *P* = .03; 2.3 ± 0.2 (K3 Ca 1.5 Bic 30) vs 2.2 ± 0.3 mmol/L (K3 Ca 1.5 Bic 34), *P* > .05; 2.6 ± 0.4 (K3 Ca 1.8 Bic 30) (*P* < .05 vs all solutions) vs 2.3 ± 0.3 mmol/L (K3 Ca 1.8 Bic 34). • At any dialysate calcium concentration (i.e., 1.3, 1.5, or 1.8 mmol/L), ionized plasma calcium during study dialysis significantly increased with 30 mmol/L dialysate bicarbonate (1.2 ± 0.1, 1.3 ± 0.1, and 1.3 ± 0.1 mmol/L, respectively) compared with 34 mmol/L dialysate bicarbonate (1.1 ± 0.1, 1.2 ± 0.1, and 1.3 ± 0.1 mmol/L, respectively; *P* < .006).
		Gabutti et al^22^	Dialysate bicarbonate 26-29 vs 32-35 mmol/L	12 weeks	21	• Pre-HD ionized calcium 1.1 ± 0.09 mmol/L with bicarbonate 26-29 and 1.1 ± 0.1 mmol/L with bicarbonate 32-35 (*P* > .05). • Post-HD ionized calcium 1.2 ± 0.08 mmol/L in bicarbonate 26-29 group, vs 1.1 ± 0.06 mmol/L in 32-35 group (*P* < .01).
		Gabutti et al^23^	3 dialysate modalities: A = bicarbonate 32 mmol/L, calcium 1.3 mmol/L, B = bicarbonate 26 mmol/L, calcium 1.3 mmol/L, C = bicarbonate 32 mmol/L, calcium 1.5 mmol/L	3 weeks	27	• Pre-HD ionized calcium 1.2 ± 0.1 mmol/L, 1.2 ± 0.1 mmol/L, and 1.1 ± 0.1 mmol/L in modality A, B, and C, respectively (no *P*-values reported). • Post-HD ionized calcium 1.1 ± 0.08 mmol/L, 1.2 ± 0.07 mmol/L, and 1.3 ± 0.07 mmol/L in modality A, B, and C, respectively (no *P*-values reported).
		Gabutti et al^24^	Dialysate bicarbonate 32 vs 26 mmol/L	6 weeks	26	• Pre-HD ionized calcium 1.2 ± 0.1 mmol/L (32 mmol/L) vs 1.2 ± 0.09 mmol/L (26 mmol/L) (*P* > .05). • Post-HD ionized calcium 1.3 ± 0.1 mmol/L (32 mmol/L), and 1.3 ± 0.1 mmol/L in (26 mmol/L) (*P* < .05).
		Williams et al^25^	Group A (dialysate bicarbonate 30 to 40 mmol/L), Group B (40 mmol/L to 30 mmol/L), and standard bicarbonate 35 mmol/L	12 months	46	• Pre-HD total calcium 2.6 ± 0.2 mmol/L at baseline and 2.5 ± 0.2 mmol/L at 12 months in Group A (*P* > .05). • Pre-HD total calcium 2.5 ± 0.2 mmol/L at baseline and 2.5 ± 0.2 mmol/L at 12 months in Group B (*P* > .05).
	Non-randomized intervention	Bozikas et al^40^	Dialysate/oral bicarbonate 37 mmol/L vs 35 mmol/L + 5 g/day (÷ 3 doses)	4 weeks	25	• Pre-HD ionized calcium 1.2 ± 0.07 mmol/L to post-HD 1.4 ± 0.4 mmol/L (*P* = .04) following dialysate bicarbonate 37 mmol/L treatment. • Pre-HD ionized calcium 1.2 ± 0.08 mmol/L to post-HD 1.3 ± 0.4 mmol/L (*P* = .005) following treatment with dialysate bicarbonate 35 mmol/L and oral sodium bicarbonate.
		Caruana et al^37^	Oral bicarbonate targeting mid-week pre-dialysis bicarbonate 22-26 mmol/L	1 month	20	• Total calcium 2.5 ± 0.2 mmol/L pre-therapy vs 2.5 ± 0.2 mmol/L post-therapy (*P* > .05); pre-dialysis/post-dialysis not specified. • Ionized calcium 1.3 ± 0.1 mmol/L pre-therapy vs 1.2 ± 0.08 post-therapy (*P* < .01); pre-dialysis/post-dialysis not specified.
		Bales et al^26^	Dialysate bicarbonate baseline 32 mmol/L, adjusted to maintain pre-dialysis serum bicarbonate ≥22 mmol/L	4 months	48	• Pre-HD ionized calcium 1.3 ± 0.1 mmol/L at month 0 vs 1.2 ± 0.1 mmol/L at month 4 (*P* = .002).
		Oettinger and Oliver^13^	Dialysate bicarbonate switched from 39 to 42 mmol/L (0-12 weeks) to 39 mmol/L (12-20 weeks)	20 weeks	38	• Pre-HD total calcium 2.4 ± 0.03 mmol/L at week 12 vs 2.3 ± 0.03 mmol/L at week 20 (*P* < .05). • Post-HD total calcium 2.3 ± 0.03 mmol/L at week 0 vs 2.4 ± 0.02 mmol/L at week 12 (*P* < .05); week 20 = not determined.
	Prospective cohort	Law and Davenport^45^	Dialysate bicarbonate 28 mmol/L versus 32 mmol/L	19 months	126	• Pre-HD total calcium 2.3 ± 0.2 mmol//L at baseline (dialysate bicarbonate 32 mmol/L) and 2.3 ± 0.2 mmol/L at study end (dialysate 28 mmol/L), *P* > .05.
		Cuadrado et al^47^	Dialysate bicarbonate individually modified according to pre-dialysis and post-dialysis serum bicarbonate targets of 19-25 mmol/L and 27-29 mmol/L (75% on 32-34 mmol/L dialysate bicarbonate at end of study)	6 months	123	• Pre-dialysis total calcium ranged from 2.1 ± 0.1 mmol/L at baseline to 2.3 ± 0.1 mmol/L (*P* < .001) at 3 months and 2.2 ± 0.1 mmol/L (*P* < .001) at 6 months. • The post-dialysis total calcium values ranged from 2.4 ± 0.2 at baseline to 2.5 ± 0.2 mmol/L (*P* < .001) at 3 months and 2.5 ± 0.2 mmol/L (*P* = .1) at 6 months.
		Montagud-Marrahi et al^28^	Dialysate bicarbonate 35 mmol/L at baseline switched to 32 mmol/L at study start	6 months	84	• Pre-HD total calcium 2.2 ± 0.1 mmol/L at baseline vs 2.2 ± 0.2 mmol/L at 6 months (*P* > .05). • Post-HD total calcium 2.5 ± 0.08 mmol/L at baseline vs 2.5 ± 0.2 mmol/L at 6 months (*P* > .05). • Calcium expressed as corrected calcium by albumin according to the formula: total calcium + (4—albumin) × 0.8, expressing calcium in mmol/L and albumin in g/dL.
		Valério Alves et al^50^	Dialysate bicarbonate adjusted every 3 months (9 time points): serum bicarbonate >30 mmol/L, reduce 4 mmol/L; ≥25 mmol/L, reduce 2 mmol/L; 20 mmol/L – 25 mmol/L, no change; ≤20 mmol/L, increase 2 mmol/L; <18 mmol/L, increase 4 mmol/L (individualized)	24 months	31	• 8.7 ± 0.6 mmol/L at baseline (mean dialysate bicarbonate 32 mmol/L) to 8.7 ± 0.5 mmol/L at 12 months, and median 8.9 mmol/L (IQR 0.5) at 24 months (mean dialysate bicarbonate 28 mmol/L); pre-dialysis/post-dialysis not specified.
		Panesar et al^29^	Dialysate bicarbonate 30-32, 33-34, 35-36, or 37-40 mmol/L	Not stated	39	• Pre-HD total calcium 4.8 ± 0.2 mmol/L, 5.0 ± 0.4 mmol/L, 5.0 ± 0.4 mmol/L, and 4.7 ± 0.2 mmol/L in 30-32, 33-34, 35-36, or 37-40 mmol/L groups, respectively (*P*-values not reported). • 68 hours post-HD total calcium 4.6 ± 0.2 mmol/L, 5.0 ± 0.4 mmol/L, 4.6 ± 0.5 mmol/L, and 4.7 ± 0.3 mmol/L in 30-32, 33-34, 35-36, or 37-40 mmol/L groups, respectively (*P*-values not reported).
		Blair et al^32^	Dialysate bicarbonate baseline 35 mmol/L increased to 39 mmol/L	6 months	197-199	• Pre-HD total calcium 9.2 ± 0.8 mmol/L (n = 197) at baseline vs 9.1 ± 0.8 mmol/L (n = 198) at 3 months, and 9.2 ± 0.9 mmol/L at 6 months (n = 199), *P* > .05.
	Retrospective cohort	Molnar et al^51^	Standardized dialysate bicarbonate (35, 36-37, and ≥38 mmol/L) and individualized dialysate bicarbonate concentration	1 year	5414	• Pre-HD total calcium 2.3 (0.2), 2.3 (0.2), 2.3 (0.2), 2.4 (0.2), and 2.3 (0.2) mmol/L in individualized group, combined standardized group all together, 35 mmol/L group, 36-37 mmol/L group, and ≥38 mmol/L group, respectively.
		Wan et al^44^	Dialysate bicarbonate <31.3 mmol/L, 31.3-32.3 mmol/L, 32.3-33.6 mmol/L, ≥33.6 mmol/L	Median 65 (range, 8-71) months	313	• Dialysate bicarbonate negatively associated with post-HD total calcium (r = −0.006, *P* > .01).
	Cross-sectional	Silva et al^30^	Dialysate bicarbonate (mean 38.2 ± 2.1 mmol/L)	Dialysis session	30	• Pre-HD ionized calcium 2.4 ± 0.3 mmol/L with dialysate bicarbonate ≤38 mmol/L vs 2.4 ± 0.2 mmol/L with dialysate bicarbonate >38 mmol/L (*P* = .6).
Potassium	Randomized parallel-group trial	Rasheed et al^52^	Oral sodium bicarbonate (500 mg) daily	3 months	50 (25 intervention, 25 control)	• 5.0 ± 0.4 mmol/L in intervention group vs 5.3 ± 0.6 mmol/L in control group (*P* < .05) at 3 months.
		Kourtellidou et al^15^	Average 3.7 ± 0.5 g oral sodium bicarbonate (maximum 6 g) daily	20 weeks	36 (16 intervention, 20 control)	• Pre-HD 4.8 ± 0.6 mmol/L in intervention group vs 5.2 ± 0.6 mmol/L in control group at weeks 13-16 (*P* = .08). • Post-HD 3.1 ± 0.4 mmol/L in intervention group vs 3.1 ± 0.3 mmol/L in control group at weeks 13-16 (*P* = .6).
		Hefzollah et al^16^	Dialysate bicarbonate 36 vs 30 mmol/L	6 months	56 (26: 36 mmol/L, 30: 30 mmol/L)	• Pre-HD 5.4 ± 0.9 mmol/L at baseline to 5.1 ± 0.8 mmol/L at 6 months (*P* = .1) (30 mmol/L). • Pre-HD 5.0 ± 0.9 mmol/L at baseline to 4.9 ± 0.6 mmol/L at 6 months (*P* = .8) (36 mmol/L)
		Brady and Hasbargen^18^	40 mmol/L dialysate bicarbonate, plus 1 mmol/kg/d oral sodium bicarbonate if pre-dialysis serum bicarbonate <22 mmol/L after 2 weeks vs 35 mmol/L dialysate bicarbonate	16 weeks	36 (18: 40 mmol/L, 18: 35 mmol/L)	• No significant difference between 35 mmol/L bicarbonate on pre-HD potassium (4.4 ± 0.6 mmol/L) vs 40 mmol/L on pre-HD potassium (4.5 ± 0.6 mmol/L) (*P* > .05).
		Lefebvre et al^19^	Dialysate bicarbonate 33 ± 2 mmol/L control, intervention 7-15 mmol/L added targeting pre-dialysis serum bicarbonate 24 mmol/L	18 months	21 (11 intervention, 10 control)	• Pre-HD potassium 5.5 ± 0.2 mmol/L at baseline to 5.2 ± 0.2 mmol/L in intervention group (*P* > .05 from baseline); pre-HD potassium 5.4 ± 0.2 mmol/L at baseline to 5.6 ± 0.1 mmol/L in control group (*P* > .05 from baseline). • Post-HD potassium 3.8 ± 0.1 mmol/L at baseline to 3.6 ± 0.1 mmol/L in intervention group (*P* > .05 from baseline); post-HD potassium 3.6 ± 0.1 mmol/L at baseline to 3.5 ± 0.1 mmol/L in control group (*P* > .05 from baseline).^a^
	Randomized crossover trial	Di Iorio et al^21^	Dialysate bicarbonate 30 vs 34 mmol/L	4 hours	22	• Post-HD potassium 3.1 ± 0.7 mmol/L (30 mmol/L) vs 3.2 ± 0.7 mmol/L (34 mmol/L) (*P* > .05).
		Gabutti et al^22^	Dialysate bicarbonate 26-29 vs 32-35 mmol/L	12 weeks	21	• Pre-HD potassium 5.0 ± 0.5 mmol/L with bicarbonate 26-29 vs 5.2 ± 1.6 mmol/L with bicarbonate 32-35 (*P* > .05). • Post-HD potassium 3.9 ± 0.2 mmol/L with bicarbonate 26-29 vs 3.7 ± 0.2 mmol/L with bicarbonate 32-35 (*P* < .001).
		Gabutti et al^23^	3 dialysate modalities: A = bicarbonate 32 mmol/L, calcium 1.3 mmol/L, B = bicarbonate 26 mmol/L, calcium 1.3 mmol/L, C = bicarbonate 32 mmol/L, calcium 1.5 mmol/L	3 weeks	27	• Pre-HD potassium 4.9 ± 0.6 mmol/L, 4.7 ± 0.7 mmol/L, and 4.8 ± 0.7 mmol/L with modalities A, B, and C, respectively. • Post-HD potassium 3.5 ± 0.4 mmol/L, 3.7 ± 0.4 mmol/L, and 3.5 ± 0.4 mmol/L with modalities A, B, and C, respectively (*P* < .05 A vs B).
		Gabutti et al^24^	Dialysate bicarbonate 32 vs 26 mmol/L	6 weeks	26	• Pre-HD potassium 4.8 ± 0.6 mmol/L with bicarbonate 32 vs 4.9 ± 0.6 mmol/L with bicarbonate 26 (*P* > .05). • Post-HD potassium 3.6 ± 0.4 mmol/L with bicarbonate 32 vs 3.9 ± 0.4 mmol/L with bicarbonate 26 (*P* < .01).
		Williams et al^25^	Group A (dialysate bicarbonate 30 to 40 mmol/L), Group B (40 mmol/L to 30 mmol/L), and standard bicarbonate 35 mmol/L	12 months (crossover a 6 months)	46	• Pre-HD 5.2 ± 0.6 mmol/L at baseline to 5.3 ± 0.6 mmol/L at 12 months in Group A. • Pre-HD 5.3 ± 0.8 mmol/L at baseline to 5.5 ± 0.7 mmol/L at 12 months in Group B.
	Non-randomized intervention	Bozikas et al^40^	Dialysate/oral bicarbonate 37 mmol/L (period A) vs 35 mmol/L + 5 g/day (÷ 3 doses) (period B)	4 weeks	25	• Pre-HD potassium 4.8 ± 0.7 mmol/L to post-HD 3.6 ± 0.3 mmol/L (*P* < .05) following bicarbonate 37. • Pre-HD potassium 4.8 ± 0.6 mmol/L to post-HD 3.6 ± 0.2 mmol/L (*P* < .05) following treatment with bicarbonate 35 and oral bicarbonate.
		Lin et al^27^	Dialysate bicarbonate increased from 35 to 38 mmol/L (month 1), then adjusted to 38-40 mmol/L in subgroup A (patients with pre-dialysis serum bicarbonate ≤21 mmol/L)	6 months	17	• Pre-HD baseline potassium 5.2 ± 0.2 mmol/L vs 5.0 ± 0.1 mmol/L at 6 months (A vs B, *P* < .05) in subgroup A (those with pre-dialysis serum bicarbonate ≤21 mmol/L).
	Prospective cohort	Cuadrado et al^47^	Dialysate bicarbonate individually modified according to pre-dialysis and post-dialysis serum bicarbonate targets of 19-25 mmol/L and 27-29 mmol/L (32 mmol/L at baseline, 75% on 32-34 mmol/L dialysate bicarbonate at end of study)	6 months	123	• Significant difference in pre-HD plasma potassium levels at baseline (4.7 ± 0.7 mmol/L) and 6 months (4.5 ± 0.7 mmol/L; *P* = .007). • Post-HD potassium significantly increased from 3.0 ± 0.4 mmol/L at baseline to 3.4 ± 0.8 mmol/L at 6 months (*P* < .001).
		Montagud-Marrahi et al^28^	Dialysate bicarbonate 35 mmol/L at baseline switched to 32 mmol/L at study start	6 months	84	• Pre-HD potassium 4.7 ± 0.5 mmol/L at baseline vs 4.7 ± 0.7 mmol/L at 6 months (*P* > .05). • Post-HD potassium 3.2 ± 0.3 mmol/L at baseline vs 3.0 ± 0.5 mmol/L at 6 months (*P* < .05).
		Panesar et al^29^	Dialysate bicarbonate 30-32, 33-34, 35-36, or 37-40 mmol/L	Not stated	39	• Pre-HD potassium 4.4 ± 0.4, 4.7 ± 0.8, 4.5 ± 0.3, and 4.3 ± 0.4 mmol/L in 30-32, 33-34, 35-36, or 37-40 mmol/L groups, respectively (*P* > .05 between-groups). • Immediate post-HD potassium 3.4 ± 0.2, 3.0 ± 0.3, 3.3 ± 0.2, and 3.2 ± 0.2 mmol/L in 30-32, 33-34, 35-36, or 37-40 mmol/L groups, respectively (*P* = .02 between 30-32 and 33-34 mmol/L groups). • 68 hours post-HD potassium 4.8 ± 0.5, 4.8 ± 0.7, 4.7 ± 0.5, and 4.7 ± 0.6 mmol/L across all concentrations, respectively (*P* > .05 between-groups).
		Blair et al^32^	Dialysate bicarbonate baseline 35 mmol/L increased to 39 mmol/L	6 months	182	• Pre-HD potassium 4.9 ± 0.7 mmol/L at baseline vs 4.8 ± 0.7 mmol/L at 6 months.
	Retrospective cohort	Molnar et al^51^	Standardized dialysate bicarbonate (35, 36-37, and ≥38 mmol/L) and individualized dialysate bicarbonate concentration	1 year	5414	• 4.7 (0.5), 4.7 (0.5), 4.8 (0.5), 4.7 (0.4), and 4.6 (0.5) mmol/L in individualized group, combined standardized group all together, 35 mmol/L group, 36-37 mmol/L group, and ≥38 mmol/L group, respectively.
		Wan et al^44^	Dialysate bicarbonate <31.3 mmol/L, 31.3-32.3 mmol/L, 32.3-33.6 mmol/L, ≥33.6 mmol/L	Median 65 (range, 8-71) months	313	• Dialysate bicarbonate negatively associated with post-HD potassium (r = −0.2, *P* = .002).
Parathyroid hormone (PTH)	Randomized parallel-group trial	Rasheed et al^52^	Oral sodium bicarbonate (500 mg) daily	3 months	50 (25 intervention, 25 control)	• iPTH 0.05 ± 0.04 pmol/L in intervention group vs 0.06 ± 0.04 pmol/L in control group (*P* = .8) at 3 months.
		Hefzollah et al^16^	Dialysate bicarbonate 36 vs 30 mmol/L	6 months	56 (26: 36 mmol/L, 30: 30 mmol/L)	• 51.1 ± 25.8 pmol/L at end of study compared to 47.7 ± 44.6 pmol/L at beginning of study, *P* = .8 (dialysate bicarbonate 30 mmol/L). • 49.7 ± 33.5 pmol/L at end of study compared to 47.0 ± 35.8 pmol/L at beginning of study, *P* = .7 (dialysate bicarbonate 36 mmol/L). • No *P*-values reported for between-group differences.
		Voiculeț et al^33^	Oral bicarbonate 5 g/day on non-dialysis days in intervention group; mean dialysate bicarbonate 29.8 (1.4) mmol/L intervention, 33 (2.2) mmol/L control	12 months	63 (29 intervention, 34 control)	• Initial vs final iPTH in intervention group (61.9 ± 44.8 vs 58.4 ± 43.3 pmol/L, *P* < .001). • Initial vs final iPTH in control group (64.8 ± 50.4 vs 68.8 ± 49.8 pmol/L, *P* < .001). • iPTH decreased in intervention (−3.5 ± 2.9, *P* < .001), increased in control group (4.0 ± 3.8, *P* < .001).
		Brady and Hasbargen^18^	40 mmol/L dialysate bicarbonate, plus 1 mmol/kg/d oral sodium bicarbonate if pre-dialysis serum bicarbonate <22 mmol/L after 2 weeks vs 35 mmol/L dialysate bicarbonate	16 weeks	36 (18: 40 mmol/L, 18: 35 mmol/L)	• iPTH 41.6 ± 35.7 pmol/L at baseline to 35.2 ± 29.8 pmol/L in 35 mmol/L group. • 36.9 ± 29.4 pmol/L at baseline to 30.9 ± 28.2 pmol/L (40 mmol/L) (*P* > .05 vs 35 mmol/L).
		Lefebvre et al^19^	Dialysate bicarbonate 33 ± 2 mmol/L control, intervention 7-15 mmol/L added targeting pre-dialysis serum bicarbonate 24 mmol/L	18 months	21 (11 intervention, 10 control)	• PTH (fragment 53-84) levels of 39.7 ± 8.8 pmol/L in control group vs 35.6 ± 7.5 pmol/L in intervention group.^a^
	Randomized crossover trial	Williams et al^25^	Group A (dialysate bicarbonate 30 to 40 mmol/L), Group B (40 mmol/L to 30 mmol/L), and standard bicarbonate 35 mmol/L	12 months (crossover at 6 months)	46	• 17 (1-150) pmol/L at baseline to 40 (1-109) pmol/L at 12 months in Group A (30 to 40 mmol/L) (*P* > .05). • 10 (2-105) pmol/L at baseline to 11 (1-83) pmol/L at 12 months in Group B (40 to 30 mmol/L) (*P* > .05).^d^ • No significant differences between groups at any timepoint.
	Non-randomized intervention	Bales et al^26^	Dialysate bicarbonate baseline 32 mmol/L, adjusted to maintain pre-dialysis serum bicarbonate ≥22 mmol/L	4 months	48	• Increase from month 0 (20.2 (9.0, 48.7) pmol/L) to month 4 (47.3 [22.5, 104.2] pmol/L) (*P* < .001).^c^
		Caruana et al^37^	Oral bicarbonate targeting mid-week pre-dialysis bicarbonate 22-26 mmol/L	1 month	20	• 58.0 ± 73.9 pmol/L at baseline vs 65.6 ± 82.3 pmol/L (*P* < .05).
	Non-randomized intervention	Lin et al^27^	Dialysate bicarbonate increased from 35 to 38 mmol/L (month 1), then adjusted to 38-40 mmol/L in subgroup A (patients with pre-dialysis serum bicarbonate ≤21 mmol/L)	6 months	17	• iPTH 34.6 ± 6.5 pmol/L at baseline vs 26.1 ± 5.2 pmol/L at 6 months (*P* < .05).
	Prospective cohort	Law and Davenport^45^	Dialysate bicarbonate 28 mmol/L versus 32 mmol/L	19 months	126	• PTH 32 (17-54) pmol/L at baseline (dialysate bicarbonate 32 mmol/L) and 40 (20-57) pmol/L at study end (dialysate 28 mmol/L), *P* > .05.
		Cuadrado et al^47^	Dialysate bicarbonate individually modified according to pre-dialysis and post-dialysis serum bicarbonate targets of 19-25 mmol/L and 27-29 mmol/L (32 mmol/L at baseline, 75% on 32-34 mmol/L dialysate bicarbonate at end of study)	6 months	123	• After the change of bicarbonate dialysate, PTH tended to increase, from 23.7 ± 19.7 pmol/L at baseline to 25.8 ± 15.6 pmol/L (*P* = .4) at month 3, although this was reversed by month 6 (22.0 ± 12.0 pmol/L; *P* = 0.9) and no statistical significance was achieved.
		Montagud-Marrahi et al^28^	Dialysate bicarbonate 35 mmol/L at baseline switched to 32 mmol/L at study start	6 months	84	• iPTH 24.0 (IQR 18.2-31.4) pmol/L at baseline vs 19.4 (IQR 13.3-25.3) pmol/L at 6 months (*P* < .05).
		Valério Alves et al^50^	Dialysate bicarbonate adjusted every 3 months (9 time points): dialysate bicarbonate >30 mmol/L. reduce 4 mmol/L; ≥25 mmol/L, reduce 2 mmol/L; 20 mmol/L – 25 mmol/L, no change; ≤20 mmol/L, increase 2 mmol/L; <18 mmol/L, increase 4 mmol/L (individualized)	24 months	31	• 24.2 (IQR 38.8) pmol/L at baseline (mean dialysate bicarbonate 32 mmol/L) to 19.2 (IQR 25.8) pmol/L at 24 months (mean dialysate bicarbonate 28 mmol/L).
		Blair et al^32^	Dialysate bicarbonate baseline 35 mmol/L increased to 39 mmol/L	6 months	163-197	• iPTH 0.3 ± 0.2 pmol/L (n = 197) baseline to 0.3 ± 0.2 pmol/L at 3 months (n = 166), 0.3 ± 0.2 pmol/L at 6 months (n = 163), *P* > .05.

*Note.* HD = hemodialysis, iPTH = intact parathyroid hormone, IQR = interquartile range, PTH = parathyroid hormone.

Conversion factors: Bicarbonate conventional unit (mEq/L) to SI unit (mmol/L) = 1, calcium conventional unit (mg/dL) to SI unit (mEq/L) = 0.50, calcium conventional unit (mg/dL) to SI unit (mmol/L) = 0.25, parathyroid hormone conventional unit (pg/mL) to SI unit (pmol/L) = 0.106, parathyroid hormone conventional unit (pg/dL) to SI unit (pmol/L) = 0.00106, parathyroid hormone conventional unit (ng/L) to SI unit (pmol/L) = 0.106, parathyroid hormone conventional unit (pg/L) to SI unit (pmol/L) = 0.000106, potassium conventional unit (mEq/L) to SI unit (mmol)/L = 1, sodium conventional unit (mEq/L) to SI unit (mmol)/L = 1. Values rounded to one decimal place or one significant figure. Data presented as mean and standard deviation unless otherwise indicated.

a Mean (SEM).

b Result obtained using WebPlotDigitizer.

c Median (IQR).

d Median (range).

**Figure 3.**
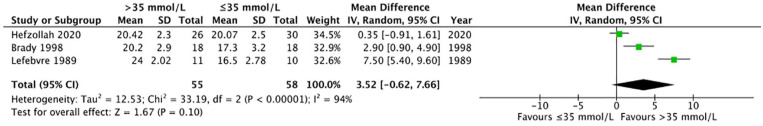
Forest plot of comparison: >35 mmol/L versus ≤35 mmol/L dialysate bicarbonate, outcome: pre-dialysis serum bicarbonate at end of study.

**Figure 4.**
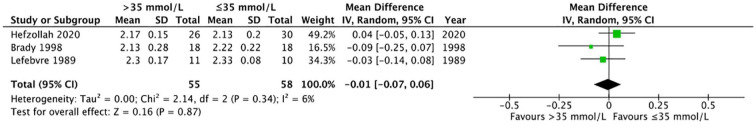
Forest plot of comparison: >35 mmol/L versus ≤35 mmol/L dialysate bicarbonate, outcome: pre-dialysis total calcium at end of study.^a^ ^a^Lefebvre et al^19^ standard deviation (SD) values converted from standard error of the mean (SEM)

**Figure 5.**
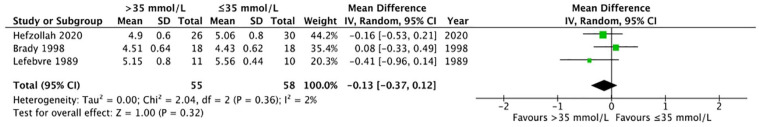
Forest plot of comparison: >35 mmol/L versus ≤35 mmol/L dialysate bicarbonate, outcome: pre-dialysis potassium at end of study.

Meta-analysis was performed on three HD parallel-group RCTs^16,18,19^ with 113 total participants that reported pre-dialysis serum bicarbonate, total calcium, and potassium at end of study follow-up and compared dialysate bicarbonate concentrations greater than 35 mmol/L to those ≤35 mmol/L (i.e., 30 vs 36 mmol/L, 35 vs 40 mmol/L, 33 ± 2 mmol/L vs additional 7-15 mmol/L). We found an uncertain effect of higher vs lower dialysate bicarbonate concentration on pre-dialysis serum bicarbonate (MD 3.5 mmol/L, 95% CI −0.6 to 7.7), serum total calcium (MD −0.01 mmol/L, 95% CI −0.07 to 0.6), and serum potassium (MD −0.1 mmol/L, 95% CI −0.4 to 0.1) (Figures 3-5, Supplemental Table 9). The overall certainty of the evidence for all three outcomes is very low due to serious concerns with risk of bias (randomization concerns), inconsistency (substantial statistical heterogeneity for serum bicarbonate, I^1^ = 94%), and imprecision (wide CIs and few participants).

Meta-analysis was also performed on three crossover RCTs^22-24^ with 74 total participants treated with HD that measured pre-dialysis and post-dialysis ionized calcium and potassium comparing dialysate bicarbonate concentrations ≥32 mmol/L and ≤29 mmol/L. There was an uncertain effect of higher vs lower dialysate bicarbonate concentration on pre-dialysis ionized calcium (MD 0.0 mmol/L, 95% CI −0.03 to 0.03), post-dialysis ionized calcium (MD −0.05 mmol/L, 95% CI −0.08 to −0.02), pre-dialysis serum potassium (MD −0.04 mmol/L, 95% CI −0.2 to 0.3), and post-dialysis serum potassium (MD −0.2 mmol/L, 95% CI −0.3 to −0.1) (Supplemental Figures 2-5, Supplemental Table 10). The overall certainty of the evidence is very low due to very serious concerns with risk of bias (selection bias and randomization concerns) and imprecision (few participants).

There were 14 HD/HDF studies that reported PTH as an outcome.^16,18,19,25-28,32,33,37,45,47,50,52^ Of these, the RCTs which compared higher to lower dialysate bicarbonate concentrations (n = 4) and oral sodium bicarbonate supplementation to no supplementation (n = 2), did not find significant differences in PTH,^16,18,19,25,52^ except for one RCT that found a decrease in iPTH in the oral bicarbonate group and an increase in iPTH in the control group.^33^ The largest PD RCT found higher serum bicarbonate and pH in the high dialysate alkali plus oral sodium bicarbonate group (27.2 ± 0.3 mmol/L, 7.4 ± 0.004) compared to the low dialysate alkali group (23.0 ± 0.3 mmol/L, 7.4 ± 0.004, both *P* < .001 between-groups) but no within-group differences in potassium, calcium, or PTH after one year (Supplemental Table 8).^48^

## Discussion

This systematic review included 41 studies, most of which included patients on maintenance HD or HDF. There were 16 RCTs, with the largest RCTs in HD and PD including 93 and 200 patients, respectively.^17,48^ Studies in HD/HDF examining mortality and hospitalizations were all non-randomized, with most evidence coming from one large, international cohort study demonstrating harm with higher dialysate bicarbonate concentrations.^3^ Studies in PD were small and lacked power to meaningfully determine effects on mortality and hard cardiovascular outcomes, such as stroke. The largest PD RCT reported fewer hospitalizations per patient per year in the high alkali group compared to the low alkali group.^48^

With respect to cardiovascular outcomes in HD/HDF RCTs, stroke and myocardial infarction were not reported, and none of the trials were powered to examine these effects. The parallel-group RCTs that we identified were few (n = 4) and small;^15,17,33,52^ most data were derived from small crossover RCTs and non-randomized interventional studies. Some studies suggest that higher dialysate bicarbonate concentrations may increase the risk of intradialytic hypotension and arrhythmia, but meaningful conclusions cannot be made due to small sample sizes and inconsistent results across studies.

Most studies reported no significant differences in nutritional outcomes, including outcomes of known prognostic value: serum albumin, nPCR, and SGA. These data suggest that dialysate bicarbonate concentrations within typically prescribed ranges, whether higher or lower, are sufficient to prevent protein-energy wasting related to metabolic acidosis. Evidence for the effect of higher vs lower dialysate bicarbonate concentrations on pre-HD serum bicarbonate, total calcium, and potassium was graded as very low certainty, as was the evidence for the effect on post-HD ionized calcium and potassium. As expected, most HD RCTs examining different dialysate bicarbonate concentrations reported a significantly higher post-dialysis serum bicarbonate with the use of higher dialysate bicarbonate concentrations,^17,19,21,24^ but the trial examining oral bicarbonate supplementation did not find a significant difference.^15^

A previous systematic review and meta-analysis^53^ on this topic included 3 RCTs^18,19,43^ and pooled both HD and PD studies.^18,43^ This approach may be problematic because we do not know whether the effect of dialysate bicarbonate or supplementation differs between PD and HD. This review is larger, including 41 studies and examined more diverse outcomes. The results of our meta-analysis of 3 HD RCTs^18,19,25^ yielded an uncertain effect of higher vs lower dialysate bicarbonate concentration on pre-dialysis serum bicarbonate (MD 3.5 mmol/L, 95% CI −0.6 to 7.7) and substantial heterogeneity (I^1^ = 94%). A previous systematic review and meta-analysis that pooled one PD and 2 HD RCTs with dialysate bicarbonate or oral bicarbonate intervention groups found a greater magnitude of effect (MD 4.6, 95% CI 1.1 to 8.2) but also substantial heterogeneity (I^1^ = 87.5%).^53^

Some of the heterogeneity between included studies could be explained by changes in characteristics of patients receiving maintenance dialysis through secular effects. For example, the mean age of participants in studies published in 1989 and 1998 was about 50 years,^18,19^ while more recent studies had a higher mean age of approximately 60 years.^16^ In addition, a study included in our meta-analysis published in 1989 excluded patients with diabetes,^19^ while contemporary study populations included up to 70% of participants with diabetes.^16^ The evidence is also limited by small sample sizes of published RCTs; the largest HD and PD trial sample sizes were 93 and 200 patients, respectively and had a high risk of bias^17^ or some concerns. Most HD/HDF studies had a moderate or high risk of bias, while 2 of 4 PD studies had a low risk of bias. Sufficient data were not available to meta-analyze patient-important outcomes, such as myocardial infarction, stroke, mortality and hospitalizations. HD studies examining nutritional outcomes and surrogate cardiovascular outcomes were too heterogeneous in terms of methods and outcome definitions for meta-analysis.

A strength of this systematic review is the use of broad inclusion criteria accepting any type of adult population on maintenance HD/HDF or PD that reported any comparisons of dialysate bicarbonate concentration or oral bicarbonate supplementation with no supplementation with no limitations on study design. This review also focused on a wide range of biochemical and clinically relevant outcomes that would be plausibly affected by alterations in bicarbonate status and commonly used by nephrologists to guide management decisions. Limitations of the review include the exclusion of non-English literature and gray literature, which might introduce publication bias. However, the magnitude of this bias may not be large, and the quantity and quality of the data found were such that it is unlikely that we missed work that would change our conclusions.^54^ Another limitation of this review is the lack of a registered protocol, which may affect transparency. However, there were no deviations from the initial review plan. Additionally, this review relied primarily on small or observational studies, as there was a lack of large RCTs.

## Conclusions

In conclusion, current guidelines for treatment of metabolic acidosis in kidney failure recommend a serum bicarbonate ≥22 mmol/L.^2^ This recommendation has resulted in the common use of high (≥35 mmol/L) dialysate bicarbonate concentrations or, less commonly, the use of oral bicarbonate supplementation, with the assumption that these approaches will normalize serum bicarbonate (measured pre-dialysis in HD patients), and therefore improve patient outcomes.^3^ The results of this review support that this recommendation and the interventions used to treat the metabolic acidosis of kidney failure are based on limited, weak evidence. This review highlights the need for large, high-quality RCTs that examine the impact of higher vs lower dialysate bicarbonate concentrations or oral bicarbonate supplementation on clinically relevant outcomes in patients receiving maintenance dialysis.

## Supplemental Material

sj-docx-1-cjk-10.1177_20543581251356182 – Supplemental material for The Effect of Dialysate Bicarbonate Concentration or Oral Bicarbonate Supplementation on Outcomes in Patients on Maintenance Dialysis: A Systematic Review and Meta-Analysis Supplemental material, sj-docx-1-cjk-10.1177_20543581251356182 for The Effect of Dialysate Bicarbonate Concentration or Oral Bicarbonate Supplementation on Outcomes in Patients on Maintenance Dialysis: A Systematic Review and Meta-Analysis by Ashlee M. Azizudin, Samuel A. Silver, Amit X. Garg, Zoe K. Friedman, Andrea C. Cowan, Catherine M. Clase and Amber O. Molnar in Canadian Journal of Kidney Health and Disease

sj-docx-2-cjk-10.1177_20543581251356182 – Supplemental material for The Effect of Dialysate Bicarbonate Concentration or Oral Bicarbonate Supplementation on Outcomes in Patients on Maintenance Dialysis: A Systematic Review and Meta-Analysis Supplemental material, sj-docx-2-cjk-10.1177_20543581251356182 for The Effect of Dialysate Bicarbonate Concentration or Oral Bicarbonate Supplementation on Outcomes in Patients on Maintenance Dialysis: A Systematic Review and Meta-Analysis by Ashlee M. Azizudin, Samuel A. Silver, Amit X. Garg, Zoe K. Friedman, Andrea C. Cowan, Catherine M. Clase and Amber O. Molnar in Canadian Journal of Kidney Health and Disease

## References

[1] Ikizler TA Burrowes JD Byham-Gray LD , et al. KDOQI clinical practice guideline for nutrition in CKD: 2020 update. Am J Kidney Dis. 2020;76(3):S1-S107. 10.1053/j.ajkd.2020.05.006 32829751

[2] K/DOQI National Kidney Foundation. Clinical practice guidelines for nutrition in chronic renal failure. Am J Kidney Dis. 2000;35:S17-S104. 10.1053/ajkd.2000.v35.aajkd03517 10895784

[3] Tentori F Karaboyas A Robinson BM , et al. Association of dialysate bicarbonate concentration with mortality in the Dialysis Outcomes and Practice Patterns Study (DOPPS). Am J Kidney Dis. 2013;62(4):738-746. 23707043 10.1053/j.ajkd.2013.03.035 PMC3832240

[4] Page MJ McKenzie JE Bossuyt PM , et al. The PRISMA 2020 statement: an updated guideline for reporting systematic reviews. BMJ. 2021;372:n71. 10.1136/bmj.n71 PMC8005924 33782057

[5] Rohatgi A . Webplotdigitizer: version 4.4. 2020. https://automeris.io/. Accessed June 25, 2025.

[6] Drevon D Fursa SR Malcolm AL . Intercoder reliability and validity of WebPlotDigitizer in extracting graphed data. Behav Modif. 2017;41(2):323-339. 27760807 10.1177/0145445516673998

[7] Sterne JAC Savović J Page MJ , et al. RoB 2: a revised tool for assessing risk of bias in randomised trials. BMJ. 2019;366:l4898. 10.1136/bmj.l4898 31462531

[8] Sterne JA Hernán MA Reeves BC , et al. ROBINS-I: a tool for assessing risk of bias in non-randomised studies of interventions. BMJ. 2016;355:i4919. 10.1136/bmj.i4919 PMC5062054 27733354

[9] Wells G Shea B O’Connell D , et al. The Newcastle-Ottawa Scale (NOS) for assessing the quality of nonrandomised studies in meta-analyses, 2013. http://www.ohri.ca/programs/clinical_epidemiology/oxford.asp/. Accessed June 25, 2025.

[10] Moola S Munn Z Tufanaru C , et al. Systematic reviews of etiology and risk. In: Aromataris E Lockwood C Porritt K Pilla B Jordan Z , eds. JBI Manual for Evidence Synthesis. Adelaide, SA, Australia: JBI; 2020:252-311.

[11] Guyatt GH Oxman AD Kunz R , et al. What is “quality of evidence” and why is it important to clinicians? BMJ. 2008;336:995. 18456631 10.1136/bmj.39490.551019.BE PMC2364804

[12] Terrin N Schmid CH Lau J . In an empirical evaluation of the funnel plot, researchers could not visually identify publication bias. J Clin Epidemiol. 2005;58(9):894-901. 16085192 10.1016/j.jclinepi.2005.01.006

[13] Oettinger CW Oliver JC . Normalization of uremic acidosis in hemodialysis patients with a high bicarbonate dialysate. J Am Soc Nephrol. 1993;3(11):1804-1807. 8329675 10.1681/ASN.V3111804

[bibr14-20543581251356182] Wieliczko M Małyszko J . Acid–base balance in hemodialysis patients in everyday practice. Ren Fail. 2022;44(1):1090-1097. 35793495 10.1080/0886022X.2022.2094805 PMC9272922

[bibr15-20543581251356182] Kourtellidou SI Ashby DR Johansson LR . Oral sodium bicarbonate in people on haemodialysis: a randomised controlled trial. BMC Nephrol. 2021;22(1):346. 34674667 10.1186/s12882-021-02549-x PMC8529780

[bibr16-20543581251356182] Hefzollah F Boushehri SN Mahmudpour M . Effect of high bicarbonate hemodialysis solution on biochemical parameters and anthropometric indices. Hemodial Int. 2020;24(3):317-322. 32419310 10.1111/hdi.12842

[bibr17-20543581251356182] Viegas M Cândido C Felgueiras J , et al. Dialysate bicarbonate variation in maintenance hemodiafiltration patients: impact on serum bicarbonate, intradialytic hypotension and interdialytic weight gain. Hemodial Int. 2017;21(3):385-392. 27761981 10.1111/hdi.12502

[bibr18-20543581251356182] Brady JP Hasbargen JA . Correction of metabolic acidosis and its effect on albumin in chronic hemodialysis patients. Am J Kidney Dis. 1998;31(1):35-40. 9428449 10.1053/ajkd.1998.v31.pm9428449

[bibr19-20543581251356182] Lefebvre A de Vernejoul MC Gueris J Goldfarb B Graulet AM Morieux C . Optimal correction of acidosis changes progression of dialysis osteodystrophy. Kidney Int. 1989;36(6):1112-1118. 2557481 10.1038/ki.1989.309

[bibr20-20543581251356182] Sabzghabaei F Heydariezade SA Joodat RS . The effects of different electrolyte composition in dialysate on QTc interval; a controlled trial. J Renal Inj Prev. 2016;5(3):153-156. 27689113 10.15171/jrip.2016.32 PMC5040003

[bibr21-20543581251356182] Di Iorio B Torraca S Piscopo C , et al. Dialysate bath and QTc interval in patients on chronic maintenance hemodialysis: pilot study of single dialysis effects. J Nephrol. 2012;25(5):653-660. 21983985 10.5301/jn.5000036

[bibr22-20543581251356182] Gabutti L Bianchi G Soldini D Marone C Burnier M . Haemodynamic consequences of changing bicarbonate and calcium concentrations in haemodialysis fluids. Nephrol Dial Transplant. 2009;24(3):973-981. 18842671 10.1093/ndt/gfn541 PMC2644633

[bibr23-20543581251356182] Gabutti L Ross V Duchini F Mombelli G Marone C . Does bicarbonate transfer have relevant hemodynamic consequences in standard hemodialysis. Blood Purif. 2005;23(5):365-372. 16088104 10.1159/000087193

[bibr24-20543581251356182] Gabutti L Ferrari N Giudici G Mombelli G Marone C . Unexpected haemodynamic instability associated with standard bicarbonate haemodialysis. Nephrol Dial Transplant. 2003;18(11):2369-2376. 14551368 10.1093/ndt/gfg383

[bibr25-20543581251356182] Williams AJ Dittmer ID McArley A Clarke J . High bicarbonate dialysate in haemodialysis patients: effects on acidosis and nutritional status. Nephrol Dial Transplant. 1997;12(12):2633-2637. 9430864 10.1093/ndt/12.12.2633

[bibr26-20543581251356182] Bales AM Moysés RM dos Reis LM , et al. Correction of metabolic acidosis in hemodialysis: consequences on serum leptin and mineral metabolism. Int Urol Nephrol. 2015;47(1):177-182. 25252843 10.1007/s11255-014-0844-5

[bibr27-20543581251356182] Lin SH Lin YF Chin HM Wu CC . Must metabolic acidosis be associated with malnutrition in haemodialysed patients. Nephrol Dial Transplant. 2002;17(11):2006-2010. 12401862 10.1093/ndt/17.11.2006

[bibr28-20543581251356182] Montagud-Marrahi E Broseta J Rodriguez-Espinosa D , et al. Optimization of dialysate bicarbonate in patients treated with online haemodiafiltration. Clin Kidney J. 2020;14(3):1003-1013. 10.1093/ckj/sfaa058 PMC8204783 34141409

[bibr29-20543581251356182] Panesar M Shah N Vaqar S , et al. Changes in serum bicarbonate levels caused by acetate-containing bicarbonate-buffered hemodialysis solution: an observational prospective cohort study. Ther Apher Dial. 2017;21(2):157-165. 28296160 10.1111/1744-9987.12510

[bibr30-20543581251356182] Silva BC Freitas GR Silva VB , et al. Hemodynamic behavior during hemodialysis: effects of dialysate concentrations of bicarbonate and potassium. Kidney Blood Press Res. 2014;39(5):490-496. 25532082 10.1159/000368459

[bibr31-20543581251356182] Noh US Yi JH Han SW Kim HJ . Varying dialysate bicarbonate concentrations in maintenance hemodialysis patients affect post-dialysis alkalosis but not pre-dialysis acidosis. Electrolyte Blood Press. 2007;5(2):95101. 10.5049/EBP.2007.5.2.95 PMC3894521 24459507

[bibr32-20543581251356182] Blair D Bigelow C Sweet SJ . Nutritional effects of delivered bicarbonate dose in maintenance hemodialysis patients. J Ren Nutr. 2003;13(3):205-211. 12874745 10.1016/s1051-2276(03)00092-x

[bibr33-20543581251356182] Voiculeț C Zară O Bogeanu C Văcăroiu I Aron G . The role of oral sodium bicarbonate supplementation in maintaining acid—base balance and its influence on the cardiovascular system in chronic hemodialysis patients—results of a prospective study. J Med Life. 2016;9(4):449-454. 27928454 PMC5141410

[bibr34-20543581251356182] Sajgure AD Dighe TA Korpe JS , et al. The relationship between metabolic acidosis and nutritional parameters in patients on hemodialysis. Indian J Nephrol. 2017;27(3):190-194. 28553038 10.4103/0971-4065.202404 PMC5434684

[bibr35-20543581251356182] Movilli E Viola BF Camerini C Mazzola G Cancarini GC . Correction of metabolic acidosis on serum albumin and protein catabolism in hemodialysis patients. J Ren Nutr. 2009;19(2):172-177. 19218045 10.1053/j.jrn.2008.08.012

[bibr36-20543581251356182] Bossola M Giungi S Tazza L Luciani G . Long-term oral sodium bicarbonate supplementation does not improve serum albumin levels in hemodialysis patients. Nephron Clin Pract. 2007;106(1):c51-c56. 10.1159/000101484 17409769

[bibr37-20543581251356182] Caruana RJ Weinstein RS Campbell HT Chaudhary BA Smith KL Kurunsaari KM . Effects of oral base therapy on serum ionized calcium, phosphorus and parathyroid hormone in chronic hemodialysis patients. Int J Artif Organs. 1989;12(12):778-781. 2559040

[bibr38-20543581251356182] Checheriţă IA David C Ciocâlteu A Lascăr I Budală L . Oral treatment of metabolic acidosis in hemodialyzed patients and the implications on the hemodynamic status. Rom J Morphol Embryol. 2013;54(3):539-543. 24068401

[bibr39-20543581251356182] Movilli E Gaggia P Camerini C Brunori G Vizzardi V Cancarini G . Effect of oral sodium bicarbonate supplementation on interdialytic weight gain, plasma sodium concentrations and predialysis blood pressure in hemodialysis patients: a cross-sectional and interventional study. Blood Purif. 2005;23(5):379-383. 16088106 10.1159/000087195

[bibr40-20543581251356182] Bozikas A Kiriakoutzik I Petrou I , et al. Aiming for the optimal bicarbonate prescription for maintenance hemodialysis therapy in end-stage renal disease. Hemodial Int. 2019;23(2):173-180. 30762289 10.1111/hdi.12710

[bibr41-20543581251356182] Feriani M Passlick-Deetjen J Jaeckle-Meyer I La Greca G , Study Group. Individualized bicarbonate concentrations in the peritoneal dialysis fluid to optimize acid-base status in CAPD patients. Nephrol Dial Transplant. 2004;19(1):195-202. 14671057 10.1093/ndt/gfg472

[bibr42-20543581251356182] Liu XY Gao XM Zhang N , et al. Oral bicarbonate slows decline of residual renal function in peritoneal dialysis patients. Kidney Blood Press Res. 2017;42(3):565-574. 29032379 10.1159/000479641

[bibr43-20543581251356182] Szeto CC Wong TY Chow KM Leung CB Li PK . Oral sodium bicarbonate for the treatment of metabolic acidosis in peritoneal dialysis patients: a randomized placebo-control trial. J Am Soc Nephrol. 2003;14(8):2119-2126. 12874466 10.1097/01.asn.0000080316.37254.7a

[bibr44-20543581251356182] Wan J Lin J Wang W , et al. Relationship between dialysate bicarbonate concentration and all-cause mortality in hemodialysis patients. Kidney Blood Press Res. 2023;48(1):460-467. 37253349 10.1159/000531267

[bibr45-20543581251356182] Law S Davenport A . The effect of changing dialysate bicarbonate concentration on serum bicarbonate, body weight and normalized nitrogen appearance rate. Artif Organs. 2023;47(5):891-897. 36519969 10.1111/aor.14483

[bibr46-20543581251356182] Sridhar NR Chen Z Yu G , et al. Effect of dialysate bicarbonate and sodium on blood pH in maintenance hemodialysis-a prospective study. Ther Apher Dial. 2023;27(2):270-277. 36056807 10.1111/1744-9987.13920

[bibr47-20543581251356182] Cuadrado E Broseta JJ Rodríguez-Espinosa D , et al. Tailoring the dialysate bicarbonate eliminates pre-dialysis acidosis and post-dialysis alkalosis. Clin Kidney J. 2022;15(10):1946-1951. 36158145 10.1093/ckj/sfac128 PMC9494532

[bibr48-20543581251356182] Stein A Moorhouse J Iles-Smith H , et al. Role of an improvement in acid-base status and nutrition in CAPD patients. Kidney Int. 1997;52(4):1089-1095. 9328950 10.1038/ki.1997.433

[bibr49-20543581251356182] Ravi KS Tumlin JA Roy-Chaudhury P , et al. Association of dialysate bicarbonate with arrhythmia in the Monitoring in Dialysis (MiD) study. Kidney360. 2024;5(10):1490-1499. 39480910 10.34067/KID.0000000000000537 PMC11556930

[bibr50-20543581251356182] Valério Alves R Gonçalves H Lopes K Sofia F Vila Lobos A . Changing the paradigm of bicarbonate (HCO3−) hemodialysis prescription in Portugal: a 24-month prospective study. Ren Replace Ther. 2020;6(1):50.

[bibr51-20543581251356182] Molnar AO Killin L Bota S , et al. Association between the dialysate bicarbonate and the pre-dialysis serum bicarbonate concentration in maintenance hemodialysis: a retrospective cohort study. Can J Kidney Health Dis. 2024;11:20543581241256774. 10.1177/20543581241256774 PMC11141227 38827142

[52] Rasheed ZA Al-Hashemi BA Ali AA . Effects of oral sodium bicarbonate supplementation on protein metabolism and inflammation in Iraqi hemodialysis patients: an open-label randomized controlled trial. Int J Nephrol. 2023;2023:6657188. 37545875 10.1155/2023/6657188 PMC10403331

[53] Roderick P Willis NS Blakeley S Jones C Tomson C . Correction of chronic metabolic acidosis for chronic kidney disease patients. Cochrane Database Syst Rev. 2007;2007(1):CD001890. 10.1002/14651858.CD001890.pub3 PMC7045985 17253467

[54] Nussbaumer-Streit B Klerings I Dobrescu AI , et al. Excluding non-English publications from evidence-syntheses did not change conclusions: a meta-epidemiological study. J Clin Epidemiol. 2020;118:42-54. 31698064 10.1016/j.jclinepi.2019.10.011

