# Supplementary material for: The Effect of Dialysate Bicarbonate Concentration or Oral Bicarbonate Supplementation on Outcomes in Patients on Maintenance Dialysis: A Systematic Review and Meta-Analysis
Source: Can J Kidney Health Dis. 2025 Jul 31;12:20543581251356182. doi: 10.1177/20543581251356182 (PMC12317238; doi:10.1177/20543581251356182)
Supplement: sj-docx-1-cjk-10.1177_20543581251356182 – Supplemental material for The Effect of Dialysate Bicarbonate Concentration or Oral Bicarbonate Supplementation on Outcomes in Patients on Maintenance Dialysis: A Systematic Review and Meta-Analysis [file sj-docx-1-cjk-10.1177_20543581251356182.docx]

Studies from databases/registers **(n = 4221)**

MEDLINE (n = 1665)

Embase (n = 1558)

CENTRAL (n = 798)

Google Scholar (n = 200)

References from other sources **(n = 0)**

Studies included in review **(n = 41)**

Studies excluded **(n = 3311)**

Studies not retrieved **(n = 0)**

Studies assessed for eligibility **(n = 344)**

Studies sought for retrieval **(n = 344)**

Studies screened **(n = 3655)**

**Identification**

References removed **(n = 566)**

Duplicates identified (n = 566)

**Screening**

Studies excluded **(n = 303)**

Review article (n = 48)

Wrong intervention (n = 40)

Abstract only (n = 39)

Investigating serum bicarbonate exposure, not dialysate/oral bicarbonate (n=37)

<20 participants (n = 36)

No outcomes of interest (n = 25)

Wrong comparator (n = 13)

Wrong patient population (n = 13)

Wrong study design (n = 11)

Study protocol (n = 9)

Duplicate/abstract version (n = 6)

Editorial (n = 6)

Letter to the Editor (n = 6)

Case presentation (n = 4)

Commentary (n = 3)

Non-English article (n = 2)

Long term follow-up from previously published results (n = 1)

Paediatric population (n = 1)

Trial registry (n = 1)

Unable to retrieve article (n = 1)

In vitro (n = 1)

**Included**
