# Supplementary material for: The Effect of Dialysate Bicarbonate Concentration or Oral Bicarbonate Supplementation on Outcomes in Patients on Maintenance Dialysis: A Systematic Review and Meta-Analysis
Source: Can J Kidney Health Dis. 2025 Jul 31;12:20543581251356182. doi: 10.1177/20543581251356182 (PMC12317238; doi:10.1177/20543581251356182)
Supplement: sj-docx-2-cjk-10.1177_20543581251356182 – Supplemental material for The Effect of Dialysate Bicarbonate Concentration or Oral Bicarbonate Supplementation on Outcomes in Patients on Maintenance Dialysis: A Systematic Review and Meta-Analysis [file sj-docx-2-cjk-10.1177_20543581251356182.docx]

**Table of Contents**

[Supplemental Table 1. Characteristics of HD/HDF studies 2](#_Toc187449426)

[Supplemental Table 2. Characteristics of PD studies 9](#_Toc187449427)

[Supplemental Table 3. Quality of observational cohort studies 10](#_Toc187449428)

[Supplemental Table 4. Risk of bias of cross-sectional studies 11](#_Toc187449429)

[Supplemental Table 5. Cardiovascular outcomes in PD studies 11](#_Toc187449430)

[Supplemental Table 6. Nutritional outcomes in HD/HDF studies 12](#_Toc187449431)

[Supplemental Table 7. Nutritional outcomes in PD studies 15](#_Toc187449432)

[Supplemental Table 8. Serum and plasma bicarbonate, pH, calcium, potassium, and PTH in PD studies 17](#_Toc187449433)

[Supplemental Table 9. Summary of meta-analysis findings comparing dialysate bicarbonate > 35 mmol/L to ≤ 35 mmol/L 19](#_Toc187449434)

[Supplemental Table 10. Summary of meta-analysis findings comparing dialysate bicarbonate ≥ 32 mmol/L to ≤ 29 mmol/L 20](#_Toc187449435)

[Supplemental Figure 1. PRISMA Flow Diagram 22](#_Toc187449373)

[Supplemental Figure 2. Forest plot of comparison: ≥ 32 mmol/L versus ≤ 29 mmol/L dialysate bicarbonate, outcome: Pre-dialysis ionized calcium. 22](#_Toc187449374)

[Supplemental Figure 3. Forest plot of comparison: ≥ 32 mmol/L versus ≤ 29 mmol/L dialysate bicarbonate, outcome: Post-dialysis ionized calcium. 22](#_Toc187449375)

[Supplemental Figure 4. Forest plot of comparison: ≥ 32 mmol/L versus ≤ 29 mmol/L dialysate bicarbonate, outcome: Pre-dialysis potassium. 22](#_Toc187449376)

[Supplemental Figure 5. Forest plot of comparison: ≥ 32 mmol/L versus ≤ 29 mmol/L dialysate bicarbonate, outcome: Post-dialysis potassium. 23](#_Toc187449377)

[Appendix A: PRISMA Statement 23](#_Toc181295365)

[Appendix B: Search Strategy 25](#_Toc181295366)

[Appendix C. Data Abstraction Form 26](#_Toc181295367)

**Supplemental Table 1. Characteristics of HD/HDF studies**

| **Study, year** | **Country** | **Study Design** | **Inclusion Criteria** | **Exclusion Criteria** | **Dialysis vintage**  **Mean (SD)** | **Intervention/Exposure and Comparator (Fixed or Individualized approach to dialysate or oral bicarbonate)** | **Outcomes** | **Number of patients** | **Age of participants in years¶**  **Mean (SD)** |
| --- | --- | --- | --- | --- | --- | --- | --- | --- | --- |
| Molnar, 2024 | Canada | Retrospective cohort | Adults receiving maintenance in-center HD at the same regional dialysis program for at least 120 days as of April 1, 2020 | Patients receiving in-center short daily dialysis or nocturnal hemodialysis at baseline, patients from 2 Ontario regional dialysis programs with no laboratory data available through the Ontario Libraries Information System (OLIS) | Not reported | Dialysate individualized (adjustment based on pre-dialysis serum bicarbonate concentration) or standardized (>90% of patients received the same dialysate bicarbonate concentration). The standardized category stratified by concentration: 35, 36-37, and ≥38 mmol/L | Pre-dialysis serum bicarbonate, serum calcium, serum potassium, serum albumin | 5414 | 68 (14) |
| Ravi, 2024 | USA | Prospective cohort | ≥ 21 years of age, thrice-weekly in-center HD, or eGFR <15ml/min/1.73 m^2^ with expected HD initiation within two months | Unsuitability for implantation, expected survival <6 months, left-sided HD catheter interfering with implantation, thoracic surgery within preceding 6 months, bacteremia within preceding 60 days or non-bacteremic infection within preceding 14 days, hemoglobin <10 g/dL on consecutive measurements within the preceding 2 months, end-stage liver failure, transplantation or modality transfer expected within 6 months, existing pacemaker or implantable cardioverter defibrillator | 2.4 (1.2, 5.3) years | Dialysate bicarbonate <35, 35, and >35 mmol/L (fixed) | Arrhythmia | 66 | 56 (12) |
| Law and Davenport, 2023 | UK | Prospective cohort | Receiving HD | None specified | Not reported | Dialysate bicarbonate lowered from 32 mmol/L to 28 mmol/L (fixed) | Lean tissue index, albumin, nPNA, calcium, pre- and post-dialysis serum bicarbonate, PTH | 126 | 64 (16) |
| Sridhar, 2023 | USA | Non-randomized interventional study | HD patients with arteriovenous fistulae or grafts in outpatient setting | None specified | 4.1 (2.5) years | Dialysate 140 mmol/L sodium/38 mmol/L bicarbonate/3.9 mmol/L acetate; 140 mmol/L sodium/32 mmol/L bicarbonate/4.2 mmol/L acetate; 137 mmol/L sodium/38 mmol/L bicarbonate/3.8 mmol/L acetate; 137 mmol/L sodium/32 mmol/L bicarbonate/4.0 mmol/L acetate (fixed) | Pre- and post-dialysis pH | 25 | 63 (17) |
| Rasheed, 2023 | Iraq | Randomized parallel-group trial | Adults on regular HD for >3 months, HD 3x/week, documented serum bicarbonate level of <22 mmol/L, dialysate bicarbonate concentration of 35 mmol/L, no residual renal function (24 hours urine output <200 ml), arteriovenous fistulae as dialysis access, steady clinical state | Acute illness or infection in the last 3 months, anticipated life expectancy of 6 months, advanced senility and impaired cognition, clinically evident cachexia and sarcopenia, ongoing enteral or parenteral nutrition, uncontrolled blood pressure (>160/90), predialysis potassium levels of less than 4 mmol/L, steroids or immunosuppressive agents, already using oral sodium bicarbonate therapy | 2.3 (0.6) years intervention, 2.0 (0.6) years control | Oral sodium bicarbonate (500 mg) daily | nPCR, serum albumin, systolic blood pressure, diastolic blood pressure, pre-dialysis serum bicarbonate, serum potassium, calcium, iPTH, hand grip strength, triceps skinfold thickness | 50 | 47 (18) intervention, 50 (14) control |
| Wan, 2023 | China | Retrospective cohort study | ≥18 years of age receiving HD three times per week, 4 h per session; and dialysis for more than 3 months | Combination therapy with peritoneal dialysis, incomplete bicarbonate concentration data (i.e., participants who missed one or more of the follow-up measurements, including those of dialysate bicarbonate, pre-HD serum bicarbonate, or post-HD serum bicarbonate) | 65 (range, 8–71) months* | Dialysate bicarbonate <31.3 mmol/L, 31.3–32.3 mmol/L, 32.3–33.6 mmol/L, ≥33.6 mmol/L (individualized- adjusted to a target pre-dialysis serum bicarbonate 22-26 mmol/L) | All-cause mortality, first hospitalization for any cause | 313 | 61 (14) |
| Cuadrado, 2022 | Spain | Prospective cohort | Receiving HDF or expanded HD for ≥12 months under a regular prescription of 32 mmol/L dialysate | Temporary percutaneous catheters, awaiting living donor kidney transplant, immunosuppressive treatment, on oral bicarbonate supplements, significant residual kidney function | 53.4 (5.9) months | Dialysate bicarbonate individually modified according to pre- and post-dialysis total carbon dioxide targets of 19-25 mmol/L and 27-29 mmol/L (individualized) | Pre- and post-dialysis potassium, calcium, parathyroid hormone | 123 | 70 (16) |
| Wieliczko, 2022 | Poland | Cross-sectional study | Receiving HD 3x per week, standard low-phosphate diet with protein content between 1-1.2 g/kg of body weight daily | HDF patients, cachexia, neoplasm disease, receiving oral bicarbonate | 4.3 (10.3) years | Dialysate bicarbonate mean 32.9 ± 1.8 mmol/L (individualized) | Pre- and post-dialysis serum bicarbonate, pH | 75 | 66 (16) |
| Kourtellidou, 2021 | UK | Randomized parallel-group trial | Adults ≥18 years receiving HD for ≥3 months, with 6-month average pre-dialysis serum bicarbonate level <22 mmol/L and potassium >4 mmol/L | Recurrent hospital admissions, dementia, or bedbound | Intervention 23 (10-51), control 38 (23-83) months* | Sodium bicarbonate started at 1 g twice a day with the dose titrated during the first 4 weeks of treatment (increasing by 0.5 g twice a day as tolerated, to a maximum of 3 g twice a day) to achieve pre-dialysis bicarbonate over 22 mmol/l (individualized). Dialysate concentration was fixed at 35 mmol/L (32 mmol/L bicarbonate and 3 mmol/L acetate). | Systolic blood pressure, diastolic blood pressure, PR interval, Qtc interval, nPCR, pre- and post-dialysis serum bicarbonate, potassium | 33 | Intervention 57 (49-63), control 58 (52-67)* |
| Hefzollah, 2020 | Iran | Randomized parallel-group trial | Adults >18 years receiving HD for at least 1 year | Kt/V <1.2, malignancy, infection, malabsorptive disease, active congestive heart failure or ischemic heart disease, pre-dialysis serum potassium <3.5 mmol/L or serum calcium <1.9 mmol/L, corticosteroid treatment | Not reported | 36 mmol/L dialysate bicarbonate for 6 months vs 30 mmol/L dialysate bicarbonate (fixed) | Albumin, BMI, pre-dialysis potassium, calcium, serum bicarbonate | 56 (26 intervention, 30 control) | Intervention 61 (11), control 57 (12) |
| Montagud-Marrahi, 2020 | Spain | Prospective cohort study | Patients ≥18 years receiving HDF for ≥12 months | Percutaneous catheter (not tunnelled) as vascular access, living kidney donor program, immunosuppressive treatment, residual diuresis >100mL/24h and urea clearance >2.5 mL/min/1.7 m^2^ | 60.6 (56.7) months | Dialysate bicarbonate decreased from 35 mmol/L to 32 mmol/L (fixed) | Albumin, pre- and post-dialysis plasma bicarbonate, calcium, potassium, PTH | 84 | 67 (15) |
| Valério Alves, 2020 | Portugal | Prospective cohort | All outpatients in HD unit | None specified | Not reported | Dialysate bicarbonate adjusted every 3 months (9 time points): dialysate bicarbonate >30 mmol/L. reduce 4 mmol/L; ≥25 mmol/L, reduce 2 mmol/L; 20 mmol/L – 25 mmol/L, no change; ≤20 mmol/L, increase 2 mmol/L; <18 mmol/L, increase 4 mmol/L (individualized) | Pre-dialysis serum bicarbonate, pH, iPTH, albumin, calcium | 31 | 74 (13) |
| Bozikas, 2019 | Greece | Non-randomized interventional study | Stable HD patient without any concurrent illness, patients with a pre-dialysis serum bicarbonate concentration <22 mmol/L went on to receive the study intervention | None specified | 59 (70) months | Dialysate bicarbonate 35 mmol/L (32 bicarbonate, 3 acetate) increased to 37 mmol/L for 2 weeks (period A) followed by switching back to a dialysate bicarbonate 35 mmol/L and adding 5g oral bicarbonate daily for 2 weeks (period B) (fixed) | Pre- and post-dialysis serum bicarbonate, ionized calcium, pH, potassium | 60 (25 patients met criteria to receive the intervention) | 72 (10) |
| Panesar, 2017 | USA | Prospective cohort study | Patients receiving HD with NaturaLyte dialysate solution | Oral sodium bicarbonate supplementation, active infection or antibiotic treatment, intra-dialytic hypotension (SBP≤100 mmHg) or pre-dialysis SBP of ≤100 mmHg | 52.0 (53.5) months | Dialysate bicarbonate 30-32 mmol/L, 33-34 mmol/L, 35-36 mmol/L, or 37-40 mmol/L (uncertain how dialysate bicarbonate concentration prescribing was determined) | Post-dialysis serum bicarbonate, pre-and post-dialysis calcium, potassium | 39 | 57 (11) |
| Sajgure, 2017 | India | Non-randomized interventional study | Receiving outpatient HD for >1 month | Acute illness | 18 (16.7) months | Dialysate bicarbonate 35 mmol/L (fixed), oral bicarbonate dosage targeting mid-week pre-dialysis serum bicarbonate ≥22 mmol/L (individualized) | Albumin, BMI, triceps skinfold thickness, mid-arm circumference, protein intake, pre-dialysis serum bicarbonate, pre-dialysis pH | 35 | 48 (14) |
| Viegas, 2017 | Portugal | Randomized parallel-group trial | Adults receiving HDF (>20 L/session) three times per week, and on HDF for at least three months | Received dialysate bicarbonate concentration different from 34 mmol/L in previous month, severe malnutrition (serum albumin <2.5 g/dL), hospitalization >3 weeks during study | Intervention 40.5 (51.0) months, control 24.0 (56.1) months* | 34 mmol/L dialysate bicarbonate vs 30 mmol/L dialysate bicarbonate (fixed) | Pre-and post-dialysis serum bicarbonate, intradialytic hypotension | 93 | Intervention 61 (33), control 72 (19)* |
| Sabzghabaei, 2016 | Iran | Randomized crossover trial | Patients >16 years receiving HD for 4 hours, three times per week for at least 6 months | Dysrhythmias, heart failure, active coronary disease, antiarrhythmic drugs | Not reported | Four solutions (2 with dialysate bicarbonate 24 mmol/L, 2 with dialysate bicarbonate 28 mmol/L, and all with varying potassium, calcium, sodium concentrations) (fixed) | Change in QTc interval on dialysis | 35 | 54 (15) |
| Voiculet, 2016 | Romania | Randomized parallel-group trial | CKD stage 5 undergoing chronic HD (>3 months), residual diuresis >500 mL/day, pre-dialysis BP <160 mmHg in last 3 months | Diabetes mellitus, severe valve disease, systemic vasculitis, active neoplasia, bone pathology before CKD onset | Intervention 55.5 (34.5) months, control 59.7 (34.8) months | Oral bicarbonate  5g/day on non-dialysis days and mean dialysate bicarbonate 29.8 (1.4) mmol/L in the intervention group, no oral sodium bicarbonate supplementation and mean dialysate bicarbonate 33 (2.2) mmol/L in the control group vs (uncertain how dialysate bicarbonate concentration prescribing was determined) | Pre-dialysis serum bicarbonate, calcium, iPTH, vascular calcification | 63 | Intervention group: 56 (13)  Control group: 57 (15) |
| Bales, 2015 | Brazil | Non-randomized interventional study | Receiving HD for ≥6 months | Infection 1 month prior to study entry | 118 (115)* months | Dialysate bicarbonate 32 mmol/L at baseline, adjusted individually to maintain pre-dialysis bicarbonate ≥22 mmol/L (individualized) | Systolic blood pressure, albumin, BMI, pre-dialysis serum bicarbonate, calcium, pH, PTH | 48 | 43 (19) |
| Silva, 2014 | Brazil | Cross-sectional study | Adult patients receiving HD for ≥6 months | Diabetes mellitus, non-sinus cardiac rhythm, recent parathyroidectomy (<6 months), history of congestive heart failure | 166 (108) months | Dialysate bicarbonate ≤38 mmol/L and >38 mmol/L (fixed) | Cardiac index, post-dialysis serum bicarbonate, pre-dialysis calcium | 30 | 40 (11) |
| Checherita, 2013 | Romania | Non-randomized interventional study  (pre-post) | Chronic HD or PD patients but only HD patients presented in the article | Life-threatening comorbidities (severe neurological disorders, neoplasia with metastasis or recent chemotherapy, severe heart, hepatic or respiratory failure) and those who did not consent | 54 months (no SD) | 650mg oral bicarbonate tablets (2, 4, or 6 tablets per day) on interdialytic days (fixed/individualized dialysate bicarbonate not specified) | Hospitalizations, duration of hospitalization, systolic blood pressure, diastolic blood pressure, intradialytic hypotension, pre-dialysis serum bicarbonate | 164 | 41 (no SD) |
| Tentori, 2013 | Australia, Belgium, Canada, France, Germany, Italy, New Zealand, Spain, Sweden, UK, US | Prospective cohort study | Adult HD patients ≥18 years, dialyzing 3 times per week, dialysate bicarbonate within 20–45 mmol/L, serum bicarbonate 10–36 mmol/L | Acetate predominant dialysate base, facilities with <5 eligible patients, facilities that measured serum bicarbonate in <60%; some US facilities with incomplete data | 3.6 (5.0) years | Continuous mean dialysate bicarbonate 35.5 ± 2.7 mmol/L; categorically ≤32 mmol/L, 33–37 mmol/L, or ≥38 mmol/L (includes fixed and individualized dialysate bicarbonate facilities) | All-cause mortality, cause-specific mortality, first hospitalization, intradialytic hypotension, pre-dialysis serum bicarbonate | 17,031 | 64 (15) |
| Di Iorio, 2012 | Italy | Randomized crossover trial | HD for at least 6 months  Stable post-dialytic dry weight | Arrhythmias, use of antiarrhythmic drugs in the previous 3 months Frequent intradialytic hypotension, acute illness, past ischemic heart disease, atrial fibrillation, low ejection fraction, treatment with drugs affecting the QT | 62.7 (47.6) months | Dialysate bicarbonate 30 mmol/L and 34 mmol/L (fixed) | QTc interval measured during dialysis and hourly for 4 hours after the end of dialysis, post-dialysis serum bicarbonate, calcium, potassium, pH | 22 | 66 (11) |
| Gabutti, 2009 | Switzerland | Randomized crossover trial | Dialyzed for 4 hours, three times weekly, stable, no intercurrent illnesses | None specified | Not reported | Dialysate bicarbonate 26-29 mmol/L and 32-35 mmol/L (Appears somewhat individualized)  In one arm, patients with a baseline bicarbonate ≥30 mmol/L were dialyzed reducing the concentration by 6 mmol/L while patients initially treated with a bicarbonate <30 mmol/L were dialyzed increasing the concentration by 6 mmol/L; in the other arm, the ionized calcium concentration was changed from 1.3 to 1.5 mmol/L or vice versa | Systolic blood pressure, diastolic blood pressure, pre- and post-dialysis calcium, potassium, pH, stroke volume, peripheral resistance | 21 | 69 (10) |
| Movilli, 2009 | Italy | Non-randomized interventional study | Regular HD treatment for ≥6 months, serum bicarbonate concentrations ≤20 mmol/L in at least three consecutive pre-HD measurements before study, no acute infective/inflammatory disease for ≥ 4 weeks before study | Diabetes mellitus, neoplasia, active liver disease, cachexia | 30 (10-366) months* | Oral bicarbonate  mean dose 2.9 ± 0.9 g/day (range 1-4 g/day); target pre-dialysis serum bicarbonate between 23-26 mmol/L (individualized dosing of oral bicarbonate with fixed dialysate bicarbonate of 35 mmol/L and acetate 4 mmol/L) | Systolic blood pressure, diastolic blood pressure, albumin, nPCR, pre-dialysis serum bicarbonate, pH | 29 | 61 (14) years |
| Bossola, 2007 | Italy | Non-randomized interventional study | Venous bicarbonate <19 mmol/L on two consecutive midweek pre-dialysis measurements  Stable clinical condition and HD regimen for at least 12 months | Cancer, sepsis  AIDS, inflammatory bowel disease, autoimmune disorders, congestive heart failure, acute and chronic hepatitis, liver failure, hyperthyroidism, COPD | 84.9 (81.7) months | 1g oral bicarbonate, thrice daily, for 12 months (fixed oral sodium bicarbonate and fixed dialysate bicarbonate 35 mmol/L) | Albumin, nPCR, Subjective Global Assessment (SGA), pre-dialysis serum bicarbonate | 20 | 63 (6) |
| Noh, 2007 | South Korea | Prospective cohort study | Stable patients receiving HD for 4 hours, three times weekly, for >3 months | Factors which may influence blood pH (e.g., acute infections, respiratory diseases, oral bicarbonate supplementation, sevelamer as a phosphate binder) | 64.4 (47.1) months | Dialysate 25 mmol/L, 30 mmol/L, or 35 mmol/L (uncertain how dialysate bicarbonate concentration prescribing was determined) | Pre- and post-dialysis serum bicarbonate and pH | 53 | 56 (13) |
| Gabutti, 2005 | Switzerland | Randomized crossover trial | Chronic HD patients who were clinically stable and without intercurrent illnesses | None specified | Not reported | Dialysate bicarbonate 32 mmol/L and 26 mmol/L (fixed) | Systolic blood pressure, diastolic blood pressure, pre- and post-dialysis calcium, potassium, pH | 27 | 66 (9) |
| Movilli, 2005 | Italy | Cross sectional study followed by a non-randomized interventional study in 18 acidotic patients (bicarbonate **≤**20 mmol/L) not taking oral sodium bicarbonate at baseline with 4 month follow up | Regular HD treatment for at ≥6 months before study, no clinical evidence of infective/inflammatory disease for ≥ 4 weeks before study | Residual urine output 6150 mL/day, active neoplasia, cachexia | 48 (6-372) months* | Cross sectional study: Oral bicarbonate, mean dose 1.9 (0.9) g per day (range 1-5, median 2 g per day), (unclear how dose determined) vs no oral bicarbonate supplementation (fixed dialysate bicarbonate 35 mmol/L, acetate 4 mmol/L) Interventional study: oral sodium bicarbonate started for acidotic patients not on oral bicarbonate at baseline (mean dose 2.6 g/day,  range 1–4; pre-dialysis serum bicarbonate target 23-25 mmol/L; individualized) | Systolic blood pressure, diastolic blood pressure, pre-dialysis pH, BMI, nPCR | Cross sectional study: 110  (70 on regular oral bicarbonate, 40 no supplementation)  Interventional study: 18 patients given oral sodium bicarbonate | 67 (15) |
| Blair, 2003 | USA | Prospective cohort study | On maintenance HD for at least 1 year on the standard bicarbonate dialysate of 35 mmol/L without oral bicarbonate supplements | Not described for patients  Samples exceeding the standard limit of  20% for technical variance were not included | 44.7 (31.2) months | Dialysate bicarbonate increased from 35 to 39 mmol/L (fixed) | Albumin, SGA, pre-dialysis serum bicarbonate, calcium, potassium, PTH | 199 | 62 (15) |
| Gabutti, 2003 | Switzerland | Randomized crossover trial | Clinically stable HD patients without intercurrent illnesses | None specified | Not reported | Dialysate bicarbonate 32 mmol/L and 26 mmol/L (fixed) | Systolic blood pressure, diastolic blood pressure, intradialytic hypotension, pre-dialysis serum bicarbonate, pre- and post-dialysis calcium, potassium, pH | 26 | 68 (11) |
| Lin, 2002 | Taiwan | Non-randomized interventional study | Virtually anuric (<50 mL per day and 82% anuria) patients receiving HD for ≥6 months | Infection, liver cirrhosis, heart failure, active issues requiring hospitalization,  elevated blood lactate or ketoacid anions | Group A 49 (11),  group B 36 (5),  group C 39 (3) months | Dialysate bicarbonate 35 mmol/L with acetate 4 mmol/L at baseline. Patients in Group A were found to have a pre-dialysis serum bicarbonate <22 mmol/L. Patients in this group had their dialysate bicarbonate increased to 38 mmol/L for the first month, then 38-40 mmol/L, adjusted to achieve a pre-dialysis serum bicarbonate 23-26 mmol/L (individualized) | Mean arterial pressure, albumin, BMI, triceps skinfold thickness, protein intake, nPCR, pre-dialysis serum bicarbonate, potassium, pH, PTH | 120, the intervention was only applied to patients in Group A (n=21, 17 completed the intervention) | Group A 50 (3), group B 59 (2), group C 64 (2) |
| Brady, 1998 | USA | Randomized parallel-group trial | Adults with life expectancy ≥4 months, dialysate bicarbonate 35 mmol/L, mean pre-dialysis serum bicarbonate **≤**18 mmol/L in 3 months before study | Receiving supplemental oral sodium bicarbonate/citrate before study, pregnant or childbearing potential, participating in another interventional research study | Intervention 62.3 (61.0) months, control 57.9 (57.4) months | 40 mmol/L dialysate bicarbonate, plus 1 mmol/kg/d oral sodium bicarbonate if pre-dialysis serum bicarbonate <22 mmol/L after 2 weeks vs 35 mmol/L dialysate bicarbonate (fixed dialysate bicarbonate) | Serum albumin, nPCR, pre-dialysis serum bicarbonate, calcium, potassium, PTH | 36 (18 intervention, 18 control) | Intervention 51 (15), control 52 (16) |
| Williams, 1997 | UK | Randomized crossover trial | Stable, receiving thrice weekly HD for ≥6 months using dialysate bicarbonate 35 mmol/L | None specified | Not reported | Dialysate bicarbonate 40 mmol/L vs dialysate bicarbonate 30 mmol/L (fixed) | Albumin, triceps skinfold thickness, nPCR, pre-and post-dialysis calcium, potassium, pH, PTH | 46 | 64 (14) |
| Oettinger, 1993 | USA | Non-randomized interventional study | None specified | None specified | Not reported | Dialysate bicarbonate 39 mmol/L vs 36 mmol/L (fixed) | Pre- and post-dialysis serum bicarbonate, calcium | 38 | Not specified |
| Caruana, 1989 | USA | Non-randomized interventional study | Recent/previous low serum bicarbonate levels and being actively treated with oral base supplements | Underwent subtotal parathyroidectomy, prior poor compliance with medical regimens | 58.7 (45) months | Oral sodium bicarbonate supplementation adjusted per mid-week pre-dialysis serum bicarbonate with a target of 22-26 mmol/L (4 patients on fixed dialysate bicarbonate 39 mmol/L and acetate 4 mmol/L, 16 on acetate dialysate 37 mmol/L) | Albumin, pre-dialysis serum bicarbonate, pre- and post-dialysis calcium, pH, PTH | 20 | 46 (14) |
| Lefebvre, 1989 | France | Randomized parallel-group trial | Adult HD patients | Severe radiological osteitis fibrosa, plasma calcium >2.8mmol/L, parathyroidectomy, diabetes mellitus, corticosteroid treatment | Intervention 6.6 (3.3) years, control 6.2 (3.0) years | 7-15 mmol/L bicarbonate added to dialysate to obtain pre-dialysis serum bicarbonate 24 mmol/L (individualized) vs 33 (2) mmol/L dialysate bicarbonate (fixed value determined by pre-study dialysate bicarbonate concentration) | Pre- and post-dialysis serum bicarbonate, calcium, potassium, pre-dialysis pH, PTH | 21 (11 acidosis corrected, 10 without correction) | Acidosis correction group: 51 (9), non-correction group: 50 (14) |

Abbreviations: BMI= Body mass index, CKD= Chronic kidney disease, COPD= Chronic obstructive pulmonary disease, HD= Hemodialysis, HDF= Hemodiafiltration, PTH= parathyroid hormone, nPCR= Normalized protein catabolic rate, nPNA=

Conversion factors: Calcium conventional unit (mg/dL) to SI unit (mmol/L) = 0.25

Normalized protein nitrogen appearance, PD= Peritoneal dialysis, SBP= Systolic blood pressure, SGA= Subjective Global Assessment

Data presented as mean (SD) unless otherwise indicated: *median (IQR)

Values rounded to one decimal place or one significant figure

¶Age rounded to nearest whole number

**Supplemental Table 2. Characteristics of PD studies**

| Study, year | Country | Study Design | Inclusion Criteria | Exclusion Criteria | Dialysis vintage | Intervention/Exposure and Comparator | Outcomes | Number of patients | Age of participants in years |
| --- | --- | --- | --- | --- | --- | --- | --- | --- | --- |
| Liu, 2017 | China | Randomized parallel-group trial | Adults receiving CAPD for ≥ 6 months, serum bicarbonate <24 mmol/L for ≥2 consecutive visits | <18 years at the initiation of PD, history of HD/kidney transplantation, acute kidney injury, 24-hour urine output <200 mL/day, oral bicarbonate within 2 weeks before enrolment, kidney transplant/ transfer to other renal centre within 6 months | 35.6 (20.2) months intervention, 44.6 (24.7) months placebo | 1g/day oral sodium bicarbonate, with up-titration if bicarbonate <24 mmol/L or down-titration if bicarbonate >30 mmol/L vs placebo | Calcium, potassium | 40 | Placebo 56 (14), intervention 55 (16) |
| Szeto, 2003 | China | Randomized parallel-group trial | Total weekly Kt/V <2.1, venous bicarbonate 25 mmol/L on two consecutive measurements, stable, CAPD for ≥12 months | Unlikely survival, possible kidney transplant to other renal centre within 6 months | 39.9 (20.8) months intervention, 39.4 (26.0) months placebo | 0.9g oral sodium bicarbonate 3x daily for 12 months vs placebo | All-cause mortality, hospitalizations, duration of hospitalization, albumin, lean body mass, nPNA, SGA | 60 | Placebo 57 (13),  intervention 54 (12) |
| Feriani, 2004 | Germany | Non-randomized interventional study | Adults ≥18 years receiving CAPD for ≥3 months, no peritonitis for at ≥4 weeks prior to study | Pulmonary dysfunction, malignancies, weekly total Kt/V <1.7, receiving oral bicarbonate supplementation | 19 (3-47) (34 mmol/L group), 12 (3-55) (39 mmol/L group) months† | Patients with a serum bicarbonate <25.3 mmol/L were assigned to 39 mmol/L dialysate bicarbonate vs patients with a serum bicarbonate >25.3 mmol/L were assigned to 34 mmol/L dialysate bicarbonate | Protein nitrogen appearance, serum bicarbonate, pH | 47 | Bic 34 group: 65 (31-74)  Bic 39 group: 57 (23-75)† |
| Stein, 1997 | UK | Randomized parallel-group trial | Patients treated by CAPD from the start of renal replacement therapy | None specified | 1 month | Low alkali (lactate 35 mmol/L) vs high alkali (lactate 40 mmol/L + optional oral sodium bicarbonate averaging 1.4 (0.2) g/day) targeting serum bicarbonate 30 mmol/L | Albumin, triceps skin fold thickness, midarm circumference, protein catabolic rate, all-cause mortality, cardiovascular mortality, hospitalization, systolic blood pressure, diastolic blood pressure, calcium, potassium, PTH, serum bicarbonate, pH | 200 | Low alkali 57 (2), high alkali 56 (1) |

Abbreviations: CAPD= Continuous ambulatory peritoneal dialysis, HD= Hemodialysis, PD= Peritoneal dialysis, PTH= Parathyroid hormone, nPNA= Normalized protein nitrogen appearance, SGA= Subjective Global Assessment

Conversion factors: Lactate conventional unit (mg/dL) to SI unit (mmol/L) = 0.111

Data presented as mean (SD) unless otherwise indicated: †median (range)

Values rounded to one decimal place or one significant figure

¶Age rounded to nearest whole number

**Supplemental Table 3. Quality of observational cohort studies**

| Study, year | Selection | Comparability | Outcome | Overall quality* |
| --- | --- | --- | --- | --- |
| Molnar, 2024 | *** | ** | *** | Good |
| Ravi, 2024 | *** | ** | *** | Good |
| Law and Davenport, 2023 | *** | ** | *** | Good |
| Wan, 2023 | *** | ** | *** | Good |
| Cuadrado, 2022 | ** | ** | *** | Fair |
| Montagud-Marrahi, 2020 | ** | ** | *** | Fair |
| Valério Alves, 2020 | *** | 0 | 0 | Poor |
| Panesar, 2017 | *** | 0 | ** | Poor |
| Tentori, 2013 | *** | ** | *** | Good |
| Noh, 2007 | ** | 0 | ** | Poor |
| Blair, 2003 | *** | 0 | *** | Poor |

*Thresholds for converting the Newcastle-Ottawa scales to AHRQ standards (good, fair, and poor):

Good quality: 3 or 4 stars in selection domain AND 1 or 2 stars in comparability domain AND 2 or 3 stars in outcome/exposure domain

Fair quality: 2 stars in selection domain AND 1 or 2 stars in comparability domain AND 2 or 3 stars in outcome/exposure domain

Poor quality: 0 or 1 star in selection domain OR 0 stars in comparability domain OR 0 or 1 stars in outcome/exposure domain

**Supplemental Table 4. Risk of bias of cross-sectional studies**

| Study, year | Inclusion criteria clearly defined | Subject/setting described in detail | Exposure measurement valid/reliable | Objective/standard criteria used for measurement of condition | Confounding factor identified | Strategies for confounding stated | Outcome measurement valid/reliable | Appropriate statistical analysis | Overall appraisal* |
| --- | --- | --- | --- | --- | --- | --- | --- | --- | --- |
| Wieliczko, 2022 | Y | Y | Y | Y | N | N | Y | Y | Moderate |
| Silva, 2014 | Y | Y | Y | Y | N | N | Y | Y | Moderate |
| Movilli, 2005 | Y | Y | Y | Y | N | N | Y | Y | Moderate |

*Overall appraisal criteria: Low risk of bias (0 No/Unclear), Moderate risk of bias (1-2 No/Unclear), High risk of bias (>2 No/Unclear)

**Supplemental Table 5. Cardiovascular outcomes in PD studies**

| **Outcome** | **Study design** | **Study, year** | **Intervention** | **Follow up time** | **Number of patients** | **Results** |
| --- | --- | --- | --- | --- | --- | --- |
| Systolic blood pressure  (SBP) | Randomized parallel-group trial | Liu, 2017 | Oral bicarbonate 1.0 g/day; up-titrated if serum bicarbonate <24, down-titrated if >30 mmol/L | 104 weeks | 28 (15 treatment, 13 placebo) | - Mean SBP 135 ± 18 mmHg at baseline vs 135 ± 18 mmHg at week 104 in treatment group. - Mean SBP 137 ± 19 mmHg at baseline vs 137 ± 19 mmHg at week 104 in placebo group. |
|  |  | Szeto, 2003 | Oral bicarbonate 0.9g 3x/day | 12 months | 60 (30 treatment, 30 placebo) | - Average SBP 144 mmHg in treatment group vs 143 mmHg in control group throughout the study (p=0.8). |
|  |  | Stein, 1997 | Low alkali (lactate 35 mmol/L) vs high alkali (lactate 40 mmol/L + optional oral sodium bicarbonate averaging 1.4 (0.2) g/day) targeting serum bicarbonate 30 mmol/L | 12 months | 200 (100 low alkali, 100 high alkali; 48 of high alkali receiving sodium bicarbonate) | - 130 ± 2 mmHg in high alkali group vs 129 ± 3 mmHg in low alkali group at one year (p>0.05). |
| Diastolic blood pressure (DBP) | Randomized parallel-group trial | Liu, 2017 | Oral bicarbonate 1.0 g/day; up-titrated if serum bicarbonate <24, down-titrated if >30 mmol/L | 104 weeks | 28 (15 treatment, 13 placebo) | - Mean DBP 82 ± 9 mmHg at baseline vs 84 ± 9 mmHg at week 104 in treatment group. - Mean DBP 81 ± 9 mmHg at baseline vs 83 ± 8 mmHg at week 104 in placebo group. |
|  |  | Szeto, 2003 | Oral bicarbonate 0.9g 3x/day | 12 months | 60 (30 treatment, 30 placebo) | - Average DBP 78 mmHg in treatment group vs 81 mmHg in control group throughout the study (p=0.8). |
|  |  | Stein, 1997 | Low alkali (lactate 35 mmol/L) vs high alkali (lactate 40 mmol/L + optional oral sodium bicarbonate averaging 1.4 (0.2) g/day) targeting serum bicarbonate 30 mmol/L | 12 months | 200 (100 low alkali, 100 high alkali; 48 of high alkali receiving sodium bicarbonate) | - 78 ± 1 mmHg in high alkali group vs 76 ± 1 mmHg in low alkali group at one year (p>0.05). |

**Supplemental Table 6. Nutritional outcomes in HD/HDF studies**

| **Outcome** | **Study design** | **Study, year** | **Intervention** | **Follow up time** | **Number of patients** | **Results** |
| --- | --- | --- | --- | --- | --- | --- |
| Serum albumin | Randomized parallel-group trial | Rasheed, 2023 | Oral sodium bicarbonate (500 mg) daily | 3 months | 50 (25 intervention, 25 control) | - 4.2 ± 0.5 g/L in intervention group vs 3.8 ± 0.5 g/L in control group (p=0.01) at 3 months. |
|  |  | Hefzollah, 2020 | Dialysate bicarbonate 36 vs 30 mmol/L | 6 months | 56 (26: 36 mmol/L, 30: 30 mmol/L) | - 44 ± 3.0 g/L at baseline to 43 ± 4.0 g/L at 6 months (p=0.7) in control group (bicarbonate 30). - 40 ± 2.0 g/L at baseline to 43 ± 6.0 g/L at 6 months (p=0.2) in intervention group (bicarbonate 36). |
|  |  | Brady, 1998 | Dialysate bicarbonate 40 vs 35 mmol/L | 16 weeks | 36 (18: 40 mmol/L, 18: 35 mmol/L) | - 39 ± 3.0 g/L (35 mmol/L) vs 38 ± 3.0 g/L in (40 mmol/L) (p>0.05). |
|  | Randomized crossover trial | Williams, 1997 | Group A (dialysate bicarbonate 30 to 40 mmol/L), Group B (40 mmol/L to 30 mmol/L), and standard bicarbonate 35 mmol/L | 12 months (crossover at 6 months) | 46 | - 39.4 ± 3.5 g/L at baseline vs 40.9 ± 3.3 g/L at 12 months in Group A (30 mmol/L to 40 mmol/L). - 40.3 ± 4.1 g/L at baseline vs 40.6 ± 4.5 g/L at 12 months in Group B (40 mmol/L to 30 mmol/L). |
|  | Non-randomized intervention | Sajgure, 2017 | Oral bicarbonate mean dose 0.7 ± 0.4 mmol/kg/day at baseline to 1.0 ± 0.6 mmol/kg/day; dosage increased if baseline serum bicarbonate <22 mmol/L | 3 months | 35 | - 39 ± 5.0 at baseline vs 42 ± 4.0 g/L at month 3 (p<0.001). |
|  |  | Bales, 2015 | Dialysate bicarbonate baseline 32 mmol/L, adjusted to maintain pre-dialysis serum bicarbonate ≥22 mmol/L | 4 months | 48 | - 35 ± 3.0 at month 0 vs 40 ± 3.0 g/L at month 4 (p<0.001). |
|  |  | Movilli, 2009 | Oral bicarbonate mean dose 2.9 ± 0.9 g/day; pre-dialysis serum bicarbonate maintained between 23-26 mmol/L | 30 months | 29 | - 36 ± 2.0 g/L at baseline vs 37 ± 3.0 g/L at follow-up (p>0.05). |
|  |  | Bossola, 2007 | Oral bicarbonate 1g 3x/day for 12 months | 12 months | 20 | - No significant difference between baseline (38 ± 2.0 g/L) and 12 months (39 ± 2.0 g/L) (p=0.3). |
|  |  | Lin, 2002 | Dialysate bicarbonate increased from 35 to 38 mmol/L (month 1), then adjusted to 38-40 mmol/L in subgroup A (patients with pre-dialysis serum bicarbonate ≤21 mmol/L) | 6 months | 17 | - 43.4 ± 0.8 g/L at baseline vs 43.8 ± 0.8 g/L at 6 months (p>0.05) in subgroup with pre-dialysis bicarbonate ≤21 mmol/L. |
|  |  | Caruana, 1989 | Oral bicarbonate adjusted targeting mid-week pre-dialysis bicarbonate 22-26 mmol/L | 1 month | 20 | - 40 ± 3.0 g/L at baseline vs 40 ± 3.0 g/L at follow-up (p>0.05). |
|  | Prospective cohort | Law and Davenport, 2023 | Dialysate bicarbonate 32 mmol/L at baseline, switched to 28 mmol/L at study start | 19 months | 126 | - Pre-HD albumin 39.7 ± 4.2 g/L at baseline (dialysate bicarbonate 32 mmol/L) and 37.6 ± 4.0 g/L at study end (dialysate 28 mmol/L), p<0.001. |
|  |  | Montagud-Marrahi, 2020 | Dialysate bicarbonate 35 mmol/L at baseline switched to 32 mmol/L at study start | 6 months | 84 | - 38.9 ± 3.2 g/L at baseline vs 37.9 ± 3.1 g/l at 3 months (p<0.05 vs baseline) vs 38.4 ± 3.7 g/L at 6 months. |
|  |  | Valério Alves, 2020 | Dialysate bicarbonate adjusted every 3 months (9 time points): dialysate bicarbonate >30 mmol/L. reduce 4 mmol/L; ≥25 mmol/L, reduce 2 mmol/L; 20 mmol/L – 25 mmol/L, no change; ≤20 mmol/L, increase 2 mmol/L; <18 mmol/L, increase 4 mmol/L (individualized) | 24 months | 31 | - Mean (SD) 33 ± 3.0 g/L at baseline (mean dialysate bicarbonate 32 mmol/L) to median (IQR) 35 (5.0) g/L at 24 months (mean dialysate bicarbonate 28 mmol/L). |
|  |  | Blair, 2003 | Dialysate bicarbonate baseline 35 mmol/L increased to 39 mmol/L | 6 months | 198-199 | - 39 ± 4.0 (n=198) at baseline vs 38 ± 4.0 g/L at 3 months (n=199) (p<0.05), vs 39 ± 4.0 (n=199) at 6 months. |
|  | Retrospective cohort | Molnar, 2024 | Standardized dialysate bicarbonate (35, 36-37, and ≥38 mmol/L) and individualized dialysate bicarbonate concentration | 1 year | 5414 | - 37.5 (4.9), 37.0 (5.0), 36.9 (4.9), 32.8 (4.3), and 39.0 (4.6) g/L in individualized group, combined standardized group all together, 35 mmol/L group, 36-37 mmol/L group, and ≥38 mmol/L group, respectively. |
|  | Cross-sectional | Wieliczko, 2022 | Dialysate bicarbonate mean 32.9 ± 1.8 mmol/L (range 28-36 mmol/L), titrated depending on pre-dialysis serum bicarbonate | Dialysis session | 75 | - Correlation between bicarbonate bath and pre-HD serum albumin= 0.3, p= 0.01. |
| BMI | Randomized parallel-group trial | Hefzollah, 2020 | Dialysate bicarbonate 36 vs 30 mmol/L | 6 months | 56 (26 intervention, 30 control) | - 24.4 ± 4.6 kg/m2, 0.2 in control group (bicarbonate 30) vs 26.2 ± 3.8 kg/m2 in intervention group (bicarbonate 36) (p=0.001). |
|  | Non-randomized intervention | Sajgure, 2017 | Oral bicarbonate mean dose 0.7 ± 0.4 mmol/kg/day at baseline to 1.0 ± 0.6 mmol/kg/day; dosage increased if baseline serum bicarbonate <22 mmol/L | 3 months | 35 | - 18.8 ± 3.0 kg/m2 at baseline vs 19.1 ± 3.0 kg/m2 at month 3 (p<0.001). |
|  |  | Bales, 2015 | Dialysate bicarbonate baseline 32 mmol/L, adjusted to maintain pre-dialysis serum bicarbonate ≥22 mmol/L | 4 months | 48 | - Non-significant change from month 0 (22.6 ± 3.6 kg/m2) to month 4 (22.6 ± 3.7 kg/m2) (p=0.9). |
|  |  | Lin, 2002 | Dialysate bicarbonate increased from 35 to 38 mmol/L (month 1), then adjusted to 38-40 mmol/L in subgroup A (patients with pre-dialysis serum bicarbonate ≤21 mmol/L) | 6 months | 17 | - 23.1 ± 1.0 kg/m2 at baseline vs 23.1 ± 1.0 kg/m2 at 6 months (p>0.05). |
|  | Cross-sectional | Movilli, 2005 | Dialysate bicarbonate 35 mmol/L; Group A daily oral sodium bicarbonate administration mean dose 1.9 ± 0.9 g/day (range 1–5 g/day), Group B control | N/A | 110 (70 intervention) | - 25 ± 5 kg/m2 in intervention group vs 24 ± 5 kg/m2 vs control (p>0.05). |
| Hand grip strength | Randomized parallel-group trial | Rasheed, 2023 | Oral sodium bicarbonate (500 mg) daily | 3 months | 50 (25 intervention, 25 control) | - 45.0 ± 19.2 kg in intervention group vs 33.9 ± 15.1 kg in control group (p<0.05) at 3 months. |
|  |  | Kourtellidou, 2021 | Average 3.7 ± 0.5 g oral sodium bicarbonate (maximum 6 g) daily | 20 weeks | 33 (15 intervention, 18 control) | - 26.6 ± 13.1 kg at baseline to 26.2 ± 13.4 kg at study end in intervention group. - 31.2 ± 10.9 kg at baseline to 29.7 ± 10.1 kg at study end in control group. - Mean change during study −0.4 ± 3.4 kg in intervention group; −1.5 ± 3.0 kg in control group (p=0.3). |
| Lean tissue index | Prospective cohort | Law and Davenport, 2023 | Dialysate bicarbonate 28 mmol/L versus 32 mmol/L | 19 months | 126 | - Lean tissue index 17.8 ± 4.5 kg/m^2^ at baseline (dialysate bicarbonate 32 mmol/L) and 17.6 ± 3.9 kg/m^2^ at study end (dialysate 28 mmol/L), p>0.05. |
| Lean tissue mass | Randomized parallel-group trial | Kourtellidou, 2021 | Average 3.7 ± 0.5 g oral sodium bicarbonate (maximum 6 g) daily | 20 weeks | 33 (15 intervention, 18 control) | - 45.0 ± 12.2% at baseline to 45.5 ± 12.5% at study end in intervention group. - 51.8 ± 13.7% at baseline to 50.2 ± 12.1% at study end in control group. - Mean change during study + 0.5 ± 3.3% in intervention group; −1.6 ± 2.9% in control group (p=0.07). |
| Triceps skinfold thickness | Randomized crossover trial | Rasheed, 2023 | Oral sodium bicarbonate (500 mg) daily | 3 months | 50 (25 intervention, 25 control) | - 10.3 ± 4.9 mm in intervention group vs 10.5 ± 8.0 in control group (p=0.9) at 3 months. |
|  |  | Williams, 1997 | Group A (dialysate bicarbonate 30 to 40 mmol/L), Group B (40 mmol/L to 30 mmol/L), and standard bicarbonate 35 mmol/L (two consecutive 6-month periods for crossover) | 12 months (crossover at 6 months) | 46 | - 14.8 ± 6.9 mm at baseline, 11.8 ± 5.5 mm at 6 months (p<0.05 vs baseline), 13.3 ± 7.2 mm at 12 months (p<0.05 vs 6 months) in Group A (30 mmol/L to 40 mmol/L). - 14.9 ± 6.3 mm at baseline, 15.8 ± 6.4 mm at 6 months (p<0.05 vs baseline), 13.8 ± 6.7 mm at 12 months (p<0.05 vs 6 months) in Group B (40 mmol/L to 30 mmol/L). |
|  | Non-randomized intervention | Sajgure, 2017 | Oral bicarbonate mean dose 0.7 ± 0.4 mmol/kg/day at baseline to 1.0 ± 0.6 mmol/kg/day; dosage increased if baseline serum bicarbonate <22 mmol/L | 3 months | 35 | - 1.1 ± 0.4 cm at baseline vs 0.9 ± 0.3 cm at 3 months (p<0.05). |
|  |  | Lin, 2002 | Dialysate bicarbonate increased from 35 to 38 mmol/L (month 1), then adjusted to 38-40 mmol/L in subgroup A (patients with pre-dialysis serum bicarbonate ≤21 mmol/L) | 6 months | 17 | - 23.5 ± 1.0 mm at baseline vs 23.8 ± 1.1 mm at 6 months in subgroup with pre-dialysis bicarbonate ≤21 mmol/L. |
| Mid-arm circumference | Non-randomized intervention | Sajgure, 2017 | Oral bicarbonate mean dose 0.7 ± 0.4 mmol/kg/day at baseline to 1.0 ± 0.6 mmol/kg/day; dosage increased if baseline serum bicarbonate <22 mmol/L | 3 months | 35 | - 22.6 ± 4.8 cm at baseline vs 23.9 ± 3.8 cm at 3 months (p<0.01). |
| nPCR | Randomized parallel-group trial | Rasheed, 2023 | Oral sodium bicarbonate (500 mg) daily | 3 months | 50 (25 intervention, 25 control) | - 1.06 + 0.27 g/kg/day in intervention group vs 0.90 + 0.27 g/kg/day in control group (p<0.05). |
|  |  | Kourtellidou, 2021 | Average 3.7 ± 0.5 g oral sodium bicarbonate (maximum 6 g) daily | 20 weeks | 33 (15 intervention, 18 control) | - 1.1 ± 0.3 at baseline to 1.1 ± 0.2 g/kg/day in intervention group. - 1.1 ± 0.2 at baseline to 1.1 ± 0.2 g/kg/day in control group. - Mean change during study +0.02 ± 0.2 g/kg/day in intervention group; 0.1 ± 0.2 g/kg/day in control group (p=0.7). |
|  |  | Brady, 1998 | Dialysate bicarbonate 40 vs 35 mmol/L | 16 weeks | 38 (18 intervention, 18 control) | - 1.0 ± 0.2 g/kg/day in control group (bicarbonate 35) vs 0.9 ± 0.2 g/kg/day in intervention group (bicarbonate 40) (p>0.05). |
|  | Randomized crossover trial | Williams, 1997 | Group A (dialysate bicarbonate 30 to 40 mmol/L), Group B (40 mmol/L to 30 mmol/L), and standard bicarbonate 35 mmol/L (two consecutive 6-month periods for crossover) | 12 months (crossover at 6 months) | 46 | - 1.0 ± 0.3 g/kg/day with low bicarbonate (bicarbonate 30) vs 1.0 ± 0.3 g/kg/day with high bicarbonate (bicarbonate 40) (p>0.05). |
|  | Non-randomized intervention | Movilli, 2009 | Oral bicarbonate mean dose 2.9 ± 0.9 g/day; pre-dialysis serum bicarbonate maintained between 23-26 mmol/L | 30 months | 29 | - 1.1 ± 0.1 g/kg/day at baseline, 1.1 ± 0.1 g/kg/day at follow-up (p<0.001). |
|  |  | Bossola, 2007 | Oral bicarbonate 1g 3x/day for 12 months | 12 months | 20 | - 0.9 ± 0.1 g/kg/day to 0.9 ± 0.1 g/kg/day. |
|  |  | Lin, 2002 | Dialysate bicarbonate increased from 35 to 38 mmol/L (month 1), then adjusted to 38-40 mmol/L in subgroup A (patients with pre-dialysis serum bicarbonate ≤21 mmol/L) | 6 months | 17 | - 1.3 ± 0.03 g/kg/day at baseline vs 1.3 ± 0.03 g/kg/day at 6 months in Group A. |
|  | Cross-sectional | Movilli, 2005 | Dialysate bicarbonate 35 mmol/L; Group A daily oral sodium bicarbonate administration mean dose 1.9 ± 0.9 g/day (range 1–5 g/day), Group B control | N/A | 110 (70 intervention) | - 1.1 ± 0.2 (intervention) 1.1 ± 0.2 g/kg/day (control), p>0.05. |
| nPNA | Prospective cohort | Law and Davenport, 2023 | Dialysate bicarbonate 28 mmol/L versus 32 mmol/L | 19 months | 126 | - Pre-HD nPNA 0.8 ± 0.2 g/kg/d at baseline (dialysate bicarbonate 32 mmol/L) and 0.8 ± 0.2 g/kg/day at study end (dialysate 28 mmol/L), p>0.05. |
| Protein intake | Randomized parallel-group trial | Kourtellidou, 2021 | Average 3.7 ± 0.5 g oral sodium bicarbonate (maximum 6 g) daily | 20 weeks | 33 (15 intervention, 18 control) | - 0.9 ± 0.4 g/kg/day at baseline to 1.0 ± 0.4 g/kg/day at study end in intervention group. - 0.9 ± 0.4 g/kg/day at baseline to 0.9 ± 0.3 g/kg/day at study end in control group. - Mean change during study +0.02 ± 0.1 g/kg/day in intervention group; −0.01 ± 0.3 g/kg/day in control group (p=0.8). |
|  | Non-randomized intervention | Sajgure, 2017 | Oral bicarbonate mean dose 0.7 ± 0.4 mmol/kg/day at baseline to 1.0 ± 0.6 mmol/kg/day; dosage increased if baseline serum bicarbonate <22 mmol/L | 3 months | 35 | - 0.4 ± 0.1 g/k/g/day at baseline vs 0.4 ± 0.1 g/kg/day at 3 months (p<0.005). |
|  |  | Lin, 2002 | Dialysate bicarbonate increased from 35 to 38 mmol/L (month 1), then adjusted to 38-40 mmol/L in subgroup A (patients with pre-dialysis serum bicarbonate ≤21 mmol/L) | 6 months | 17 | - 1.3 ± 0.03 g/kg/day at baseline vs 1.2 ± 0.03 g/kg/day at 6 months in subgroup with pre-dialysis bicarbonate ≤21 mmol/L. |
| SGA | Non-randomized intervention | Bossola, 2007 | Oral bicarbonate 1g 3x/day for 12 months | 12 months | 20 | - 4.5 ± 0.4 at baseline to 4.6 ± 0.5 (p=0.1). |
|  | Prospective cohort | Blair, 2003 | Dialysate bicarbonate baseline 35 mmol/L increased to 39 mmol/L | 6 months | 35 | - 5.6 ± 1.1 at baseline vs 5.5 ± 1.3 at 6 months (p>0.05). |

Abbreviations: BMI= Body mass index, HD= Hemodialysis, nPCR= Normalized protein catabolic rate, nPNA= Normalized protein nitrogen appearance, SGA= Subjective Global Assessment

Conversion factors: Albumin conventional unit (g/dL) to SI unit (g/L) = 10

Values rounded to one decimal place or one significant figure

**Supplemental Table 7. Nutritional outcomes in PD studies**

| **Outcome** | **Study design** | **Study, year** | **Intervention** | **Follow up time** | **Number of patients** | **Results** |
| --- | --- | --- | --- | --- | --- | --- |
| Serum albumin | Randomized parallel-group trial | Szeto, 2003 | Oral bicarbonate 0.9g 3x/day | 12 months | 60 (30 treatment, 30 placebo) | - 27.7 ± 4.3 to 28.9 ± 4.6 g/L after 4 weeks (p=0.03) in treatment group but returned to pre-treatment level by 24 weeks. Static in placebo group. Between-group difference non-statistically significant (p=0.6). |
|  | Randomized parallel-group trial | Stein, 1997 | Low alkali (lactate 35 mmol/L) vs high alkali (lactate 40 mmol/L + optional oral sodium bicarbonate averaging 1.4 (0.2) g/day) targeting serum bicarbonate 30 mmol/L | 12 months | 200 (100 low alkali, 100 high alkali; 48 of high alkali receiving sodium bicarbonate) | - 37.2 ± 0.4 to 38.2 ± 0.5 g/L from baseline to one year in low alkali group (p>0.05). - 37.5 ± 0.5 to 37.8 ± 0.4 g/L from baseline to one year in high alkali group (p>0.05). |
| Lean body mass | Randomized parallel-group trial | Szeto, 2003 | Oral bicarbonate 0.9g 3x/day | 12 months | 60 (30 treatment, 30 placebo) | - Stable lean body mass in treatment group. In placebo group, 50.7 ± 7.0 to 48.9 ± 7.1 kg from 0 to 12 weeks (p=0.002). Between-group difference not statistically significant after adjusting for Charlson Comorbidity Index (CCI). |
| Midarm circumference | Randomized parallel-group trial | Stein, 1997 | Low alkali (lactate 35 mmol/L) vs high alkali (lactate 40 mmol/L + optional oral sodium bicarbonate averaging 1.4 (0.2) g/day) targeting serum bicarbonate 30 mmol/L | 1 month | 200 (100 low alkali, 100 high alkali; 48 of high alkali receiving sodium bicarbonate) | - 28.3 ± 0.4 to 28.7 ± 0.4 cm from baseline to one month in low alkali group (p>0.05). - 28.2 ± 0.4 to 29.8 ± 0.4 cm from baseline to one month in high alkali group (p>0.05). |
| nPNA | Randomized parallel-group trial | Szeto, 2003 | Oral bicarbonate 0.9g 3x/day | 12 months | 60 (30 treatment, 30 placebo) | - 1.2 ± 0.3 to 1.3 ± 0.3 g/kg/d (p=0.03) in treatment group. 1.1 ± 0.3 to 1.0 ± 0.3 g/kg/day (p=0.05) in placebo group. - Statistically significant effect of bicarbonate treatment on change in nPNA (p=0.05). |
| PCR | Randomized parallel-group trial | Stein, 1997 | Low alkali (lactate 35 mmol/L) vs high alkali (lactate 40 mmol/L + optional oral sodium bicarbonate averaging 1.4 (0.2) g/day) targeting serum bicarbonate 30 mmol/L | 1 month | 200 (100 low alkali, 100 high alkali; 48 of high alkali receiving sodium bicarbonate) | - 0.9 ± 0.02 to 0.9 ± 0.03 g/kg/d from baseline to one month in low alkali group (p>0.05). - 0.9 ± 0.02 to 0.9 ± 0.02 g/kg/d from baseline to one month in high alkali group (p>0.05). |
| PNA | Non-randomized intervention | Feriani, 2004 | Dialysate bicarbonate 34 vs 39 mmol/L | 24 weeks | 47 (13 on bicarbonate 34, 34 on bicarbonate 39) | - 50.6 ± 7.2 g/day at baseline to 47.1 ± 8.3 g/day at week 24 in dialysate bicarbonate 34 mmol/L group (p>0.05). - 59.7 ± 14.3 g/day at baseline to 58.1 ± 16.8 g/day at week 24 in dialysate bicarbonate 39 mmol/L group (p>0.05). - 60.1 ± 13.2 g/day at baseline to 56.3 ± 13.5 g/day at week 24 in subset of dialysate bicarbonate 39 mmol/L group changing from acidosis to normal acid-base status (p<0.01). |
| Subjective Global Assessment (SGA) | Randomized parallel-group trial | Szeto, 2003 | Oral bicarbonate 0.9g 3x/day | 12 months | 60 (30 treatment, 30 placebo) | - 4.3 ± 0.9 to 4.5 ± 1.0 from 0 to 52 weeks in placebo group. - 4.3 ± 0.9 to 5.2 ±1.0 from 0 to 52 weeks in treatment group. - Treatment had a statistically significant effect on the change in overall SGA score (p<0.001). |
| Triceps skinfold thickness | Randomized parallel-group trial | Stein, 1997 | Low alkali (lactate 35 mmol/L) vs high alkali (lactate 40 mmol/L + optional oral sodium bicarbonate averaging 1.4 (0.2) g/day) targeting serum bicarbonate 30 mmol/L | 1 month | 200 (100 low alkali, 100 high alkali; 48 of high alkali receiving sodium bicarbonate) | - 13.6 ± 0.8 to 14.3 ± 0.8 mm from baseline to one month in low alkali group (p>0.05). - 14.5 ± 0.8 to 16.1 ± 0.9 mm from baseline to one month in high alkali group (p>0.05). |

Abbreviations: nPNA= Normalized protein nitrogen appearance, PCR= Protein catabolic rate, PNA= Protein nitrogen appearance, SGA= Subjective Global Assessment

Conversion factor: Bicarbonate conventional unit (mEq/L) to SI unit (mmol)/L = 1, Lactate conventional unit (mg/dL) to SI unit (mmol/L) = 0.111

Values rounded to one decimal place or one significant figure

**Supplemental Table 8. Serum and plasma bicarbonate, pH, calcium, potassium, and PTH in PD studies**

| **Outcome** | **Study design** | **Study, year** | **Intervention** | **Follow up time** | **Number of patients** | **Results** |
| --- | --- | --- | --- | --- | --- | --- |
| Serum bicarbonate | Randomized parallel-group trial | Liu, 2017 | Oral bicarbonate 1.0 g/day; up-titrated if serum bicarbonate <24, down-titrated if >30 mmol/L | 104 weeks | 28 (15 treatment, 13 placebo) | - 22.6 ± 0.9 mmol/L at baseline vs 26.8 ± 0.8 mmol/L and 22.9 ± 1.2 mmol/L by week 8 and week 104 in placebo group. - 22.4 ± 1.0 mmol/L at baseline vs 30.2 ± 0.9 mmol/L after week 8 (p<0.01) followed by gradual decline up to week 104 in treatment group. - Significantly higher in treatment group at each timepoint. |
|  | Randomized parallel-group trial | Stein, 1997 | Low alkali (lactate 35 mmol/L) vs high alkali (lactate 40 mmol/L + optional oral sodium bicarbonate averaging 1.4 (0.2) g/day) targeting serum bicarbonate 30 mmol/L | 12 months | 200 (100 low alkali, 100 high alkali; 48 of high alkali receiving sodium bicarbonate) | - 20.4 ± 0.6 to 23.0 ± 0.3 mmol/L from baseline to one year in low alkali group (p>0.05). - 20.1 ± 0.5 to 27.2 ± 0.3 mmol/L from baseline to one year in high alkali group (p<0.001). - 27.2 ± 0.3 in high alkali group compared to 23.0 ± 0.3 mmol/L in low alkali group at one year (p<0.001). |
|  | Non-randomized intervention | Feriani, 2004 | Dialysate bicarbonate 34 vs 39 mmol/L | 24 weeks | 47 (13 on bicarbonate 34, 34 on bicarbonate 39) | - At week 24, 27.1 ± 2.1 mmol/L in bicarbonate 34 group vs 25.7 ± 2.8 mmol/L in bicarbonate 39 group (p>0.05). |
| Plasma bicarbonate | Randomized parallel-group trial | Szeto, 2003 | Oral bicarbonate 0.9g 3x/day | 12 months | 60 (30 treatment, 30 placebo) | - Small, statistically significant increase from 22.8 ± 1.7 to 24.7 ± 3.9 mmol/L in placebo group at 4 weeks (p=0.01). - Statistically significant increase from 22.9 ± 1.6 to 27.8 ± 2.6 mmol/L after 12 weeks (p<0.0001) in treatment group, but gradually declined during study period (remained significantly higher than placebo at all timepoints). |
| pH | Randomized parallel-group trial | Stein, 1997 | Low alkali (lactate 35 mmol/L) vs high alkali (lactate 40 mmol/L + optional oral sodium bicarbonate averaging 1.4 (0.2) g/day) targeting serum bicarbonate 30 mmol/L | 12 months | 200 (100 low alkali, 100 high alkali; 48 of high alkali receiving sodium bicarbonate) | - 7.4 ± 0.004 in high alkali group compared to 7.4 ± 0.004 mmol/L in low alkali group at one year (p<0.001). |
|  | Non-randomized intervention | Feriani, 2004 | Dialysate bicarbonate 34 vs 39 mmol/L | 24 weeks | 47 (13 on bicarbonate 34, 34 on bicarbonate 39) | - 7.3 ± 0.02 at baseline to 7.3 ± 0.03 at week 24 in 34 mmol/L group. - 7.3 ± 0.04 at baseline to 7.3 ± 0.04 in 39 mmol/L group (p<0.01). - No significant differences between groups at all timepoints. |
| Calcium | Randomized parallel-group trial | Liu, 2017 | Oral bicarbonate 1.0 g/day; up-titrated if serum bicarbonate <24, down-titrated if >30 mmol/L | 104 weeks | 28 (15 treatment, 13 placebo) | - 2.3 ± 0.2 mmol/L at baseline vs 2.4 ± 1.2 mmol/L by week 104 in placebo group. - 2.3 ± 0.2 mmol/L at baseline vs 2.4 ± 0.1 mmol/L at week 104 in treatment group. |
|  | Randomized parallel-group trial | Stein, 1997 | Low alkali (lactate 35 mmol/L) vs high alkali (lactate 40 mmol/L + optional oral sodium bicarbonate averaging 1.4 (0.2) g/day) targeting serum bicarbonate 30 mmol/L | 12 months | 200 (100 low alkali, 100 high alkali; 48 of high alkali receiving sodium bicarbonate) | - 2.3 ± 0.04 to 2.5 ± 0.3 mmol/L from baseline to one year in low alkali group (p>0.05). - 2.3 ± 0.03 to 2.5 ± 0.02 mmol/ L from baseline to one year in high alkali group (p>0.05). |
| Potassium | Randomized parallel-group trial | Liu, 2017 | Oral bicarbonate 1.0g/day; up-titrated if serum bicarbonate <24, down-titrated if >30 mmol/L | 104 weeks | 28 (15 treatment, 13 placebo) | - 4.3 ± 0.5 mmol/L at baseline vs 4.2 ± 0.4 mmol/L by week 104 in placebo group. - 4.2 ± 0.4 mmol/L at baseline vs 4.2 ± 0.3 mmol/L at week 104 in treatment group. |
|  |  | Szeto, 2003 | Oral bicarbonate 0.9g 3x/day | 12 months | 60 (30 treatment, 30 placebo) | - Potassium concentration remained stable in placebo group. In treatment group changed from 4.4 ± 0.7 mmol/L to 4.0 ± 0.7 mmol/L (p=0.03) at 4 weeks and remained stable after. |
|  |  | Stein, 1997 | Low alkali (lactate 35 mmol/L) vs high alkali (lactate 40 mmol/L + optional oral sodium bicarbonate averaging 1.4 (0.2) g/day) targeting serum bicarbonate 30 mmol/L | 12 months | 200 (100 low alkali, 100 high alkali; 48 of high alkali receiving sodium bicarbonate) | - 4.4 ± 0.1 to 4.0 ± 0.1 mmol/L from baseline to one year in low alkali group (p>0.05). - 4.6 ± 0.1 to 4.2 ± 0.1 mmol/L from baseline to one year in high alkali group (p>0.05). |
| PTH | Randomized parallel-group trial | Stein, 1997 | Low alkali (lactate 35 mmol/L) vs high alkali (lactate 40 mmol/L + optional oral sodium bicarbonate averaging 1.4 (0.2) g/day) targeting serum bicarbonate 30 mmol/L | 12 months | 200 (100 low alkali, 100 high alkali; 48 of high alkali receiving sodium bicarbonate) | - 21.2 ± 2.4 to 15.7 ± 3.0 pmol/L from baseline to one month in low alkali group (p>0.05). - 23.6 ± 2.5 to 17.0 ± 1.9 pmol/L from baseline to one month in high alkali group (p>0.05). |

Abbreviations: PTH= Parathyroid hormone

Conversion factors: Bicarbonate conventional unit (mEq/L) to SI unit (mmol/L) = 1, Calcium conventional unit (mg/dL) to SI unit (mEq/L) = 0.50, Calcium conventional unit (mg/dL) to SI unit (mmol/L) = 0.25, Potassium conventional unit (mEq/L) to SI unit (mmol)/L = 1, Sodium conventional unit (mEq/L) to SI unit (mmol)/L = 1, PTH conventional unit (pg/mL) to SI unit (pmol/L) = 0.106, Lactate conventional unit (mg/dL) to SI unit (mmol/L) = 0.111

Values rounded to one decimal place or one significant figure

**Supplemental Table 9. Summary of meta-analysis findings comparing dialysate bicarbonate > 35 mmol/L to ≤ 35 mmol/L**

**Question:** Dialysate bicarbonate > 35 mmol/L compared to dialysate bicarbonate ≤ 35 mmol/L for patients with kidney failure receiving maintenance HD

| **Certainty assessment** | | | | | | | **№ of patients** | | **Effect** | **Certainty** |
| --- | --- | --- | --- | --- | --- | --- | --- | --- | --- | --- |
| **№ of studies** | **Study design** | **Risk of bias** | **Inconsistency** | **Indirectness** | **Imprecision** | **Other considerations** | **Dialysate bicarbonate >35 mmol/L** | **dialysate bicarbonate ≤35 mmol/L** | **Absolute (95% CI)** |  |
| **Pre-dialysis serum bicarbonate** | | | | | | | | | | |
| 3 | randomized parallel-group trials | serious^a^ | very serious^b^ | not serious | very serious^c^ | none | 55 | 58 | MD **3.5 mmol/L higher** (0.6 lower to 7.7 higher) | ⨁◯◯◯ Very low |
| **Pre-dialysis total calcium** | | | | | | | | | | |
| 3 | randomized parallel-group trials | serious^a^ | not serious | not serious | very serious^c^ | none | 55 | 58 | MD **0.01 mmol/L lower** (0.07 lower to 0.06 higher) | ⨁◯◯◯ Very low |
| **Pre-dialysis potassium** | | | | | | | | | | |
| 3 | randomized parallel-group trials | serious^a^ | not serious | not serious | very serious^c^ | none | 55 | 58 | MD **0.1 mmol/L lower** (0.4 lower to 0.1 higher) | ⨁◯◯◯ Very low |

**CI:** confidence interval; **MD:** mean difference

Explanations

Studies included: Hefzollah 2020, Brady 1998, Lefebvre 1989

a. Some concerns in at least one of the studies in all domains except domain 3 (missing outcome data) and domain 4 (outcome measurement). While there is no study with high risk of bias in any domain and all studies have approximately the same weight, there are some concerns with randomization in two of three studies.

b. Substantial heterogeneity (I^2^ = 94%, Chi-square p-value <0.00001) and confidence intervals of effect estimates do not overlap. Heterogeneity likely arising from difference in interventions between studies (e.g., 30 vs 36 mmol/L, 35 vs 40 mmol/L, 30-35 vs 37-45 dialysate bicarbonate concentrations) and differences in patient populations and era across studies.

c. For continuous outcome, require at least 800 participants included in meta-analysis, but only have 113. Wide confidence intervals; important thresholds not known, but upper and lower confidence limits of estimates cross effect size of approximately 0.5 in both directions.

**Supplemental Table 10. Summary of meta-analysis findings comparing dialysate bicarbonate ≥ 32 mmol/L to ≤ 29 mmol/L**

**Question:** Dialysate bicarbonate ≥ 32 mmol/L compared to dialysate bicarbonate ≤ 29 mmol/L for patients with kidney failure receiving maintenance HD

| **Certainty assessment** | | | | | | | **№ of patients** | | **Effect** | **Certainty** |
| --- | --- | --- | --- | --- | --- | --- | --- | --- | --- | --- |
| **№ of studies** | **Study design** | **Risk of bias** | **Inconsistency** | **Indirectness** | **Imprecision** | **Other considerations** | **Dialysate bicarbonate ≥32 mmol/L** | **dialysate bicarbonate ≤29 mmol/L** | **Absolute (95% CI)** |  |
| **Pre-dialysis ionized calcium** | | | | | | | | | | |
| 3 | Randomized crossover trials | very serious^a^ | not serious | not serious | serious^b^ | none | 74 | 74 | MD **0.0 mmol/L** **lower** (0.03 lower to 0.03 higher) | ⨁◯◯◯ Very low |
| **Post-dialysis ionized calcium**   \| 3 \| Randomized crossover trials \| very serious^a^ \| not serious \| not serious \| serious^b^ \| none \| 74 \| 74 \| MD **0.05 mmol/L** **lower** (0.08 lower to 0.02 lower) \| ⨁◯◯◯ Very low \| \| --- \| --- \| --- \| --- \| --- \| --- \| --- \| --- \| --- \| --- \| --- \|   **Pre-dialysis potassium** | | | | | | | | | | |
| 3 | Randomized crossover trials | very serious^a^ | not serious | not serious | serious^b^ | none | 74 | 74 | MD **0.04 mmol/L lower** (0.2 lower to 0.3 higher) | ⨁◯◯◯ Very low |
| **Post-dialysis potassium** | | | | | | | | | | |
| 3 | Randomized crossover trials | very serious^a^ | Not serious | not serious | serious^b^ | none | 74 | 74 | MD **0.2 mmol/L lower** (0.3 lower to 0.1 lower) | ⨁◯◯◯ Very low |

**CI:** confidence interval; **MD:** mean difference

Explanations

Studies included: Gabutti 2009, Gabutti 2005, Gabutti 2003.

a. High risk of bias in domain 5 (selection bias) in one study. While all studies have approximately the same weight, there are some concerns with randomization in two of three studies.

b. For continuous outcome, require at least 800 participants included in meta-analysis, but only have 74.

Studies from databases/registers **(n = 4221)**

MEDLINE (n = 1665)

Embase (n = 1558)

CENTRAL (n = 798)

Google Scholar (n = 200)

References from other sources **(n = 0)**

Studies included in review **(n = 41)**

Studies excluded **(n = 3311)**

Studies not retrieved **(n = 0)**

Studies assessed for eligibility **(n = 344)**

Studies sought for retrieval **(n = 344)**

Studies screened **(n = 3655)**

**Identification**

References removed **(n = 566)**

Duplicates identified (n = 566)

**Screening**

Studies excluded **(n = 303)**

Review article (n = 48)

Wrong intervention (n = 40)

Abstract only (n = 39)

Investigating serum bicarbonate exposure, not dialysate/oral bicarbonate (n=37)

<20 participants (n = 36)

No outcomes of interest (n = 25)

Wrong comparator (n = 13)

Wrong patient population (n = 13)

Wrong study design (n = 11)

Study protocol (n = 9)

Duplicate/abstract version (n = 6)

Editorial (n = 6)

Letter to the Editor (n = 6)

Case presentation (n = 4)

Commentary (n = 3)

Non-English article (n = 2)

Long term follow-up from previously published results (n = 1)

Paediatric population (n = 1)

Trial registry (n = 1)

Unable to retrieve article (n = 1)

In vitro (n = 1)

**Included**

**Supplemental Figure 1. PRISMA Flow Diagram**

**Supplemental Figure 2. Forest plot of comparison: ≥ 32 mmol/L versus ≤ 29 mmol/L dialysate bicarbonate, outcome: Pre-dialysis ionized calcium.**

**Supplemental Figure 3. Forest plot of comparison: ≥ 32 mmol/L versus ≤ 29 mmol/L dialysate bicarbonate, outcome: Post-dialysis ionized calcium.**

**Supplemental Figure 4. Forest plot of comparison: ≥ 32 mmol/L versus ≤ 29 mmol/L dialysate bicarbonate, outcome: Pre-dialysis potassium.**

**Supplemental Figure 5. Forest plot of comparison: ≥ 32 mmol/L versus ≤ 29 mmol/L dialysate bicarbonate, outcome: Post-dialysis potassium.**

**Appendix A: PRISMA Statement**

| **Section and Topic** | **Item #** | **Checklist item** | **Location where item is reported** |
| --- | --- | --- | --- |
| **TITLE** | | |  |
| Title | 1 | Identify the report as a systematic review. | Pg. 1 |
| **ABSTRACT** | | |  |
| Abstract | 2 | See the PRISMA 2020 for Abstracts checklist. | Pg. 2-3 |
| **INTRODUCTION** | | |  |
| Rationale | 3 | Describe the rationale for the review in the context of existing knowledge. | Pg. 4 |
| Objectives | 4 | Provide an explicit statement of the objective(s) or question(s) the review addresses. | Pg. 4 |
| **METHODS** | | |  |
| Eligibility criteria | 5 | Specify the inclusion and exclusion criteria for the review and how studies were grouped for the syntheses. | Pg. 5 |
| Information sources | 6 | Specify all databases, registers, websites, organisations, reference lists and other sources searched or consulted to identify studies. Specify the date when each source was last searched or consulted. | Pg. 5 |
| Search strategy | 7 | Present the full search strategies for all databases, registers and websites, including any filters and limits used. | Appendix B |
| Selection process | 8 | Specify the methods used to decide whether a study met the inclusion criteria of the review, including how many reviewers screened each record and each report retrieved, whether they worked independently, and if applicable, details of automation tools used in the process. | Pg. 5 |
| Data collection process | 9 | Specify the methods used to collect data from reports, including how many reviewers collected data from each report, whether they worked independently, any processes for obtaining or confirming data from study investigators, and if applicable, details of automation tools used in the process. | Pg. 5 |
| Data items | 10a | List and define all outcomes for which data were sought. Specify whether all results that were compatible with each outcome domain in each study were sought (e.g. for all measures, time points, analyses), and if not, the methods used to decide which results to collect. | Pg. 6, Appendix C |
|  | 10b | List and define all other variables for which data were sought (e.g. participant and intervention characteristics, funding sources). Describe any assumptions made about any missing or unclear information. | Appendix C |
| Study risk of bias assessment | 11 | Specify the methods used to assess risk of bias in the included studies, including details of the tool(s) used, how many reviewers assessed each study and whether they worked independently, and if applicable, details of automation tools used in the process. | Pg. 6 |
| Effect measures | 12 | Specify for each outcome the effect measure(s) (e.g. risk ratio, mean difference) used in the synthesis or presentation of results. | Pg. 7 |
| Synthesis methods | 13a | Describe the processes used to decide which studies were eligible for each synthesis (e.g. tabulating the study intervention characteristics and comparing against the planned groups for each synthesis (item #5)). | Pg. 6-7 |
|  | 13b | Describe any methods required to prepare the data for presentation or synthesis, such as handling of missing summary statistics, or data conversions. | Pg. 7 |
|  | 13c | Describe any methods used to tabulate or visually display results of individual studies and syntheses. | Pg. 6-7 |
|  | 13d | Describe any methods used to synthesize results and provide a rationale for the choice(s). If meta-analysis was performed, describe the model(s), method(s) to identify the presence and extent of statistical heterogeneity, and software package(s) used. | Pg. 6-7 |
|  | 13e | Describe any methods used to explore possible causes of heterogeneity among study results (e.g. subgroup analysis, meta-regression). | N/A |
|  | 13f | Describe any sensitivity analyses conducted to assess robustness of the synthesized results. | N/A |
| Reporting bias assessment | 14 | Describe any methods used to assess risk of bias due to missing results in a synthesis (arising from reporting biases). | N/A |
| Certainty assessment | 15 | Describe any methods used to assess certainty (or confidence) in the body of evidence for an outcome. | Pg. 7 |
| **RESULTS** | | |  |
| Study selection | 16a | Describe the results of the search and selection process, from the number of records identified in the search to the number of studies included in the review, ideally using a flow diagram. | Pg. 7-8, Supplemental Figure 1 |
|  | 16b | Cite studies that might appear to meet the inclusion criteria, but which were excluded, and explain why they were excluded. | N/A |
| Study characteristics | 17 | Cite each included study and present its characteristics. | Pg. 7-8, Supplemental Tables 1 & 2 |
| Risk of bias in studies | 18 | Present assessments of risk of bias for each included study. | Figures 1 & 2, Supplemental Tables 3 & 4 |
| Results of individual studies | 19 | For all outcomes, present, for each study: (a) summary statistics for each group (where appropriate) and (b) an effect estimate and its precision (e.g. confidence/credible interval), ideally using structured tables or plots. | Figures 3-5, Supplemental Figures 2-5 |
| Results of syntheses | 20a | For each synthesis, briefly summarise the characteristics and risk of bias among contributing studies. | Pg. 12-13 |
|  | 20b | Present results of all statistical syntheses conducted. If meta-analysis was done, present for each the summary estimate and its precision (e.g. confidence/credible interval) and measures of statistical heterogeneity. If comparing groups, describe the direction of the effect. | Pg. 12-13, Figures 3-5, Supplemental Figures 2-5 |
|  | 20c | Present results of all investigations of possible causes of heterogeneity among study results. | N/A |
|  | 20d | Present results of all sensitivity analyses conducted to assess the robustness of the synthesized results. | N/A |
| Reporting biases | 21 | Present assessments of risk of bias due to missing results (arising from reporting biases) for each synthesis assessed. | N/A |
| Certainty of evidence | 22 | Present assessments of certainty (or confidence) in the body of evidence for each outcome assessed. | Supplemental Tables 8 & 9 |
| **DISCUSSION** | | |  |
| Discussion | 23a | Provide a general interpretation of the results in the context of other evidence. | Pg. 13-16 |
|  | 23b | Discuss any limitations of the evidence included in the review. | Pg. 13-16 |
|  | 23c | Discuss any limitations of the review processes used. | Pg. 13-16 |
|  | 23d | Discuss implications of the results for practice, policy, and future research. | Pg. 13-16 |
| **OTHER INFORMATION** | | |  |
| Registration and protocol | 24a | Provide registration information for the review, including register name and registration number, or state that the review was not registered. | Pg. 4-5 |
|  | 24b | Indicate where the review protocol can be accessed, or state that a protocol was not prepared. | Pg. 4-5 |
|  | 24c | Describe and explain any amendments to information provided at registration or in the protocol. | N/A |
| Support | 25 | Describe sources of financial or non-financial support for the review, and the role of the funders or sponsors in the review. | Pg. 17 |
| Competing interests | 26 | Declare any competing interests of review authors. | Pg. 17 |
| Availability of data, code and other materials | 27 | Report which of the following are publicly available and where they can be found: template data collection forms; data extracted from included studies; data used for all analyses; analytic code; any other materials used in the review. | Pg. 17 |

*From:*  Page MJ, McKenzie JE, Bossuyt PM, Boutron I, Hoffmann TC, Mulrow CD, et al. The PRISMA 2020 statement: an updated guideline for reporting systematic reviews. BMJ 2021;372:n71. doi: 10.1136/bmj.n71

For more information, visit: <http://www.prisma-statement.org/>

**Appendix B: Search Strategy**

Database: **Ovid MEDLINE(R) ALL <1946 to May 25, 2023>**

| **#** | **Searches** |
| --- | --- |
| 1 | exp Renal Dialysis/ or exp Dialysis Solutions/ or exp Kidney Failure, Chronic/ or exp Peritoneal Dialysis/ or exp Dialysis/ |
| 2 | exp Bicarbonates/ |
| 3 | haemodialy*.mp. |
| 4 | 1 or 3 |
| 5 | 2 and 4 |
| 6 | limit 5 to english language |

Database: **Embase <1974 to May 25, 2023>**

| **#** | **Searches** |
| --- | --- |
| 1 | exp peritoneal dialysis/ or exp dialysis/ or exp dialysis fluid/ or exp peritoneal dialysis fluid/ (230092) |
| 2 | exp hemodialysis fluid/ or exp continuous hemodialysis/ or exp hemodialysis/ (138462) |
| 3 | haemodialysis.mp. or hemodialysis/ (141450) |
| 4 | exp bicarbonate blood level/ (6358) |
| 5 | serum bicarb*.mp. (2337) |
| 6 | dialysate bicarb*.mp. (114) |
| 7 | oral bicarb*.mp. (146) |
| 8 | renal dialys*.mp. (3306) |
| 9 | dialys*.mp. (223381) |
| 10 | peritoneal dialy*.mp. (54836) |
| 11 | hemodialy*.mp. (183903) |
| 12 | 1 or 2 or 3 or 8 or 9 or 10 or 11 (323117) |
| 13 | 4 or 5 or 6 or 7 (7096) |
| 14 | 12 and 13 (1453) |

Database: **EBM Reviews - Cochrane Central Register of Controlled Trials <Inception to May 25, 2023>**

| **#** | **Searches** |
| --- | --- |
| 1 | renal dialy*.mp. (5464) |
| 2 | dialy*.mp. (17227) |
| 3 | hemodia*.mp. (12986) |
| 4 | peritoneal dia*.mp. (2508) |
| 5 | bicarb*.mp. (4445) |
| 6 | serum bicarb*.mp. (318) |
| 7 | oral bicarb*.mp. (38) |
| 8 | dialysate bicarb*.mp. (21) |
| 9 | 1 or 2 or 3 or 4 (21630) |
| 10 | 5 or 6 or 7 or 8 (4445) |
| 11 | 9 and 10 (743) |

Database: **Google Scholar <Inception to May 25, 2023> (6450 results, first 200 sorted by relevance screened)**

| **#** | **Searches** |
| --- | --- |
| 1 | bicarb*, OR dialy* |
| 2 | Exclude patents |

**Appendix C. Data Abstraction Form**

# Research information and study design

## General

**Title**

**Lead author**

**Country**

**Year of publication**

**Funding sources**

**Possible conflicts of interest for study authors**

## Study characteristics

**Aim of study**

Short description of study objective

**Primary outcome**

**Study design**

1. Randomised controlled trial
2. Non-randomised experimental study
3. Prospective cohort study
4. Retrospective cohort study
5. Cross sectional study
6. Other

**Completeness of follow-up (%)**

**Additional notes**

## Participants, intervention/exposure details

**Inclusion criteria**

**Exclusion criteria**

**Total number of participants**

**Number of participants in intervention group**

**Number of participants in control group**

**Duration of dialysis (months/yrs)**

**Intervention group dialysate bicarbonate (HCO3) concentration**

**Control group dialysate bicarbonate (HCO3) concentration**

**Oral sodium bicarbonate prescription**

**Additional notes**

# Patient baseline characteristics

## Demographics and renal profile

**Age (years)**

Mean (SD) or median (IQR)

**Sex (%male)**

**BMI**

**Predialysis DBP**

**Predialysis SBP**

**Postdialysis SBP**

**Smoking (%)**

**Blood flow rate**

**Residual kidney function**

**Single-pool Kt/V**

**eKT/V**

**Total Kt/V**

**Urine volume**

**CrCl**

**Urea Cl**

**eGFR**

**Other**

## Comorbidities (%)

**Coronary artery disease**

**Cerebrovascular disease**

**Peripheral vascular disease**

**Congestive heart failure**

**Hypertension**

**Diabetes**

**COPD**

**Gastrointestinal bleeding**

**Depression/anxiety**

**Cancer**

**Infectious characteristics (e.g. line infections, bacteria, sepsis, hospitalizations)**

**Other**

## Causes of ESRD/Primary renal disease (%)

**Diabetes mellitus**

**Glomerulonephritis (includes Goodpastures, membranous, IgA, FSGS, vasculitis)**

**Hepatorenal syndrome**

**Hypertensive/vascular**

**Inherited/genetic (ADPKD, Alports, Tuberous sclerosis)**

**Interstitial**

**Obstructive uropathy**

**Unknown**

**Other**

## Medications

**ACE-inhibitors or ARB**

**Alpha blockers**

**Betablockers**

**Calcium antagonists**

**EPO**

**Sevelamer**

**Calcium phosphate binders**

**Anticoagulants (i.e. warfarin)**

**ASA/anti-platelets (Plavix)**

**Statins**

**Other**

## Baseline clinical laboratory values

**Albumin**

**Beta2-microglobulin**

**Pre-dialysis bicarbonate**

**Post-dialysis bicarbonate**

**Blood urea nitrogen**

**C-reactive protein**

**Calcium**

**pCO2 (from venous or arterial blood gas)**

**enPCR**

**Ferritin**

**Glucose**

**HDL-cholesterol**

**Hemoglobin**

**Intact parathyroid hormone (iPTH)**

**Lactate**

**LDL-cholesterol**

**nPCR**

**Potassium**

**Sodium**

**Thyroid stimulating hormone**

**Total cholesterol**

**Triglyceride**

**Uric acid**

**Venous pH**

**Arterial pH**

**White blood count**

**Other**

# Outcomes

## Main outcomes of interest

**All-cause mortality**

**Cardiovascular (CV) mortality**

**Infection-related mortality**

**Hospitalizations**

**CV-related hospitalizations**

**Infection-related hospitalizations**

**Fractures**

**Intra-dialytic hypotension**

**Intra-dialytic fluid removal**

**Serum albumin**

**BMI**

**Triceps skinfold thickness**

**Lean body mass**

**Subjective Global Assessment (SGA)**

**Parathyroid hormone (PTH)**

**Pre-dialysis potassium**

**Pre-dialysis phosphate**

**Pre-dialysis serum bicarbonate**

**Other**

## Additional clinical laboratory outcomes

**Beta2-microglobulin**

**Blood urea nitrogen**

**C-reactive protein**

**Calcium**

**pCO2 (VBG or ABG)**

**enPCR**

**Ferritin**

**Glucose**

**HDL-cholesterol**

**Hemoglobin**

**Intact parathyroid hormone (iPTH)**

**Lactate**

**LDL-cholesterol**

**nPCR**

**Potassium**

**Sodium**

**Thyroid stimulating hormone**

**Total cholesterol**

**Triglyceride**

**Uric acid**

**Venous pH**

**Arterial pH**

**White blood count**

**Other**
